# Network analysis of skin tumor progression identifies a rewired genetic architecture affecting inflammation and tumor susceptibility

**DOI:** 10.1186/gb-2011-12-1-r5

**Published:** 2011-01-18

**Authors:** David A Quigley, Minh D To, Il Jin Kim, Kevin K Lin, Donna G Albertson, Jonas Sjolund, Jesús Pérez-Losada, Allan Balmain

**Affiliations:** 1 Helen Diller Family Comprehensive Cancer Center, University of California San Francisco, 1450 Third St, San Francisco, CA 94158, USA; 2 Thoracic Oncology Program, Department of Surgery, University of California San Francisco, 1600 Divisadero St, San Francisco, CA 94143, USA; 3 Department of Laboratory Medicine, University of California San Francisco, 1521 Parnassus Ave, Room C255, Box 0451, San Francisco, CA 94143, USA; 4 Instituto de Biología Molecular y Celular del Cáncer, CSIC/Universidad de Salamanca, Campus M Unamuno s/n, 37007-Salamanca, Spain

## Abstract

**Background:**

Germline polymorphisms can influence gene expression networks in normal mammalian tissues and can affect disease susceptibility. We and others have shown that analysis of this genetic architecture can identify single genes and whole pathways that influence complex traits, including inflammation and cancer susceptibility. Whether germline variants affect gene expression in tumors that have undergone somatic alterations, and the extent to which these variants influence tumor progression, is unknown.

**Results:**

Using an integrated linkage and genomic analysis of a mouse model of skin cancer that produces both benign tumors and malignant carcinomas, we document major changes in germline control of gene expression during skin tumor development resulting from cell selection, somatic genetic events, and changes in the tumor microenvironment. The number of significant expression quantitative trait loci (eQTL) is progressively reduced in benign and malignant skin tumors when compared to normal skin. However, novel tumor-specific eQTL are detected for several genes associated with tumor susceptibility, including IL18 (*Il18*), Granzyme E (*Gzme*), Sprouty homolog 2 (*Spry2*), and Mitogen-activated protein kinase kinase 4 (*Map2k4*).

**Conclusions:**

We conclude that the genetic architecture is substantially altered in tumors, and that eQTL analysis of tumors can identify host factors that influence the tumor microenvironment, mitogen-activated protein (MAP) kinase signaling, and cancer susceptibility.

## Background

Common genetic variants have been shown to affect many complex traits, including cancer susceptibility [1]. However, factors responsible for most of the expected heritable risk of cancer development have not yet been identified. Finding these alleles and isolating the causal polymorphisms is challenging because the heritable component of susceptibility is influenced by many alleles exerting modest effects that may be pleiotropic, epistatic, or context-dependent [2,3]. Mouse models of cancer using inbred strains of a defined genetic background do not recapitulate the genetic heterogeneity inherent in human populations. However, genetically heterogeneous mouse crosses permit analysis of the combinatorial effects of host genetic background and somatic events during tumor evolution, and these crosses have been used to identify polymorphisms that influence tumor susceptibility and progression [4-7]. Analysis of the genetic architecture of gene expression in normal skin from a *Mus spretus*/*Mus musculus *backcross ([SPRET/Ei X FVB/N] X FVB/N, hereafter FVBBX) identified expression quantitative trait loci (eQTL) that influence both structural and functional phenotypes, including hair follicle development, inflammation and tumor susceptibility [8]. A systematic analysis of germline influence on gene expression in benign and malignant skin tumors could identify novel alleles that influence tumorigenesis but are undetectable by analysis of normal tissue. Here we demonstrate that somatic alterations during tumor progression reduce the detectable influence of germline polymorphisms, but alleles that are not relevant in normal tissue are found to influence innate immune responses to skin tumors and are associated with tumor susceptibility.

## Results

### Germline control of gene expression is altered in tumors

Skin tumors were induced on a cohort of 71 FVBBX mice by treatment of dorsal back skin with dimethyl benzanthracene (DMBA) and tetradecanoyl-phorbol acetate (TPA) (see experimental design in Figure S1 of Additional file 1). This treatment induced multiple benign papillomas as well as malignant carcinomas. Gene expression analysis was performed on mRNA extracted from 68 of these papillomas: two papillomas from each of 31 FVBBX mice and a single papilloma from six additional FVBBX mice. Gene expression and DNA copy number analysis was performed on 60 carcinomas that developed on these animals. A second cohort of 28 FVBBX animals (the 'confirmation' cohort) was subsequently generated and treated with the same carcinogenesis protocol as the first set of mice in order to confirm gene expression and eQTL results from the discovery cohort.

**Figure 1:**
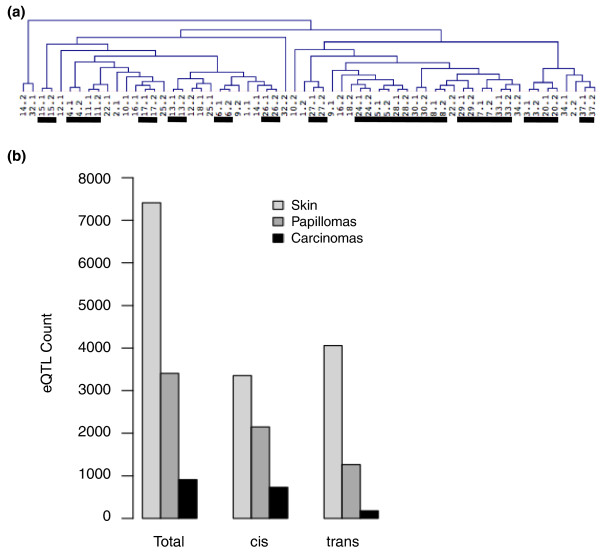
**The influence of germline polymorphisms on gene expression is present but reduced in tumors**. **(a) **Hierarchical clustering of total gene expression from papilloma pairs indicates that germline polymorphisms continue to exert a major effect on gene expression at the benign tumor stage. Bars indicate when both papillomas in a pair are most similar to each other. **(b) **Counts of total, *cis*-, and *trans- *eQTL in skin, papillomas, and carcinomas, showing that overall germline control of gene expression is strongly reduced, particularly for *trans*-eQTL, in malignant carcinomas.

Germline polymorphisms have been shown to influence gene expression in tissues from model organisms and humans [8-13], but it is not clear how this influence is altered during tumor progression. If the germline plays no significant role in tumor gene expression, we would expect papilloma gene expression profiles from the same host to cluster near each other only by chance. Hierarchical clustering of gene expression profiles from papillomas demonstrated that tumors from the same mouse are most similar to each other in 19 of the 31 papilloma pairs (Figure 1a). The highly significant similarity of gene expression from same-host papillomas suggested that germline polymorphisms affect constitutive levels of gene expression in benign tumors (*P *< 0.00001 by permutation; see Materials and methods). The contribution of genetic background to the benign and malignant tumor gene expression profiles was quantified by eQTL analysis. Our previous study of normal skin from the same animals identified almost 8,000 candidate eQTL at ≤10% false discovery rate (FDR). We identified 3,408 candidate eQTL in the 68 papillomas and 912 candidate eQTL in the 60 carcinomas significant at ≤10% FDR (Figure 1b; carcinoma eQTL listed in Table S1 in Additional file 1). At ≤5% FDR we identified 2,175 and 674 candidate eQTL in papillomas and carcinomas, respectively; increasing statistical stringency reduced the number of candidate eQTL but did not change the subsequent results qualitatively, and we report eQTL significant at the 10% FDR level.

The striking reduction in eQTL detected in tumors, particularly in malignant carcinomas, prompted us to investigate reasons why fewer genes are significantly influenced by germline polymorphisms in carcinomas than in normal skin. Of the 7,414 genes with significant eQTL in skin, only 237 are not expressed in tumors, so complete absence of gene expression explains only about 3% of the decrease. EQTLs affecting genes that did not undergo drastic changes (more than two standard deviations from the mean fold-change) in their expression levels were more likely to be conserved between skin and carcinomas (*P *< 7.4e-06, Fisher exact test). Conserved eQTL had significantly stronger statistical significance in normal skin than non-conserved eQTL (*P *< 1e-16, Wilcoxon signed rank test). In normal skin we identified eQTL acting in *cis *(where the locus is physically proximal to the gene it affects) and in *trans *(where the locus is distant from or on another chromosome from the gene it affects) with approximately equal frequencies. The most statistically significant eQTL in skin acted overwhelmingly in *cis*. The *cis*/*trans *proportion detected in tails was 0.8/1, while in papillomas it was approximately 1.5/1, and in carcinomas it was approximately 5.75/1 (exact counts are listed in Table S2 in Additional file 1). We conclude that only very strong eQTL effects carry through from normal skin to affect the malignant carcinomas, and weaker *trans*-acting effects are rarely conserved.

### Somatic events alter the genetic architecture of gene expression in tumors

**Figure 2:**
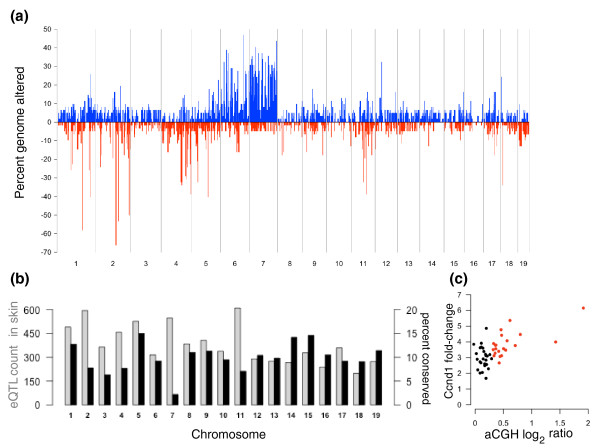
**DNA copy number changes reduce germline influence**. **(a) **Percentage of carcinomas with alterations across the mouse genome; amplifications (blue) plotted above zero, deletions (red) below zero. Chromosome 7 is most frequently amplified. **(b) **Counts of eQTL in skin on autosomal chromosomes (grey bars) compared to percentage of those eQTL conserved in carcinomas (black bars). Left-side scale indicates eQTL counts, right-side scale indicates conservation percentage. Conservation percentage is lowest on chromosome 7. **(c) **Amplification of aCGH probe MouseArray1M2_K17, at chromosome 7, 144.5 Mb, is significantly associated with increased expression of Cyclin D1 in carcinomas compared to matched normal skin. Amplification of this region of distal chromosome 7 accounts for loss of eQTL for Cyclin D1 and other genes in this region.

Changes in the wiring of signaling pathways through epigenetic or genetic alterations may alter the influence of germline polymorphisms on gene expression in transformed cells. We used array comparative genomic hybridization (aCGH) analysis to quantify alterations in tumor DNA copy number. Tumors showed widespread genomic instability (Figure 2a). The most frequent target of large-scale amplification in FVBBX carcinomas was distal chromosome 7, which showed copy number gains in 45% (27 of 60) of carcinomas. Chromosome seven had a markedly smaller percentage of eQTL conserved between skin and carcinomas (2.2%) than other autosomal chromosomes (mean 10%, range 2.2% to -15%; Figure 2b). We identified a significant correlation between amplification of the most distal probe on chromosome 7 and fold-change increases of several genes located near the probe, including *Ccnd1 *(encoding Cyclin D1; *P *= 3.0e-6, mean 10.5-fold up-regulation; Figure 2c). Cyclin D1 amplification or overexpression is an early event in numerous human tumors, and targeted over-expression of *Ccnd1 *drives several mouse models of carcinogenesis [14-16]. Although *Ccnd1 *had a significant *cis*-eQTL in skin (uncorrected *P *= 0.0001, permutation *P = *0.009, *q *< 0.015), this *cis*-eQTL was not detected in papillomas or carcinomas.

DMBA induces a characteristic activating mutation in *Hras1 *[17], which is also located on distal chromosome 7 in the mouse. *Hras1 *also had a significant *cis*-eQTL in skin (uncorrected *P *= 8.7e-5, permutation *P *= 0.013, *q *< 0.02) that was not detected in papillomas or carcinomas. Changes in *Hras1 *mutant gene copy number and/or loss of the normal wild-type allele play a role in tumor progression, and trisomy of chromosome 7 is a common early event in both papillomas and carcinomas, leading to increased copy number of the mutant *Hras1 *allele [18,19]. We conclude that gene copy number alterations on distal chromosome 7 have disrupted the normal genetic control of expression of these target genes.

### Genomic networks are rewired during tumorigenesis

**Figure 3:**
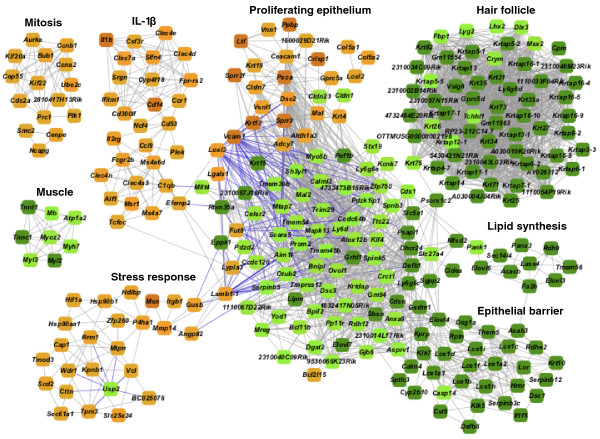
**The progression network for squamous cell carcinomas**. Gene pairs with significantly correlated expression change and change in expression level >2 standard deviations from mean between skin and carcinomas are drawn as nodes. Red nodes indicate increased expression in carcinomas and green nodes indicate decreased expression, with darker color indicating more extreme change. Grey lines connect genes with significant directly correlated change and blue lines indicate significant inverse correlation. The network demonstrates coordinated increases in gene expression motifs associated with mitosis, stress response, epidermal lineage proliferation, and IL1-mediated inflammatory responses. Concomitant decreases are seen in motifs linked to epithelial cell barrier function, hair follicles, lipid biosynthesis, and muscle cells due to major alterations in cell populations in carcinomas compared to normal skin.

Changes in gene expression networks in tumors can result from macroscopic alterations in cellular composition during transformation, or from rewiring of signaling pathways. Coordinated alterations in gene expression from normal to tumor can be visualized as a 'progression network' by combining correlation and differential expression analysis (see Materials and methods; genes used to build this network and fold-change values are listed in Table S3 in Additional file 1). This method identifies functionally related gene sets with significantly correlated changes in expression between two states. The global network constructed in this way is shown in Figure 3 and demonstrates that pathways linked to mitosis, stress responses, and IL1-mediated signaling are seen as distinct network motifs that are up-regulated in carcinomas. Carcinomas result from clonal expansion of initiated epidermal cells, and this is reflected in the down-regulation of motifs related to epithelial barrier, striated muscle, and hair follicles.

**Figure 4:**
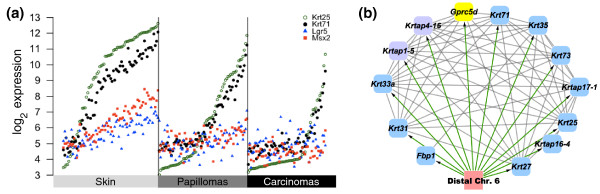
**Re-wiring of the *Lgr5 *hair follicle eQTL network**. **(a) **Gene expression levels of *Krt71*, *Krt25*, *Msx2*, and *Lgr5 *in skin, papillomas, and carcinomas, showing that while *Lgr5 *is significantly correlated with *Msx2 *and *Krt71 *in normal skin, this association is lost during tumor progression. **(b) **A new eQTL network for hair follicle keratins in papillomas where the locus affects *Gprc5d *(yellow node) in *cis *and other genes (blue nodes) in *trans*. Green lines indicate significant influence of eQTL locus on all genes in the network (≤10% FDR); grey lines indicate significant gene-gene correlation.

We previously identified a hair follicle network in normal skin genetically linked to the G-protein coupled receptor gene *Lgr5*, known to mark hair follicle stem cells [8,20]. Papillomas do not produce hair follicles, although they continue to express hair follicle keratins (Figure 4a; Figure S2 in Additional file 1). Although *Lgr5 *is significantly expressed in papillomas and carcinomas, it is not under the control of a *cis*-eQTL in tumors, and also is not linked genetically to the hair follicle correlation network. A papilloma-specific eQTL network including hair follicle keratins and keratin-associated proteins was detected with a shared locus of control on distal chromosome six (Figure 4b), a locus that was not significantly associated with these genes in normal tissue. The G-protein coupled receptor family member *Gprc5d *was the only *cis*-eQTL in the new network (raw *P *= 5.4e-4, permutation *P *= 0.02, *q *= 0.02; linkage map plotted in Figure S3 in Additional file 1). Intriguingly, overexpression of *Gprc5d *promotes hair keratin gene expression, and *Gprc5d *is expressed in *whn *(hairless) nude mice [21], compatible with a role that would only be revealed when normal hair follicle control has been disrupted. These data suggest that the hair follicle stem cell network is significantly rewired during skin tumor development, but the possible role of *Lgr5 *as a marker of tumor initiating cells remains to be determined. We conclude that gene copy number changes, somatic mutations, and alterations in tissue composition in papillomas and carcinomas account for the loss of the *Ccnd1*, *Hras1*, and *Lgr5 *eQTL and likely are responsible for the loss of many other eQTL seen in normal skin.

### Tumor-specific eQTL are associated with susceptibility

Of the 912 transcripts with significant eQTL in carcinoma, 210 did not have a significant eQTL in normal skin (carcinoma eQTL are listed in Table S1 in Additional file 1). Of the 210 eQTL detected only in carcinomas, in 45 cases the transcript was expressed only in carcinomas and not in normal tissue. This may be due to activation of signaling pathways not expressed in normal skin, or by infiltration of transformed epithelium by cell populations from the microenvironment not normally resident in the skin, particularly cells of the innate and adaptive immune systems. Loci that affect the expression of transcripts in tumors but not normal skin may affect tumor susceptibility, but these eQTL would not be evident from analysis of normal tissue. To identify genes with tumor-specific eQTL that were associated with susceptibility, we identified genes that were significantly differentially expressed when contrasting papillomas from resistant and susceptible animals (FDR ≤5%). Genes were considered of interest if they had expression in papillomas significantly associated with susceptibility and also had a tumor-specific eQTL.

**Figure 5:**
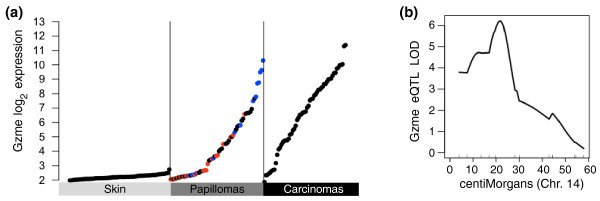
**Granzyme E alleles are associated with su sceptibility**. **(a) **Log_2 _expression of *Gzme *in skin, papillomas, and carcinomas. *Gzme *mRNA is not detected in normal skin, and its level of expression is highest in papillomas from mice that are relatively resistant to papilloma development. Papillomas from resistant animals are plotted as blue circles, susceptible animals as red circles. **(b) **Expression of *Gzme *in papillomas and carcinomas is under germline genetic control. LOD plot for *Gzme *carcinoma eQTL significance on chromosome 14.

Twenty-nine genes met these criteria (listed in Table 1). Of these genes, the serine protease Granzyme E (*Gzme*) showed the largest induction in papillomas from resistant mice. *Gzme *is expressed in granules released by cytotoxic T lymphocytes and together with perforin can destroy pathogen-infected or transformed cells [22,23]. *Gzme *was expressed at background levels in normal FVBBX skin, but at a range of detectable levels in papillomas and carcinomas (Figure 5a). The tumor-specific *cis*-eQTL for *Gzme *peaked at chromosome 14, 51 Mb in papillomas and carcinomas (raw *P *= 6.6e-7, permutation *P *< 0.001, *q *< 0.001; Figure 5b). Mice heterozygous at the eQTL locus (that is, with *Gzme *alleles inherited from both FVB/N and SPRET/Ei) had higher expression of *Gzme *in papillomas and carcinomas than mice homozygous for FVB/N at this allele. Although (as previously reported [8]) classical QTL analysis of papilloma counts for these FVBBX mice did not identify a locus significant after multiple test correction, the strongest linkage was to markers on chromosome 14, peaking at 62 Mb (linkage map plotted in Figure S4 in Additional file 1). The SPRET/Ei allele was protective at this locus, in agreement with the direction of the *Gzme *eQTL and susceptibility results. We conclude that *Gzme *is a strong candidate modifier of papilloma susceptibility based on genetic control of gene expression in tumor tissue, higher levels of expression in papillomas from resistant mice carrying the SPRET/Ei allele, and the documented biological activity of granzymes in killing of potential tumor cells.

**Table 1:** Genes with novel eQTL in tumors that are also associated with susceptibility

Symbol	Probe	Chr.	Mb	Fold change	SAM *q*-value	Higher in	Higher genotype	eQTL chr.	eQTL Mb
*Gzme*	1421227_at	14	56.7	*-16.67*	<0.01	Resist.	Het.	14	41
*Gzme*	1450171_x_at	14	56.7	*-7.69*	<0.01	Resist.	Het.	14	41
*Mnda*	1452349_x_at	1	175.8	*-2.94*	4.31	Resist.	Het.	1	169
*2310005E10Rik*	1453173_at	6	34.3	*-2.27*	3.02	Resist.	Het.	6	32
*Ddx6*	1439122_at	9	44.4	*-1.82*	4.31	Resist.	Hom.	9	34
*Spry2*	1421656_at	14	106.3	*-1.39*	3.02	Resist.	Het.	14	94
*Kctd3*	1436811_at	1	190.8	1.16	4.31	Susc.	Hom.	1	187
*Map2k4*	1451982_at	11	65.5	1.16	1.38	Susc.	Hom.	11	101
*Ssr1*	1441327_a_at	13	38.1	1.18	3.02	Susc.	Hom.	10	106
*Ndst2*	1417931_at	14	21.5	1.21	3.02	Susc.	Hom.	14	19
*Ppih*	1429832_at	4	119.0	1.23	2.02	Susc.	Hom.	5	44
*1810063B07Rik*	1427905_at	14	20.9	1.23	1.38	Susc.	Hom.	14	23
*Psme3*	1418078_at	11	101.2	1.25	3.02	Susc.	Hom.	4	75
*Tardbp*	1436318_at	4	148.0	1.25	2.02	Susc.	Hom.	1	187
*BC003266*	1449189_at	4	126.9	1.25	<0.01	Susc.	Hom.	4	121
*Acbd6*	1452601_a_at	1	157.4	1.26	2.02	Susc.	Het.	9	116
*2810457I06Rik*	1436805_at	9	40.8	1.26	0.93	Susc.	Het.	9	34
*Dhdds*	1450654_a_at	4	133.5	1.26	<0.01	Susc.	Hom.	4	141
*Nrd1*	1424391_at	4	108.7	1.28	0.93	Susc.	Hom.	10	118
*Nme6*	1448574_at	9	109.7	1.3	0.93	Susc.	Het.	9	102
*Sept8*	1426802_at	11	53.3	1.35	2.02	Susc.	Hom.	4	106
*Hyls1*	1431315_at	9	35.4	1.38	1.38	Susc.	Het.	9	34
*Pus3*	1418491_a_at	9	35.4	1.42	0.93	Susc.	Het.	9	34
*C230096C10Rik*	1436709_at	4	138.9	1.43	1.38	Susc.	Hom.	4	141
*Creg1*	1415947_at	1	167.7	1.46	2.02	Susc.	Hom.	1	160
*Asah3l*	1451355_at	4	86.5	1.51	0.33	Susc.	Hom.	13	1
*Rdh11*	1449209_a_at	12	80.3	1.57	3.02	Susc.	Het.	6	32
*Mtap2*	1434194_at	1	66.2	1.81	1.38	Susc.	Hom.	10	102
*Tslp*	1450004_at	18	33.0	2.34	2.02	Susc.	Het.	5	138
*Il18*	1417932_at	9	50.4	2.34	<0.01	Susc.	Het.	9	34

Genes that satisfy two conditions: rewired or novel eQTL in tumors compared to normal skin and significant differential expression in papillomas when tumors from resistant and susceptible mice are compared. 'Chr.' and 'Mb' indicate gene location; 'Fold change' indicates differential expression between resistant and susceptible; 'SAM *q*-value' indicates differential expression significance; 'Higher in' indicates whether the gene was higher in resistant (Resist.) or susceptible (Susc.) animals; 'Higher genotype' indicates whether heterozygous (Het.) or homozygous (Hom.) alleles were associated with higher expression; 'eQTL chr.' and 'eQTL Mb' indicate peak eQTL linkage.

Higher expression of several other genes was associated with resistance, including Sprouty homolog two (*Spry2*), a negative regulator of Ras/mitogen-activated protein kinase (MAPK) signaling (raw *P *= 7.6e-8, permutation *P *< 0.01, *q *= 0.03). *Spry2 *was also expressed at very low levels in normal skin, but was expressed at elevated levels in tumors. The DMBA/TPA model of carcinogenesis is driven by oncogenic signaling through the Ras pathway, and it is plausible that mice with higher constitutive levels of *Spry2 *expression in tumors would show greater resistance to tumorigenesis.

Some genes associated with susceptibility are expressed in normal skin but are only under germline control in tumors. The IL1 family member IL18 (*Il18*) was expressed in skin and tumor samples, but only in carcinomas did *Il18 *have a strong *cis*-eQTL, with higher expression in papillomas from susceptible animals and when a SPRET/Ei allele was present (raw *P *= 2.6e-8, permutation *P *< 0.001, *q *= 0.001). Higher levels of the kinase *Map2k4 *(also called *Mek4 *or *Mkk4*) are also associated with increased susceptibility, and this gene is under germline control only in tumors (raw *P *= 3.5e-5, permutation *P *= 0.005, *q *= 0.014). A recent report has shown that FVB mice with a skin-specific knockout of *Map2k4 *are resistant to the DMBA/TPA tumorigenesis protocol, consistent with our eQTL analysis [24].

### Perturbation from normal expression is controlled primarily in *trans*

The tumor eQTL analysis described above was based on steady state levels of all transcripts detected in tumors. The availability of matched normal skin and tumor tissue enabled us to ask whether the degree of perturbation of transcript levels in the tumors, as opposed to their steady state levels, is under germline control. We performed an eQTL analysis on the gene expression changes when comparing the same probe in matched normal skin and carcinomas. Including only probes that were expressed above background in both skin and carcinomas and that did not have a significant eQTL in tail or carcinoma analysis based on steady-state levels, we identified 55 significant eQTL. In contrast to carcinoma eQTL, which acted almost exclusively in *cis*, 80% of these novel 'perturbation eQTL' acted in *trans *(44 of 55; listed in Table S4 in Additional file 1). A recent report investigating gene expression changes in human cell lines in response to ionizing radiation demonstrated that loci associated with response overwhelmingly acted in *trans *[25]. It is possible, therefore, that major perturbations of gene expression as a result of DNA damage or tumor development are controlled indirectly through the influence of *trans*-acting regulatory factors (for example, transcription factors) rather than through widespread influence on transcription levels of individual genes.

### Confirmation of tumor eQTL

Of the 912 transcripts with significant eQTL in the discovery carcinoma eQTL data set, 560 were significant in the confirmation cohort at a 5% FDR. The number of samples in the confirmation cohort was relatively small (*N *= 28), and it is possible that more predicted eQTL would have been confirmed with a more highly powered study. These eQTL were mostly *cis*-eQTL and included the eQTL affecting *Gzme *and *Il18 *expression (Figure S5 in Additional file 1; replication results listed in Table S1 in Additional file 1).

## Discussion

The past few years have witnessed an explosion in genome-wide association studies of cancer susceptibility in human populations. While these studies have revealed many new genetic variants that influence cancer risk, each variant is predicted to have a very small effect on susceptibility, and most heritable factors influencing risk remain to be discovered [26]. Some risk is conferred by rare variants with large effects, such as the *BRCA1*/*BRCA2 *mutations that increase breast cancer susceptibility. Rare variations cannot be detected by genome-wide association studies, which analyze only common (typically >5% minor allele frequency) alleles. Epistatic interactions between common alleles may also contribute to cancer risk. The latter model is supported by studies of mouse models of cancer susceptibility, which have demonstrated that common alleles interact in a complex fashion to influence risk [27]. However, even in mouse models that combine defined inbred strains with dramatically different tumor susceptibilities under well-controlled environmental conditions, classical mapping studies have not identified the majority of the risk factors [28].

The realization that cancer susceptibility is an emergent property of the combinatorial effects of many genes necessitated the development of more complex network-based approaches that integrate classical genetics with gene expression analysis in normal and transformed tissues. We have previously used a systems genetics approach to analyze how gene expression networks in normal whole skin vary between animals that are susceptible or resistant to skin papilloma development. This approach led to identification of pathways controlling mitosis, inflammation and tissue remodeling in normal skin that affect individual susceptibility [8]. In the present study we have focused on analysis of the rewiring of these normal gene expression networks during development of benign and malignant tumors from the same heterogeneous population of inter-specific backcross mice.

Our data illuminate the dynamic changes in cell populations, both tumor-derived and host-derived, that accompany the evolution of solid tumors. Genomic networks in squamous cell carcinomas are profoundly deregulated compared to normal epithelium and benign papillomas, reflecting major changes in gross tissue organization and signaling. Allelic variation continues to influence tumor gene expression, although this influence is reduced by the somatic alterations accompanying progression. The strongest reduction in tumors is seen in eQTL that act in *trans*, possibly due to genomic instability leading to alterations in transcription factor-mediated control of gene expression and the tissue-specific nature of *trans*-eQTL. eQTL under the control of *cis*-acting elements in general have stronger effects than *trans*-eQTL, and they may be more robust in the face of somatic genetic changes because the causal variant affects the gene directly. A recent study compared eQTL detected in hematopoietic cells at four stages of differentiation and demonstrated that many eQTL are unique to each state, and *trans*-eQTL are less likely to be conserved between differentiation states than *cis*-eQTL [29]. *Trans*-eQTL were detected in all four states.

We have also identified 'perturbation eQTL', which measure the degree to which changes in levels of gene expression between normal and transformed states are under genetic control. These eQTL reflect genetic control of the changes that occur in response to exogenous damage. In contrast to the steady state eQTL that are mainly *cis*-acting, perturbation eQTL act primarily in *trans*, similar to a scenario recently described for human lymphoblastoid cells subjected to ionizing radiation [25]. The mechanistic basis for these observations remain to be determined by isolation and analysis of the *trans*-acting factors responsible for these effects.

Genetic and gene expression analyses of tumors reveals features that cannot be detected by analysis of normal tissues, such as the *cis*-eQTL controlling expression of *Il18*, *Gzme*, *Map2k4*, and *Spry2 *in tumors but not normal skin. *Il18 *has an important and complex role in inflammatory and immune responses; it has been reported to have both tumor-promoting and anti-tumor activities in different contexts [30]. It remains to be determined whether the gain of germline influence over *Il18 *expression reflects a change in cell populations or a modification in cell-autonomous signaling. The presence of a tumor-specific eQTL for *Il18 *may reflect differences in the relative proportions of epithelial and inflammatory cells in the tumors, or may be due to rewiring of *Il18 *signaling during progression.

Unlike *Il18*, *Gzme *expression is not detectable in normal skin, and appears in papillomas and carcinomas concomitantly with the influx of innate immune cells. Mice with higher levels of *Gzme *within their papillomas were relatively resistant to papilloma development, in agreement with a protective role for *Gzme*, and possibly other granzymes within this gene cluster, in tumor development. In contrast, mice with high levels of *Il18 *in their papillomas were most susceptible to tumor development. These data suggest that innate immune cell responses against tumors are stronger in animals that carry the SPRET/Ei allele at the *Gzme *locus, due to a polymorphism resulting in higher *Gzme *expression. This analysis also suggests opposing roles in tumor susceptibility for *Map2k4 *and *Spry2*, genes that exert opposite effects on mitogen-activated protein kinase (MAPK) signaling.

Tumor signaling can be rewired due to oncogenic mutations or loss of tumor suppressor genes, possibly revealing activity of a germline polymorphism that is not evident in normal tissue. The identification of susceptibility genes by a combination of genetic and gene expression analysis of tumors highlights the power of this approach to elucidate the genetic architecture of cancer susceptibility. A combination of genetic and gene expression analysis of human tumors will complement genetic association methods and may identify additional susceptibility factors that cannot be detected using classical methods.

## Materials and methods

### Mouse models, gene expression, and aCGH

FVBBX mice were generated and treated with DMBA/TPA as described in [8]. Gene expression was measured with the Affymetrix Mouse Genome 430 2.0 microarray, Affymetrix annotation release 30. Microarray probesets where all 11 probes did not hybridize to an annotated Refseq gene were eliminated from analysis. Animals sensitive to papilloma tumorigenesis were defined as >7 papillomas after 20 weeks of treatment (*N *= 22), resistant as <2 papillomas at that time point (*N *= 11). The confirmation cohort of FVBBX mice was generated and treated by the same protocol, with genotypes and gene expression measured as described above. Genomic amplification/deletion was measured with a 2,504 probe aCGH system using log_2 _± 0.3 cutoffs for amplification/deletion [31]. Percentage of the genome altered was calculated by dividing each chromosome into 1,000,000 equally spaced bins and calling each bin amplified or deleted depending on the status of the most physically proximal probe for which a measurement was available. Statistical analysis was performed with the R package [32].

### Statistical analysis of gene expression

Permutation analysis of hierarchical clustering was performed by first calculating the distance matrix for sample gene expression using all present genes, counting cases where the closest papilloma to a given sample was from the same mouse (N_observed_). We performed 10,000 permutations of the sample labels and calculated N_perm _in the same manner, reporting the number of times N_perm _≥ N_observed _divided by 10,000. Differential expression was analyzed with the Significance Analysis of Microarrays algorithm [33]. Correlation was defined as significant at the 5% alpha level using the experiment-wise genome-wide error rate permutation method as described in [34]. To calculate the tumor progression network, skin and carcinoma microarrays were normalized together and genotype-matched skin expression was subtracted from tumor expression. Mean fold-change values were approximately normally distributed. Highly significant change for progression networks was defined as >2 standard deviations from the global mean change (*N *= 926). Significant correlation in fold-change was assessed at the 5% genome-wide level using the genome-wide error rate method as described in [34]. All significantly correlated pairs of probes with highly significant fold-change in expression and membership in a network clique of size 3 or greater were plotted. Correlation networks were drawn using Cytoscape [35]. Microarray results have been deposited in the Gene Expression Omnibus under accession number [GEO:GSE21264].

### eQTL analysis

Pairs of papillomas from the same animal were combined for eQTL analysis using the mean expression for each probeset. eQTL were identified by linear regression as previously described [8]. Briefly, corrected eQTL *P*-values were calculated by storing the lowest observed *P*-value *p*_*minimal-obs *_across all 230 SNPs and generating 1,000 shuffled genotypes, calculating *p*_*minimal-perm *_for each permutation, and reporting the rank of *p*_*minimal-obs *_in the sorted set of *p*_*minimal-perm *_divided by 1,000. The distribution of corrected *P*-values was used as input to Storey's QVALUE software [36] to calculate FDR *q*-values. Candidate *cis*-eQTL were defined as loci within 30 Mb of the gene they were predicted to affect (qualitatively similar results were obtained with windows of 20 and 40 Mb). Interval mapping was performed with R/QTL [37]. The 912 carcinoma eQTL from the discovery cohort were tested in the confirmation dataset by linear regression of the loci and gene expression values. The distribution of 912 confirmation *P*-values was used with QVALUE to calculate *q*-values for confirmation results.

## Abbreviations

aCGH: array comparative genomic hybridization; DMBA: dimethyl benzanthracene; eQTL: expression quantitative trait locus/loci; FDR: false discovery rate; FVBBX: [SPRET/Ei X FVB/N] X FVB/N; IL: interleukin; TPA: tetradecanoyl-phorbol acetate.

## Authors' contributions

The study was conceived and supervised by AB. Bioinformatics analysis was performed by DAQ. RNA and DNA extraction was performed by IK, KKL, and MDT. JPL contributed mice and tumor samples and KKL performed array analysis for the confirmation study. Taqman assays were performed by JS. Array CGH data were provided by DGA. The paper was written by DAQ and AB.

## Supplementary Material

Additional file 1 **Additional figures and tables**. A schematic overview of the experiment, additional detailed figures supporting the eQTL analysis, a table listing eQTL detected in carcinomas, a table detailing *cis*- and *trans*-eQTL counts, a table listing genes altered more than two standard deviations from the mean in carcinomas compared to matched normal skin, and a table listing perturbation eQTL identified. Click here for file
