# Supplementary material for: Network analysis of skin tumor progression identifies a rewired genetic architecture affecting inflammation and tumor susceptibility
Source: Genome Biol. 2011 Jan 18;12(1):R5. doi: 10.1186/gb-2011-12-1-r5 (PMC3091303; doi:10.1186/gb-2011-12-1-r5)
Supplement: Additional file 1 — Additional figures and tables. A schematic overview of the experiment, additional detailed figures supporting the eQTL analysis, a table listing eQTL detected in carcinomas, a table detailing cis- and trans-eQTL counts, a table listing genes altered more than two standard deviations from the mean in carcinomas compared to matched normal skin, and a table listing perturbation eQTL identified. [file gb-2011-12-1-r5-S1.DOC]

**Network Analysis of Benign and Malignant Skin Tumors Identifies a Rewired Genetic Architecture Affecting Host-Tumor Interactions**

David A. Quigley^1^, Minh D. To^1,2^, Il-Jin Kim^1^, Kevin K. Lin^1^, Donna G. Albertson^1,3^, Jonas Sjolund^1^, Jesús Pérez-Losada^4^ & Allan Balmain^1*^

^1^*Hellen Diller Family Comprehensive Cancer Center, University of California San Francisco, 1450 Third St. San Francisco CA 94158, USA* ^2^*Thoracic Oncology Program, Department of Surgery, University of California San Francisco, 1600 Divisadero St., San Francisco, CA 94143, USA* ^3^*Department of Laboratory Medicine, University of California San Francisco, 1521 Parnassus Ave. Room C255 Box 0451, San Francisco, CA 94143, USA* ^4^*Instituto de Biología Molecular y Celular del Cáncer, CSIC/Universidad de Salamanca, Campus M. Unamuno s/n, 37007-Salamanca, Spain*

**Correspondence: abalmain@cc.ucsf.edu, (415) 502-4192*

**SUPPLEMENTARY FIGURES**


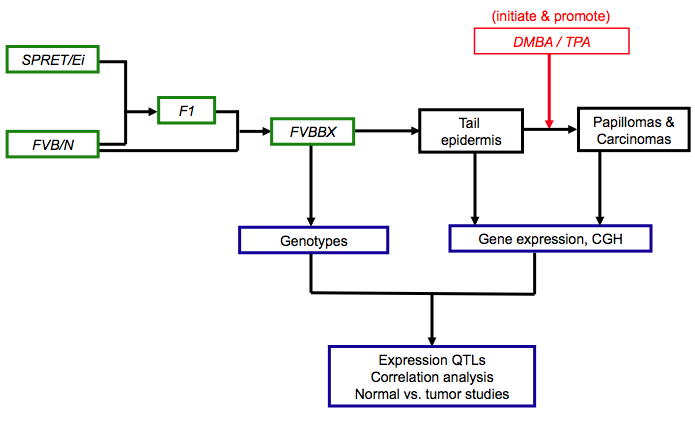


**Figure S1: Overview of experimental design**

FVBBX mice are generated through a back-cross of SPRET/Ei and FVB/N strains. Back skin is treated with the standard DMBA / TPA protocol as described in [38]. Total mRNA from untreated tail epidermis, papillomas, and carcinomas was used for microarray analysis. Analysis of genomic and eQTL networks in tail mRNA was previously published in [38]. DNA from tails was used for genotyping, and DNA extracted from carcinomas was used with tail DNA for CGH analysis.


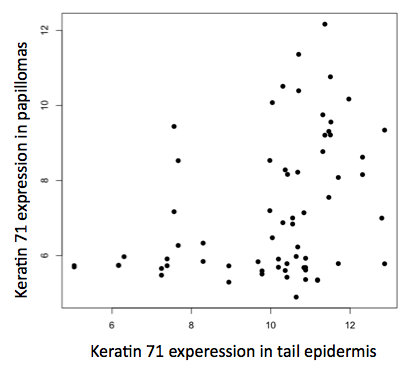


**Figure S2: Keratin 71 expression in tail epidermis and matched (same animal) papillomas.** Raw log_2_ expression values for *Krt71* from same-animal matched tail and papillomas. Note that expresion in papillomas is not significantly correlated with expression in tails. Although some tail samples with high expression still maintain high expression in papillomas (points plotted in the upper right quadrant), almost all papillomas show weaker *Krt71* expression than tails (points plotted along bottom). Very similar results hold for other hair follicle keratins (data not shown).


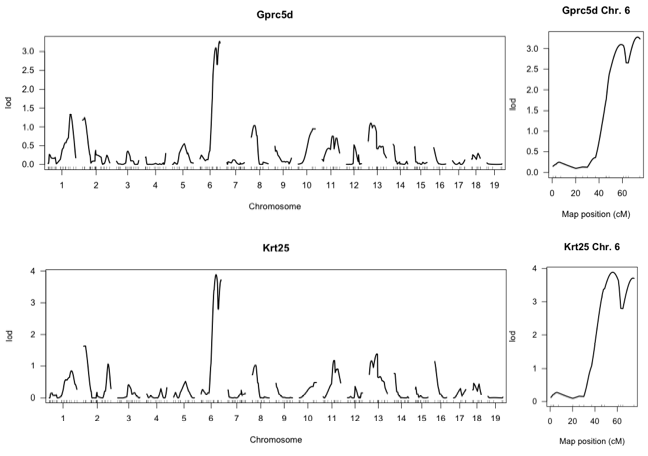


**Figure S3: Interval mapping for Gprc5d and Krt25 eQTL.** Numerous keratins that make up the structure of the hair follicle have strong eQTL on distal chromosome six in papillomas. The reason for the "double peak" of the LOD peak is not clear; it may suggest a complex locus where more than one nearby allele is affecting gene expression, or it may a result of the relatively low number of genotypes (N=37) used to generate these eQTL results.

A B


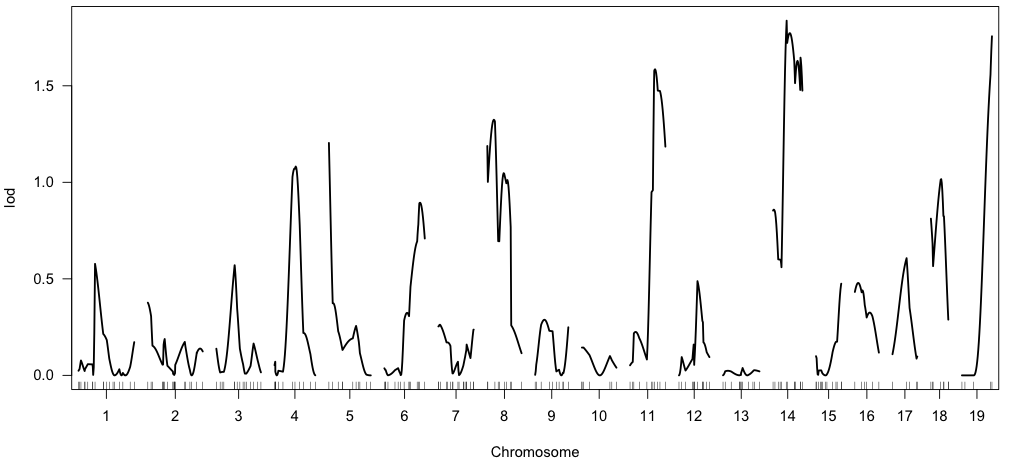


**Figure S4: QTL analysis of papilloma number after 20 weeks of treatment in 71 FVBBX animals.** Although no locus is significant at a 5% GWER level (required LOD: 2.56) the highest LOD peak is on chromosome 14 at 62 Mb (A). Mice with a SPRET/Ei allele have lower numbers of papillomas (B). *Gzme* is located on chromosome 14 at 56 Mb.

| **A** | **B** |
| --- | --- |

**Figure S5: Confirmation of *Gzme* and *Il18* eQTLs**

Figures show measured expression values for (a) *Gzme* (uncorrected P-value = 4.6e-4) and (b) *Il18* (uncorrected P-value = 4.9e-4) in a replication set of 28 carcinomas (see supplementary Methods), divided by genotype at the locus *cis* to each gene. The most significant each eQTL was found at the same loci as those identified in the discovery set of 60 carcinomas. As in the discovery set, mice heterozygous at the gene loci have higher expression of *Gzme* and *Il18,* respectively.


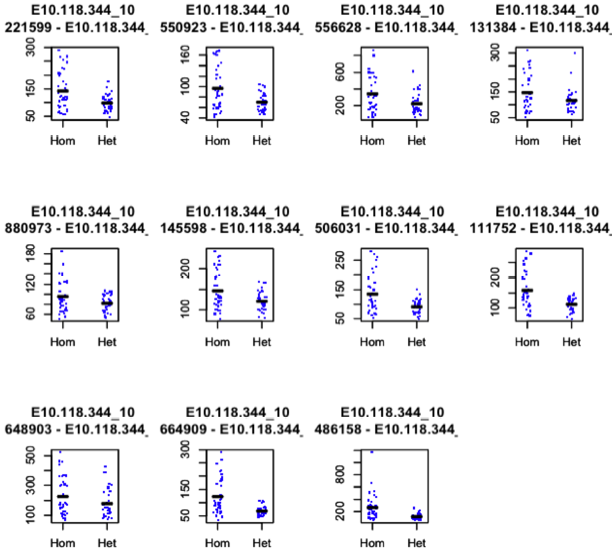


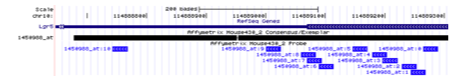


**Figure S6: Analysis of SNP effects on probe hybridization.**

A) Raw probe expression values for 11 probes in *Lgr5* probeset 1450988_at. SNPs are present in probe nine at 114,889,007 and 114,889,025 and in probe 10 at 114,888,763 nucleotides (mm9 assembly). These are the lower middle and lower right raw plots, respectively. B) Location of *Lgr5* probesets for M430v2 microarray, illustrated with the UCSC Genome browser (genome.ucsc.edu). This figure is referenced by the text associated with **Supplementary Table 2.**

**SUPPLEMENTARY TABLES**

**Table S1**

Probes with eQTL significant at ≤ 10% FDR in carcinomas.

"**SNP**": indicates the single most significant locus for the probe.

"**raw**": Observed *P* value for carcinoma eQTL in discovery cohort

"**perm**": permutation *P* values after 1000 permutations.

"**FF**", "**FS**": Mean expression values for mice homozygous FVB/N and heterozygous FVB/N and Spret/Ei

"**Cons**": Probes with a significant eQTL conserved from normal skin .

"**Conf Q**": Q value of this exact SNP-probe pair in the confirmation cohort.

| **Gene** | **Probe** | **SNP** | **raw** | **perm** | **FF** | **FS** | **Cons** | **Conf Q** |
| --- | --- | --- | --- | --- | --- | --- | --- | --- |
| Pdxdc1 | 1452705_at | E16.018.064_10 | 4.90E-17 | 0 | 8.18 | 7.35 | Y | 4.28E-03 |
| Uso1 | 1424274_at | E05.085.774_10 | 5.28E-17 | 0 | 9.53 | 8.72 | Y | 8.05E-06 |
| Gdi2 | 1436016_x_at | E13.001.439_10 | 6.21E-17 | 0 | 11.82 | 11.11 | Y | 2.90E-04 |
| Adpgk | 1451079_at | E09.052.894_10 | 2.79E-16 | 0 | 5.26 | 6.52 | Y | 3.62E-07 |
| Gdi2 | 1435898_x_at | E13.001.439_10 | 9.56E-16 | 0 | 12.25 | 11.5 | Y | 3.23E-04 |
| Ythdc1 | 1456699_s_at | E05.085.774_10 | 6.20E-15 | 0 | 8.98 | 8.31 | Y | 8.41E-04 |
| Rchy1 | 1432144_a_at | E05.085.774_10 | 2.68E-14 | 0 | 8.65 | 7.83 | Y | 1.10E-04 |
| Rex2 | 1438237_at | E04.141.582_10 | 2.92E-14 | 0 | 2.98 | 4.69 | Y | 2.84E-06 |
| Rap2b | 1448885_at | E03.066.854_10 | 5.56E-14 | 0 | 6.76 | 8.93 | Y | 5.32E-05 |
| Plau | 1422139_at | E14.019.954_10 | 3.11E-13 | 0 | 5.86 | 8.83 | Y | 7.40E-04 |
| Poldip3 | 1452709_at | E15.087.204_10 | 3.21E-13 | 0 | 8.34 | 7.69 | Y | 7.14E-03 |
| Hyou1 | 1423290_at | E09.034.366_10 | 7.10E-13 | 0 | 6.82 | 7.74 | Y | 2.03E-02 |
| Aph1b | 1456500_at | E09.052.894_10 | 7.19E-13 | 0 | 5.42 | 6.32 | Y | 1.12E-04 |
| Stk25 | 1416770_at | E01.083.652_10 | 1.35E-12 | 0 | 7.58 | 8.43 | Y | 2.53E-05 |
| Qrich1 | 1426289_at | E09.102.375_10 | 2.46E-12 | 0 | 8.8 | 8.04 | Y | 9.77E-05 |
| Gstp1 | 1449575_a_at | E19.005.316_10 | 3.05E-12 | 0 | 11.03 | 11.87 | Y | 2.75E-04 |
| Arsk | 1453109_at | E13.056.787_10 | 4.45E-12 | 0 | 4.09 | 5.36 | Y | 5.61E-03 |
| Tmem33 | 1452053_a_at | E05.085.774_10 | 5.77E-12 | 0 | 8.34 | 7.52 | Y | 3.01E-03 |
| Cox18 | 1415710_at | E05.085.774_10 | 7.89E-12 | 0 | 8.03 | 7.38 | Y | 4.37E-04 |
| Rpl17 | 1453752_at | E18.077.468_10 | 1.10E-11 | 0 | 7.23 | 9.03 | Y | 6.87E-04 |
| BC003993 | 1438278_a_at | E02.085.273_10 | 1.13E-11 | 0 | 6.02 | 7.58 | Y | 4.71E-04 |
| Aco2 | 1436934_s_at | E15.087.204_10 | 1.20E-11 | 0 | 8.75 | 7.84 | Y | 1.78E-04 |
| 2310005E10Rik | 1453173_at | E06.032.288_10 | 1.35E-11 | 0 | 6.69 | 8.54 | N | 9.25E-06 |
| Plau | 1422138_at | E14.019.954_10 | 1.49E-11 | 0 | 5.48 | 7.91 | Y | 1.14E-03 |
| Fbxo22 | 1426592_a_at | E09.052.894_10 | 1.64E-11 | 0 | 7.03 | 6.57 | Y | 2.38E-05 |
| Dck | 1439012_a_at | E05.085.774_10 | 1.79E-11 | 0 | 7.91 | 6.84 | Y | 1.22E-03 |
| Polr3f | 1429686_at | E02.137.900_10 | 2.24E-11 | 0 | 6.31 | 5.75 | Y | 1.19E-01 |
| Ntan1 | 1423538_at | E16.018.064_10 | 2.30E-11 | 0 | 8.68 | 9.4 | N | 1.28E-04 |
| Rpo1-3 | 1451120_at | E05.138.555_10 | 2.60E-11 | 0 | 10.61 | 9.74 | Y | 2.71E-03 |
| Dck | 1449176_a_at | E05.085.774_10 | 2.98E-11 | 0 | 5.92 | 5.09 | Y | 1.73E-03 |
| Ppat | 1452831_s_at | E05.085.774_10 | 4.20E-11 | 0 | 6.63 | 5.89 | Y | 1.25E-02 |
| 6030458C11Rik | 1433860_at | E15.024.918_10 | 5.01E-11 | 0 | 7.22 | 6.48 | Y | 2.38E-02 |
| Eif4h | 1438554_x_at | E05.138.555_10 | 5.01E-11 | 0 | 11.62 | 11.01 | Y | 3.23E-04 |
| Hadhb | 1437172_x_at | E05.023.497_10 | 5.05E-11 | 0 | 9.17 | 8.48 | Y | 1.81E-04 |
| 1600012H06Rik | 1428218_a_at | E17.022.410_10 | 5.31E-11 | 0 | 8.13 | 7.48 | Y | 1.81E-05 |
| Eef1d | 1439439_x_at | E15.087.204_10 | 5.79E-11 | 0 | 9.72 | 8.9 | Y | 6.84E-04 |
| Cr2 | 1425289_a_at | E01.187.751_10 | 6.27E-11 | 0 | 4.3 | 6.05 | Y | 3.06E-03 |
| Dync1li1 | 1424947_at | E09.099.776_10 | 7.57E-11 | 0 | 7.48 | 6.88 | Y | 2.47E-04 |
| Arhgap21 | 1428368_at | E02.019.239_10 | 1.13E-10 | 0 | 5.44 | 6.35 | Y | 2.29E-02 |
| Nme6 | 1448574_at | E09.102.375_10 | 1.27E-10 | 0 | 6.28 | 6.91 | N | 1.15E-04 |
| Mcat | 1452216_at | E15.087.204_10 | 1.36E-10 | 0 | 6.7 | 6.09 | Y | 3.88E-02 |
| Rbx1 | 1416577_a_at | E15.073.732_10 | 1.45E-10 | 0 | 10.92 | 10.35 | Y | 1.89E-04 |
| Helb | 1419234_at | E10.118.344_10 | 1.52E-10 | 0 | 5.85 | 5.13 | Y | 1.56E-02 |
| Vamp3 | 1437708_x_at | E04.156.084_10 | 1.87E-10 | 0 | 8.75 | 9.58 | Y | 9.53E-04 |
| Anxa11 | 1418468_at | E14.019.954_10 | 2.90E-10 | 0 | 6.6 | 7.48 | Y | 3.28E-04 |
| Slc39a1 | 1424424_at | E03.094.477_10 | 3.01E-10 | 0 | 8.49 | 9.06 | Y | 1.97E-02 |
| Gzme | 1421227_at | E14.041.725_10 | 3.17E-10 | 0 | 5.93 | 9.44 | N | 1.87E-03 |
| Ap1s1 | 1416087_at | E05.138.555_10 | 4.08E-10 | 0 | 8.94 | 8.47 | Y | 2.92E-02 |
| Slc2a9 | 1426568_at | E05.032.425_10 | 4.13E-10 | 0 | 4.21 | 5.7 | Y | 1.15E-04 |
| D530033C11Rik | 1435806_at | E15.045.423_10 | 4.37E-10 | 0 | 6.27 | 5.49 | Y | 1.16E-03 |
| Peci | 1431012_a_at | E13.037.637_10 | 4.47E-10 | 0 | 8.79 | 9.49 | Y | 5.32E-05 |
| Gzme | 1450171_x_at | E14.041.725_10 | 5.00E-10 | 0 | 5.61 | 8.66 | N | 2.55E-03 |
| Fastk | 1460635_at | E05.023.497_10 | 5.96E-10 | 0 | 6.75 | 6.19 | Y | 3.44E-07 |
| 2410018G20Rik | 1452591_a_at | E16.018.064_10 | 8.38E-10 | 0 | 7.98 | 8.68 | Y | 2.30E-03 |
| Rpn1 | 1438943_x_at | E06.092.041_10 | 8.57E-10 | 0 | 10.33 | 9.54 | Y | 8.05E-06 |
| Rcor1 | 1437545_at | E12.105.658_10 | 9.22E-10 | 0 | 8 | 7.34 | Y | 1.22E-02 |
| Nhlrc2 | 1429145_at | E19.055.858_10 | 9.22E-10 | 0 | 6.43 | 5.4 | Y | 4.13E-05 |
| Rfc5 | 1452917_at | E05.107.059_10 | 1.10E-09 | 0 | 8.15 | 7.54 | Y | 1.54E-01 |
| Exoc8 | 1436048_at | E08.129.275_10 | 1.22E-09 | 0 | 7.01 | 6.27 | N | 5.88E-03 |
| 1190017O12Rik | 1417402_at | E16.084.417_10 | 1.26E-09 | 0 | 7.92 | 7.45 | N | 1.99E-03 |
| Grsf1 | 1433457_s_at | E05.085.774_10 | 1.30E-09 | 0 | 8.12 | 7.52 | Y | 7.68E-03 |
| Tox4 | 1416574_at | E14.041.725_10 | 1.31E-09 | 0 | 7.53 | 7.08 | Y | 2.52E-04 |
| Tpp2 | 1421893_a_at | E01.042.090_10 | 1.34E-09 | 0 | 7.02 | 6.46 | Y | 6.17E-02 |
| Trim34 | 1424857_a_at | E07.101.309_10 | 1.40E-09 | 0 | 3.6 | 4.51 | N | 1.28E-03 |
| D19Bwg1357e | 1434173_s_at | E19.023.477_10 | 1.49E-09 | 0 | 10.01 | 9.42 | Y | 6.82E-03 |
| Ets1 | 1422027_a_at | E09.034.366_10 | 1.49E-09 | 0 | 6.61 | 7.52 | Y | 3.88E-11 |
| Rpn1 | 1439257_x_at | E06.092.041_10 | 1.52E-09 | 0 | 10.97 | 10.13 | Y | 8.05E-06 |
| Rpn1 | 1456438_x_at | E06.092.041_10 | 1.72E-09 | 0 | 10.88 | 10.08 | Y | 8.05E-06 |
| Rpgrip1 | 1421144_at | E14.041.725_10 | 1.93E-09 | 0 | 6.4 | 5.84 | Y | 3.35E-01 |
| Rhbdd1 | 1423918_at | E01.086.274_10 | 1.96E-09 | 0 | 7.94 | 7.29 | Y | 1.78E-01 |
| Nrd1 | 1449716_s_at | E04.106.454_10 | 2.02E-09 | 0 | 5.48 | 6.35 | Y | 8.05E-06 |
| Ly6e | 1453304_s_at | E15.087.204_10 | 2.14E-09 | 0 | 12.24 | 11.44 | Y | 4.44E-04 |
| Cno | 1459783_s_at | E05.044.682_10 | 2.36E-09 | 0 | 7.86 | 6.99 | Y | 1.28E-04 |
| Hsd17b12 | 1450011_at | E02.092.755_10 | 2.72E-09 | 0 | 10.18 | 9.45 | Y | 1.36E-02 |
| Tspo | 1438948_x_at | E15.087.204_10 | 2.73E-09 | 0 | 9.9 | 9.08 | Y | 2.64E-03 |
| A930016P21Rik | 1428683_at | E15.024.918_10 | 2.90E-09 | 0 | 6.98 | 6.43 | Y | 4.35E-02 |
| Tcf25 | 1451081_a_at | E08.129.275_10 | 3.07E-09 | 0 | 8.2 | 7.55 | Y | 9.83E-03 |
| Pgam5 | 1428080_at | E05.103.695_10 | 4.10E-09 | 0 | 8.29 | 7.75 | Y | 1.62E-02 |
| Lias | 1447027_s_at | E05.044.682_10 | 4.14E-09 | 0 | 9.13 | 8.58 | Y | 9.57E-03 |
| Cnnm3 | 1420482_at | E01.033.082_10 | 4.93E-09 | 0 | 5.49 | 4.95 | Y | 1.07E-01 |
| Mospd3 | 1460452_at | E05.138.555_10 | 5.15E-09 | 0 | 6.18 | 6.7 | Y | 3.99E-03 |
| Mrpl1 | 1450948_a_at | E05.085.774_10 | 5.29E-09 | 0 | 6.51 | 5.69 | Y | 2.17E-02 |
| Jhdm1d | 1456150_at | E06.049.290_10 | 6.39E-09 | 0 | 7.81 | 6.9 | Y | 1.71E-04 |
| Helb | 1419235_s_at | E10.118.344_10 | 6.44E-09 | 0 | 5.9 | 5.28 | Y | 3.23E-02 |
| Trappc5 | 1448999_at | E08.023.645_10 | 6.64E-09 | 0 | 6.62 | 6.09 | Y | 1.53E-01 |
| Ubtf | 1453097_a_at | E11.101.632_10 | 6.78E-09 | 0 | 8.32 | 7.79 | Y | 3.45E-04 |
| Poldip3 | 1434176_x_at | E15.073.732_10 | 7.31E-09 | 0 | 8.96 | 8.54 | Y | 1.43E-02 |
| Xpot | 1441682_s_at | E10.118.344_10 | 7.38E-09 | 0 | 9.46 | 8.86 | Y | 4.95E-02 |
| Tmem63a | 1423871_at | E01.187.751_10 | 7.46E-09 | 0 | 8.57 | 7.84 | Y | 6.96E-02 |
| Cct6a | 1455988_a_at | E05.126.262_10 | 7.46E-09 | 0 | 11.63 | 11.13 | Y | 6.71E-02 |
| Abcb10 | 1416402_at | E08.129.275_10 | 8.08E-09 | 0 | 7.05 | 6.41 | Y | 5.18E-02 |
| Mcm7 | 1416030_a_at | E05.138.555_10 | 8.85E-09 | 0 | 8.82 | 8 | Y | 1.29E-02 |
| Rnf149 | 1429321_at | E01.042.090_10 | 9.03E-09 | 0 | 10.42 | 9.79 | Y | 6.74E-02 |
| Sp2 | 1426237_at | E11.117.260_10 | 9.73E-09 | 0 | 5.14 | 4.61 | Y | 1.22E-03 |
| N6amt2 | 1449072_a_at | E14.051.169_10 | 9.77E-09 | 0 | 7.75 | 7.2 | Y | 5.49E-04 |
| Denr | 1420367_at | E05.126.262_10 | 1.06E-08 | 0 | 4.63 | 3.78 | N | 3.20E-03 |
| Cnnm3 | 1420481_at | E01.060.215_10 | 1.14E-08 | 0 | 6.04 | 5.46 | Y | 2.70E-01 |
| Mettl8 | 1451141_at | E02.078.067_10 | 1.19E-08 | 0 | 6.37 | 5.62 | Y | 1.54E-01 |
| Kcnmb4 | 1449471_at | E10.102.359_10 | 1.27E-08 | 0 | 4.87 | 6.24 | Y | 2.38E-05 |
| Txn2 | 1455640_a_at | E15.087.204_10 | 1.41E-08 | 0 | 8.61 | 8.27 | Y | 8.79E-02 |
| Mfap1a | 1449445_x_at | E02.112.734_10 | 1.41E-08 | 0 | 8.69 | 8.25 | Y | 4.49E-03 |
| D5Wsu178e | 1431802_a_at | E05.023.497_10 | 1.44E-08 | 0 | 8.26 | 7.57 | Y | 1.51E-03 |
| 2210009G21Rik | 1431831_at | E02.112.734_10 | 1.47E-08 | 0 | 5.55 | 5.06 | N | 1.30E-01 |
| Tmem14c | 1416479_a_at | E13.037.637_10 | 1.74E-08 | 0 | 9.06 | 8.64 | Y | 7.69E-05 |
| Tbk1 | 1460315_s_at | E10.118.344_10 | 1.87E-08 | 0 | 9.19 | 8.74 | Y | 1.53E-01 |
| Ttc5 | 1424508_at | E14.051.169_10 | 1.96E-08 | 0 | 7.85 | 7.48 | Y | 1.44E-04 |
| 2610207I05Rik | 1457262_at | E07.107.183_10 | 2.00E-08 | 0.001 | 6.26 | 7.17 | Y | 3.20E-02 |
| Arl8b | 1428137_at | E06.131.131_10 | 2.07E-08 | 0 | 7.69 | 7.01 | Y | 3.22E-02 |
| Pi4k2b | 1420411_a_at | E05.044.682_10 | 2.17E-08 | 0 | 8.05 | 7.08 | Y | 6.46E-04 |
| Ndst1 | 1460436_at | E18.060.777_10 | 2.25E-08 | 0 | 9.47 | 8.77 | Y | 9.10E-04 |
| Tspo | 1456251_x_at | E15.087.204_10 | 2.28E-08 | 0 | 9.85 | 9.07 | Y | 1.84E-02 |
| Ccni | 1416427_at | E05.085.774_10 | 2.29E-08 | 0 | 10.98 | 10.36 | Y | 3.27E-03 |
| Il18 | 1417932_at | E09.034.366_10 | 2.68E-08 | 0 | 7.82 | 8.97 | Y | 1.33E-03 |
| Phf17 | 1426753_at | E03.026.032_10 | 3.04E-08 | 0 | 5.46 | 6.29 | Y | 8.74E-04 |
| Bid | 1417045_at | E06.126.705_10 | 3.53E-08 | 0 | 7.35 | 6.59 | Y | 6.04E-02 |
| 4922501C03Rik | 1443899_at | E09.094.015_10 | 3.57E-08 | 0 | 5.03 | 5.55 | Y | 1.87E-01 |
| Atp5a1 | 1449710_s_at | E18.077.468_10 | 3.66E-08 | 0 | 10.93 | 10.3 | Y | 2.75E-04 |
| Lias | 1418700_at | E05.044.682_10 | 3.96E-08 | 0 | 8.17 | 7.72 | Y | 1.79E-01 |
| Plekhm1 | 1454746_at | E11.101.632_10 | 4.10E-08 | 0 | 6.91 | 6.41 | N | 4.31E-03 |
| 1110019K23Rik | 1429600_at | E05.044.682_10 | 4.10E-08 | 0 | 5.42 | 4.66 | N | 4.05E-02 |
| Polr3a | 1437525_a_at | E14.019.954_10 | 4.13E-08 | 0 | 6.49 | 5.74 | Y | 1.35E-03 |
| Zfp422-rs1 | 1434896_at | E17.022.410_10 | 4.27E-08 | 0.001 | 7.28 | 6.55 | Y | 4.11E-04 |
| 2410002O22Rik | 1418667_at | E13.105.239_10 | 4.28E-08 | 0 | 6.26 | 5.49 | Y | 1.42E-03 |
| Cbr4 | 1451398_at | E08.038.758_10 | 4.44E-08 | 0 | 7.31 | 6.57 | Y | 8.01E-04 |
| Wdr37 | 1434076_at | E13.001.439_10 | 4.53E-08 | 0 | 6.82 | 6.35 | Y | 1.29E-04 |
| Rabl3 | 1418039_at | E16.033.089_10 | 4.65E-08 | 0 | 6.74 | 6.24 | Y | 4.66E-03 |
| Ncoa7 | 1454809_at | E10.023.222_10 | 4.77E-08 | 0 | 5.3 | 6.2 | Y | 1.35E-03 |
| Tcf20 | 1421910_at | E15.087.204_10 | 4.86E-08 | 0 | 7.46 | 6.88 | Y | 1.97E-01 |
| Chchd2 | 1428161_a_at | E05.126.262_10 | 5.04E-08 | 0 | 12.55 | 12.19 | Y | 2.80E-03 |
| Orc5l | 1415830_at | E05.023.497_10 | 5.05E-08 | 0 | 7.56 | 7.06 | Y | 5.78E-04 |
| Foxc2 | 1416693_at | E08.112.069_10 | 5.11E-08 | 0 | 5.81 | 7.28 | N | 1.89E-01 |
| Bag4 | 1449186_at | E08.023.645_10 | 5.24E-08 | 0 | 6.73 | 6.1 | Y | 3.83E-04 |
| Prpf40a | 1450035_a_at | E02.056.057_10 | 5.27E-08 | 0 | 7.86 | 7 | Y | 3.40E-04 |
| Pgm1 | 1453283_at | E05.044.682_10 | 5.59E-08 | 0 | 8.33 | 8.95 | Y | 5.01E-02 |
| Dnlz | 1428913_at | E02.019.239_10 | 5.84E-08 | 0 | 7.21 | 6.74 | Y | 1.74E-03 |
| Rpl7l1 | 1439780_at | E17.068.056_10 | 6.05E-08 | 0 | 6.29 | 5.55 | Y | 1.34E-03 |
| Nfatc3 | 1452497_a_at | E08.109.032_10 | 6.29E-08 | 0 | 6.06 | 6.54 | Y | 3.17E-01 |
| Whsc1l1 | 1457793_a_at | E08.048.274_10 | 6.38E-08 | 0 | 5.29 | 5.99 | Y | 7.57E-02 |
| Eif2s1 | 1420491_at | E12.080.625_10 | 6.47E-08 | 0 | 5.55 | 7.03 | Y | 1.31E-03 |
| Tagap | 1460453_at | E17.022.410_10 | 6.69E-08 | 0 | 6.96 | 6.33 | Y | 7.97E-05 |
| Cep164 | 1435676_at | E09.034.366_10 | 6.73E-08 | 0 | 7.47 | 6.91 | Y | 4.03E-03 |
| Hax1 | 1438180_x_at | E03.081.606_10 | 7.00E-08 | 0 | 10.94 | 10.29 | Y | 2.85E-05 |
| Bxdc5 | 1459573_at | E03.147.739_10 | 7.54E-08 | 0 | 5.86 | 5.31 | Y | 6.39E-05 |
| Spry2 | 1421656_at | E14.094.324_10 | 7.64E-08 | 0 | 3.95 | 4.66 | N | 3.29E-01 |
| A2m | 1434719_at | E06.126.705_10 | 7.94E-08 | 0 | 3.88 | 5.53 | Y | 1.36E-02 |
| Trappc4 | 1415674_a_at | E09.034.366_10 | 8.69E-08 | 0 | 8.63 | 8.21 | Y | 5.14E-02 |
| Cflar | 1425686_at | E01.033.082_10 | 8.73E-08 | 0 | 6.85 | 6.07 | N | 1.41E-02 |
| H13 | 1438456_at | E02.157.973_10 | 8.79E-08 | 0 | 6.02 | 5.15 | Y | 1.39E-02 |
| Jmy | 1420639_at | E13.102.230_10 | 9.46E-08 | 0 | 6.02 | 6.76 | Y | 8.09E-04 |
| Tuba1b | 1423846_x_at | E15.101.153_10 | 9.62E-08 | 0 | 13.2 | 12.79 | Y | 4.28E-03 |
| Arhgap1 | 1451309_at | E02.112.734_10 | 9.72E-08 | 0 | 7.9 | 7.41 | Y | 1.84E-02 |
| D2hgdh | 1437243_at | E01.083.652_10 | 9.97E-08 | 0 | 5.42 | 5.86 | Y | 4.99E-03 |
| Cpsf6 | 1428233_at | E10.118.344_10 | 1.04E-07 | 0 | 7.61 | 6.93 | Y | 1.40E-01 |
| Rnd3 | 1416701_at | E02.112.734_10 | 1.06E-07 | 0 | 8.78 | 8.02 | N | 3.37E-01 |
| Tle3 | 1449554_at | E09.052.894_10 | 1.09E-07 | 0 | 6.33 | 5.79 | Y | 3.56E-02 |
| Commd3 | 1433539_at | E02.019.239_10 | 1.15E-07 | 0 | 9.92 | 9.43 | Y | 1.35E-01 |
| Endod1 | 1426543_x_at | E09.034.366_10 | 1.18E-07 | 0 | 7.54 | 6.65 | Y | 1.49E-02 |
| Rnd3 | 1416700_at | E02.056.057_10 | 1.18E-07 | 0 | 8.74 | 8.14 | Y | 6.30E-02 |
| Stch | 1453172_at | E16.084.417_10 | 1.19E-07 | 0 | 7.61 | 8.51 | Y | 6.63E-04 |
| Glrx2 | 1437679_a_at | E01.144.066_10 | 1.21E-07 | 0 | 7.99 | 7.29 | Y | 9.35E-04 |
| 2810457I06Rik | 1436805_at | E09.034.366_10 | 1.21E-07 | 0 | 5.39 | 6.18 | N | 3.64E-04 |
| 1700029I01Rik | 1436574_at | E07.002.028_10 | 1.25E-07 | 0.001 | 5.16 | 4.01 | Y | 6.78E-03 |
| Ddhd1 | 1455321_at | E14.041.725_10 | 1.27E-07 | 0 | 5.24 | 6.07 | Y | 9.77E-05 |
| Atad1 | 1448763_at | E19.023.477_10 | 1.29E-07 | 0 | 8.22 | 7.79 | Y | 3.79E-02 |
| Snapc3 | 1428760_at | E04.080.606_10 | 1.31E-07 | 0 | 6.76 | 7.33 | Y | 2.82E-04 |
| Naprt1 | 1454748_at | E15.087.204_10 | 1.33E-07 | 0 | 5.81 | 6.8 | Y | 1.76E-03 |
| Nap1l1 | 1420478_at | E10.118.344_10 | 1.33E-07 | 0 | 10.73 | 10.28 | Y | 2.90E-01 |
| Ogg1 | 1430078_a_at | E06.104.373_10 | 1.38E-07 | 0 | 6.13 | 6.89 | Y | 2.85E-02 |
| Gimap8 | 1456061_at | E06.032.288_10 | 1.42E-07 | 0 | 6.92 | 5.64 | Y | 2.06E-01 |
| BC003266 | 1449189_at | E04.121.681_10 | 1.42E-07 | 0 | 8.03 | 7.65 | N | 3.38E-01 |
| Pa2g4 | 1420142_s_at | E10.118.344_10 | 1.47E-07 | 0 | 8.16 | 7.29 | Y | 9.92E-02 |
| Lpp | 1455314_at | E16.033.089_10 | 1.47E-07 | 0 | 9.93 | 9.25 | Y | 1.86E-01 |
| Vnn3 | 1420723_at | E10.023.222_10 | 1.55E-07 | 0 | 3.93 | 4.75 | N | 1.66E-03 |
| Pitpnb | 1434257_s_at | E05.044.682_10 | 1.59E-07 | 0 | 9.14 | 8.82 | Y | 2.50E-01 |
| Anapc4 | 1423930_at | E05.044.682_10 | 1.60E-07 | 0 | 6.93 | 6.35 | Y | 3.78E-04 |
| Ppcdc | 1424335_at | E09.052.894_10 | 1.60E-07 | 0 | 6.61 | 6.14 | Y | 3.45E-04 |
| Cox19 | 1434923_at | E05.138.555_10 | 1.64E-07 | 0 | 8.39 | 8 | Y | 2.44E-02 |
| 2610524H06Rik | 1429244_at | E05.107.059_10 | 1.71E-07 | 0 | 7.93 | 7 | Y | 1.22E-03 |
| Nrp | 1440263_at | E12.090.698_10 | 1.81E-07 | 0 | 6.08 | 5.58 | Y | 1.35E-03 |
| Gmfb | 1448570_at | E14.051.169_10 | 1.84E-07 | 0 | 9.4 | 8.98 | Y | 1.82E-02 |
| 6330577E15Rik | 1425194_a_at | E19.055.858_10 | 1.86E-07 | 0.001 | 10.73 | 10.18 | Y | 4.76E-04 |
| Efhd2 | 1437478_s_at | E04.141.582_10 | 1.87E-07 | 0 | 11.04 | 10.48 | Y | 1.82E-02 |
| Tmem181 | 1435948_at | E17.022.410_10 | 1.90E-07 | 0 | 8.02 | 7.35 | Y | 5.32E-05 |
| Aurkaip1 | 1416245_at | E04.156.084_10 | 1.93E-07 | 0 | 8.67 | 9.1 | Y | 2.02E-04 |
| Bfar | 1426489_s_at | E16.018.064_10 | 1.96E-07 | 0 | 8.29 | 7.68 | Y | 8.03E-04 |
| Tpp2 | 1430575_a_at | E01.033.082_10 | 1.98E-07 | 0 | 6.1 | 5.38 | Y | 6.44E-02 |
| 0610009K11Rik | 1451401_a_at | E04.141.582_10 | 1.98E-07 | 0 | 6.06 | 5.57 | Y | 3.20E-02 |
| A630033E08Rik | 1437128_a_at | E17.022.410_10 | 2.03E-07 | 0 | 6.07 | 5.3 | Y | 2.28E-04 |
| 9230105E10Rik | 1443858_at | E07.101.309_10 | 2.04E-07 | 0 | 6.96 | 6.18 | Y | 5.46E-02 |
| Nif3l1 | 1438728_at | E01.060.215_10 | 2.11E-07 | 0 | 5.31 | 4.72 | Y | 3.88E-02 |
| Smarcd2 | 1448401_at | E11.101.632_10 | 2.11E-07 | 0 | 7.92 | 7.59 | Y | 3.38E-04 |
| Zfr | 1449552_at | E15.013.193_10 | 2.11E-07 | 0 | 7.54 | 7.98 | Y | 3.71E-03 |
| Gnpnat1 | 1423156_at | E14.023.871_10 | 2.14E-07 | 0 | 7.99 | 7.43 | Y | 1.23E-02 |
| 1600012H06Rik | 1428217_at | E17.022.410_10 | 2.21E-07 | 0 | 7.36 | 6.77 | Y | 1.26E-05 |
| Nup155 | 1449200_at | E15.024.918_10 | 2.21E-07 | 0 | 7.04 | 6.6 | Y | 2.63E-01 |
| Wdr48 | 1452162_at | E09.116.239_10 | 2.39E-07 | 0 | 7.16 | 7.75 | Y | 6.01E-04 |
| Afg3l1 | 1449045_at | E08.129.275_10 | 2.39E-07 | 0 | 7.48 | 7.09 | Y | 1.19E-01 |
| 1200013P24Rik | 1415721_a_at | E16.018.064_10 | 2.42E-07 | 0 | 7.24 | 6.93 | Y | 4.73E-03 |
| Mnda | 1452349_x_at | E01.169.742_10 | 2.44E-07 | 0.001 | 8.59 | 9.78 | N | 1.06E-01 |
| Polr2k | 1452596_at | E15.024.918_10 | 2.45E-07 | 0 | 9.89 | 9.43 | Y | 1.68E-02 |
| Nhlrc2 | 1453048_at | E19.055.858_10 | 2.45E-07 | 0 | 6.88 | 6.41 | Y | 8.33E-05 |
| Ifngr1 | 1448167_at | E10.009.586_10 | 2.46E-07 | 0 | 8.77 | 8.3 | Y | 1.82E-02 |
| Ugt8a | 1419063_at | E03.127.496_10 | 2.49E-07 | 0 | 3.19 | 5.35 | Y | 5.42E-04 |
| Sgol2 | 1437370_at | E01.042.090_10 | 2.53E-07 | 0 | 7.57 | 6.7 | Y | 4.25E-02 |
| OTTMUSG00000010657 | 1424784_at | E07.002.028_10 | 2.66E-07 | 0.001 | 4.82 | 3.59 | Y | 1.22E-02 |
| Mcph1 | 1429911_at | E08.020.455_10 | 2.70E-07 | 0 | 6.16 | 5.58 | N | 4.99E-03 |
| Rpl7l1 | 1452049_at | E17.056.536_10 | 2.81E-07 | 0 | 8.23 | 7.72 | Y | 3.12E-03 |
| Pgd | 1423706_a_at | E04.141.582_10 | 2.88E-07 | 0 | 9.57 | 8.92 | Y | 3.09E-03 |
| Fam73a | 1437002_at | E03.158.424_10 | 2.93E-07 | 0 | 4.32 | 5.22 | N | 5.83E-02 |
| Shoc2 | 1423129_at | E19.055.858_10 | 2.98E-07 | 0 | 9.16 | 8.75 | Y | 2.50E-04 |
| Vhlh | 1434708_at | E06.139.952_10 | 3.05E-07 | 0 | 6.53 | 6.1 | Y | 5.92E-03 |
| Myo1b | 1448989_a_at | E01.042.090_10 | 3.11E-07 | 0 | 8.69 | 7.95 | Y | 2.68E-01 |
| Uba3 | 1417202_s_at | E06.126.705_10 | 3.16E-07 | 0 | 9.02 | 8.29 | Y | 5.20E-02 |
| Rbm34 | 1429587_at | E08.129.275_10 | 3.20E-07 | 0 | 7.84 | 7.35 | Y | 2.23E-01 |
| Sav1 | 1448204_at | E12.029.924_10 | 3.22E-07 | 0 | 9.53 | 8.98 | Y | 2.42E-01 |
| Tmod1 | 1422754_at | E04.043.559_10 | 3.23E-07 | 0 | 4.88 | 5.42 | N | 4.18E-03 |
| Copb2 | 1456175_a_at | E09.099.776_10 | 3.23E-07 | 0 | 9.52 | 9.14 | Y | 2.30E-03 |
| Rab24 | 1421872_at | E13.053.052_10 | 3.41E-07 | 0 | 8.92 | 8.14 | N | 1.79E-03 |
| Zkscan1 | 1429152_at | E05.138.555_10 | 3.43E-07 | 0 | 6.84 | 6.41 | Y | 3.18E-03 |
| Sod2 | 1448610_a_at | E17.022.410_10 | 3.49E-07 | 0 | 9.25 | 8.55 | Y | 1.57E-04 |
| Tbc1d23 | 1426899_at | E16.047.738_10 | 3.65E-07 | 0 | 7.21 | 6.75 | Y | 4.90E-05 |
| Ppp1r2 | 1448684_at | E16.033.089_10 | 3.68E-07 | 0 | 8.77 | 8.39 | N | 6.99E-03 |
| Eif3b | 1426674_at | E05.138.555_10 | 3.70E-07 | 0 | 9.74 | 9.37 | Y | 2.44E-02 |
| Gsr | 1421817_at | E08.023.645_10 | 3.73E-07 | 0 | 9.81 | 9.07 | N | 6.68E-04 |
| Clec4g | 1427428_at | E08.023.645_10 | 3.74E-07 | 0 | 3.85 | 4.46 | Y | 1.77E-03 |
| Wwc2 | 1448611_at | E08.038.758_10 | 3.78E-07 | 0 | 7.35 | 6.69 | Y | 2.25E-02 |
| Eif2ak2 | 1440866_at | E17.068.056_10 | 3.81E-07 | 0 | 4.92 | 4.12 | N | 7.21E-03 |
| Tgds | 1424526_a_at | E14.111.590_10 | 3.82E-07 | 0 | 7.5 | 6.91 | Y | 5.74E-03 |
| Rgs5 | 1420940_x_at | E01.187.751_10 | 3.87E-07 | 0 | 5.12 | 6.18 | Y | 2.67E-01 |
| Myl6 | 1434396_a_at | E10.118.344_10 | 3.94E-07 | 0 | 12.37 | 12.03 | Y | 1.14E-01 |
| Mvd | 1417303_at | E08.129.275_10 | 3.95E-07 | 0 | 6.37 | 5.38 | Y | 3.89E-02 |
| Sec22c | 1456091_at | E09.116.239_10 | 4.28E-07 | 0 | 4.52 | 4.04 | Y | 2.87E-02 |
| Bdh2 | 1453011_at | E03.140.010_10 | 4.33E-07 | 0 | 3.87 | 4.58 | Y | 3.63E-03 |
| Rint1 | 1456394_at | E05.023.497_10 | 4.40E-07 | 0 | 3.86 | 4.26 | Y | 9.19E-02 |
| 2310061F22Rik | 1428350_at | E08.129.275_10 | 4.53E-07 | 0 | 6.35 | 6.02 | Y | 3.39E-01 |
| Mta2 | 1423165_a_at | E19.005.316_10 | 4.55E-07 | 0.001 | 7.97 | 7.63 | Y | 2.66E-02 |
| Sf3b2 | 1438637_x_at | E19.005.316_10 | 4.57E-07 | 0 | 8.62 | 7.92 | Y | 8.05E-06 |
| Mpst | 1418356_at | E15.087.204_10 | 4.65E-07 | 0 | 5.95 | 5.38 | Y | 1.48E-02 |
| BC012278 | 1425297_at | E13.056.787_10 | 4.73E-07 | 0 | 6.42 | 6.01 | Y | 1.89E-04 |
| Rad23a | 1422964_at | E08.088.925_10 | 4.74E-07 | 0.001 | 6.71 | 7.24 | Y | 3.58E-02 |
| Ttc30b | 1423672_at | E02.078.067_10 | 4.74E-07 | 0 | 6.93 | 6.1 | Y | 5.74E-03 |
| 6030443O07Rik | 1438233_at | E19.055.858_10 | 4.76E-07 | 0 | 7.54 | 6.92 | Y | 2.93E-03 |
| Pi4k2b | 1416489_at | E05.044.682_10 | 4.88E-07 | 0 | 8.38 | 7.8 | Y | 1.02E-03 |
| Tmem134 | 1421993_a_at | E19.005.316_10 | 4.90E-07 | 0 | 6.47 | 5.96 | Y | 2.08E-02 |
| Endod1 | 1426541_a_at | E09.034.366_10 | 5.00E-07 | 0 | 8.11 | 7.14 | Y | 1.12E-02 |
| Ipo9 | 1441645_s_at | E01.135.010_10 | 5.05E-07 | 0 | 8.43 | 7.84 | Y | 2.14E-02 |
| Ppp1r2 | 1417342_at | E16.033.089_10 | 5.11E-07 | 0 | 5.24 | 5.9 | Y | 2.85E-03 |
| Ktelc1 | 1426535_at | E16.033.089_10 | 5.12E-07 | 0 | 8.12 | 7.74 | Y | 2.53E-02 |
| C130032J12Rik | 1433879_a_at | E14.041.725_10 | 5.17E-07 | 0 | 8.5 | 8.02 | Y | 1.10E-02 |
| Krr1 | 1420139_s_at | E10.118.344_10 | 5.23E-07 | 0 | 8.75 | 8.18 | Y | 1.19E-01 |
| Rad17 | 1448762_at | E13.102.230_10 | 5.49E-07 | 0 | 7.45 | 6.99 | Y | 2.64E-03 |
| Eif4b | 1426378_at | E15.101.379_10 | 5.60E-07 | 0 | 10.22 | 9.77 | Y | 3.34E-01 |
| Nadk | 1416248_at | E04.156.084_10 | 5.65E-07 | 0 | 9.56 | 9.16 | Y | 1.28E-03 |
| Zfp617 | 1449546_a_at | E08.054.070_10 | 5.66E-07 | 0 | 6.91 | 6.42 | Y | 1.52E-04 |
| Fvt1 | 1440331_at | E01.094.380_10 | 5.73E-07 | 0 | 6.02 | 5.54 | Y | 6.49E-02 |
| Atpbd4 | 1426548_a_at | E02.112.734_10 | 5.77E-07 | 0 | 7.6 | 7.08 | N | 2.72E-01 |
| Serpinb9 | 1439790_at | E13.037.637_10 | 5.86E-07 | 0 | 5.57 | 7.34 | N | 5.49E-04 |
| Tacc3 | 1417450_a_at | E05.044.682_10 | 5.91E-07 | 0 | 7.52 | 6.87 | N | 1.46E-02 |
| Arpc5 | 1448129_at | E01.144.066_10 | 6.11E-07 | 0 | 11.09 | 10.7 | N | 1.53E-01 |
| Eif2ak2 | 1422005_at | E17.087.010_10 | 6.11E-07 | 0 | 5.94 | 5.27 | N | 1.12E-01 |
| Cwf19l1 | 1434065_at | E19.055.858_10 | 6.26E-07 | 0 | 5.87 | 5.46 | Y | 2.80E-04 |
| Nod1 | 1454733_at | E06.032.288_10 | 6.35E-07 | 0 | 6.52 | 5.91 | Y | 1.60E-01 |
| H2-Ea | 1422891_at | E17.022.410_10 | 6.40E-07 | 0 | 7.51 | 8.78 | N | 1.29E-03 |
| Zdhhc24 | 1428278_at | E19.005.316_10 | 6.53E-07 | 0 | 5.22 | 4.64 | Y | 4.37E-04 |
| Ripk1 | 1449485_at | E13.014.119_10 | 6.64E-07 | 0 | 8.56 | 8.27 | Y | 4.25E-03 |
| Nars | 1452866_at | E18.056.581_10 | 6.69E-07 | 0 | 10.62 | 10.17 | Y | 2.50E-04 |
| Cad | 1452829_at | E05.044.682_10 | 6.70E-07 | 0 | 7.81 | 7.14 | Y | 6.63E-04 |
| Slc2a3 | 1437052_s_at | E06.147.529_10 | 6.74E-07 | 0 | 7.25 | 8.58 | Y | 7.30E-02 |
| D130059P03Rik | 1436632_at | E06.032.288_10 | 6.94E-07 | 0 | 6.88 | 6.25 | Y | 7.64E-04 |
| Aktip | 1423364_a_at | E08.109.032_10 | 7.02E-07 | 0 | 7.17 | 6.39 | Y | 1.20E-02 |
| Pten | 1455728_at | E19.055.858_10 | 7.06E-07 | 0 | 7.65 | 7.09 | Y | 1.41E-01 |
| Ube3b | 1452149_at | E05.107.059_10 | 7.17E-07 | 0 | 7.85 | 7.52 | Y | 1.95E-02 |
| Ankrd13c | 1435868_at | E03.140.010_10 | 7.20E-07 | 0 | 6.66 | 7.13 | Y | 2.25E-01 |
| Frg1 | 1417253_at | E08.054.070_10 | 7.27E-07 | 0 | 8.72 | 8.23 | Y | 8.05E-06 |
| Hdgf | 1419964_s_at | E03.081.606_10 | 7.28E-07 | 0 | 9.91 | 9.41 | Y | 1.85E-04 |
| Snw1 | 1429002_at | E12.090.698_10 | 7.38E-07 | 0 | 8.6 | 8.21 | Y | 6.78E-03 |
| Chuk | 1417091_at | E19.055.858_10 | 7.50E-07 | 0 | 8.16 | 7.69 | Y | 4.42E-03 |
| Pkm2 | 1417308_at | E09.034.366_10 | 7.76E-07 | 0 | 12.3 | 11.97 | N | 1.46E-02 |
| Lysmd3 | 1460335_at | E13.102.230_10 | 7.91E-07 | 0 | 8.11 | 7.52 | Y | 8.06E-04 |
| AA881470 | 1452390_at | E05.138.555_10 | 7.96E-07 | 0 | 5.13 | 4.82 | Y | 6.27E-04 |
| Ppm1a | 1415678_at | E12.070.033_10 | 7.97E-07 | 0 | 9.36 | 9.03 | Y | 2.03E-02 |
| Smarcd2 | 1448400_a_at | E11.101.632_10 | 8.47E-07 | 0 | 9.29 | 8.67 | Y | 1.63E-03 |
| Angel2 | 1448360_s_at | E01.187.751_10 | 8.51E-07 | 0.001 | 8.03 | 7.49 | Y | 2.59E-02 |
| Pfkfb3 | 1416432_at | E02.005.251_10 | 8.59E-07 | 0 | 7.07 | 6.2 | Y | 4.49E-03 |
| Dynll1 | 1440278_at | E05.107.059_10 | 8.63E-07 | 0.001 | 4.81 | 4.18 | Y | 3.74E-03 |
| Sypl | 1422880_at | E12.053.286_10 | 8.64E-07 | 0 | 10.15 | 9.67 | Y | 5.40E-02 |
| 2010301N04Rik | 1430297_a_at | E06.126.705_10 | 9.08E-07 | 0 | 5.72 | 5.16 | Y | 2.79E-01 |
| Aldh1a1 | 1416468_at | E19.023.477_10 | 9.36E-07 | 0.001 | 4.45 | 6.51 | N | 3.46E-02 |
| Zfp120 | 1421519_a_at | E02.137.900_10 | 9.44E-07 | 0 | 5.82 | 5.13 | Y | 2.15E-02 |
| Txn2 | 1452782_a_at | E15.087.204_10 | 9.44E-07 | 0.002 | 8.34 | 8.02 | N | 2.18E-01 |
| Rnasen | 1438505_s_at | E15.024.918_10 | 9.44E-07 | 0 | 8.21 | 7.88 | Y | 1.43E-01 |
| Reep3 | 1424781_at | E10.102.359_10 | 9.46E-07 | 0 | 8.49 | 7.88 | Y | 2.35E-04 |
| Tmod3 | 1423089_at | E09.052.894_10 | 9.65E-07 | 0 | 8.77 | 7.8 | Y | 3.11E-02 |
| Paics | 1423564_a_at | E05.085.774_10 | 9.84E-07 | 0 | 10.25 | 9.69 | Y | 1.98E-03 |
| Rfc4 | 1438161_s_at | E16.018.064_10 | 9.87E-07 | 0 | 6.73 | 5.63 | Y | 2.20E-02 |
| Rpp14 | 1419460_at | E14.019.954_10 | 1.04E-06 | 0 | 6.35 | 5.8 | Y | 3.01E-04 |
| Rsad1 | 1437449_at | E11.101.632_10 | 1.04E-06 | 0 | 5.86 | 5.12 | Y | 9.53E-04 |
| 1300018I05Rik | 1423681_at | E17.022.410_10 | 1.06E-06 | 0.001 | 7.21 | 6.73 | Y | 6.63E-04 |
| Tm2d2 | 1456663_x_at | E08.023.645_10 | 1.06E-06 | 0 | 6.62 | 7.25 | Y | 5.88E-05 |
| Sfrs2ip | 1460445_at | E15.101.379_10 | 1.07E-06 | 0 | 7.25 | 6.78 | Y | 3.79E-02 |
| M6prbp1 | 1416424_at | E17.068.056_10 | 1.08E-06 | 0.001 | 7.81 | 7.42 | Y | 7.05E-04 |
| Wdr37 | 1454823_at | E06.001.969_10 | 1.11E-06 | 0 | 6.26 | 5.84 | Y | 2.73E-01 |
| Lars2 | 1435682_at | E09.116.239_10 | 1.15E-06 | 0 | 6.88 | 6.47 | Y | 3.38E-04 |
| Ola1 | 1430053_a_at | E02.112.734_10 | 1.15E-06 | 0.001 | 8.8 | 7.99 | Y | 2.42E-01 |
| Hibadh | 1435967_s_at | E06.046.553_10 | 1.18E-06 | 0 | 9.04 | 8.6 | Y | 8.76E-03 |
| Ppox | 1416618_at | E01.187.751_10 | 1.19E-06 | 0 | 6.22 | 5.88 | N | 2.91E-01 |
| 2510002D24Rik | 1457675_at | E16.018.064_10 | 1.21E-06 | 0 | 5.58 | 5.06 | Y | 2.26E-01 |
| Polr2b | 1433552_a_at | E05.044.682_10 | 1.23E-06 | 0 | 9.52 | 9.19 | Y | 9.29E-02 |
| Slu7 | 1425489_at | E11.028.442_10 | 1.26E-06 | 0 | 5.03 | 4.66 | Y | 3.66E-02 |
| Zfp263 | 1453105_at | E16.018.064_10 | 1.26E-06 | 0 | 6.27 | 5.47 | Y | 1.86E-02 |
| Zfp68 | 1448760_at | E05.138.555_10 | 1.28E-06 | 0 | 7.02 | 6.46 | Y | 3.84E-02 |
| Trio | 1433745_at | E15.024.918_10 | 1.29E-06 | 0 | 8.26 | 7.53 | Y | 1.09E-02 |
| Bat5 | 1460709_a_at | E17.022.410_10 | 1.30E-06 | 0 | 7.77 | 7.33 | Y | 2.80E-04 |
| Poldip3 | 1437335_x_at | E15.073.732_10 | 1.32E-06 | 0.001 | 8.12 | 7.79 | Y | 2.69E-02 |
| 2210012G02Rik | 1418662_at | E04.106.454_10 | 1.32E-06 | 0 | 4.79 | 5.39 | Y | 1.84E-03 |
| Ncaph2 | 1423699_at | E15.101.153_10 | 1.34E-06 | 0 | 5.59 | 5.09 | Y | 3.76E-02 |
| Ube2j1 | 1417723_at | E04.027.855_10 | 1.35E-06 | 0 | 8.66 | 8.23 | Y | 5.44E-04 |
| Ndufb5 | 1417102_a_at | E03.026.032_10 | 1.36E-06 | 0 | 10.17 | 9.7 | Y | 1.42E-02 |
| Selk | 1423225_at | E14.019.954_10 | 1.37E-06 | 0 | 9.36 | 8.95 | Y | 2.33E-03 |
| Fbxl14 | 1417407_at | E06.126.705_10 | 1.37E-06 | 0 | 7.44 | 6.91 | Y | 1.91E-02 |
| Tmem186 | 1418041_at | E16.018.064_10 | 1.39E-06 | 0 | 4.64 | 4.17 | Y | 5.17E-03 |
| 1110034A24Rik | 1453183_at | E12.070.033_10 | 1.39E-06 | 0 | 6.65 | 6.15 | Y | 2.66E-01 |
| Ttll1 | 1436833_x_at | E15.087.204_10 | 1.43E-06 | 0 | 5.72 | 4.84 | Y | 1.37E-02 |
| Aqr | 1433497_at | E02.112.734_10 | 1.45E-06 | 0 | 7.05 | 6.75 | Y | 2.86E-01 |
| Ddx6 | 1439122_at | E09.034.366_10 | 1.49E-06 | 0.001 | 6.65 | 6.15 | Y | 9.88E-02 |
| 2810407C02Rik | 1452167_at | E03.066.854_10 | 1.52E-06 | 0 | 10.41 | 10.12 | N | 1.29E-04 |
| 2410187C16Rik | 1434144_s_at | E15.042.560_10 | 1.52E-06 | 0 | 6.37 | 5.81 | Y | 5.13E-02 |
| Txnl4a | 1433698_a_at | E18.076.137_10 | 1.52E-06 | 0 | 9.43 | 8.99 | Y | 2.95E-03 |
| Brp16 | 1456738_s_at | E15.057.403_10 | 1.52E-06 | 0.001 | 7.98 | 7.5 | Y | 8.24E-02 |
| Lig3 | 1437198_at | E11.089.228_10 | 1.53E-06 | 0 | 6.12 | 5.54 | Y | 5.77E-04 |
| Pus3 | 1418491_a_at | E09.034.366_10 | 1.55E-06 | 0.001 | 5.55 | 6.09 | N | 2.99E-04 |
| Api5 | 1415813_at | E02.092.755_10 | 1.59E-06 | 0 | 9.42 | 9.14 | Y | 1.84E-01 |
| Zadh1 | 1453156_s_at | E12.080.625_10 | 1.63E-06 | 0 | 6.48 | 5.73 | Y | 7.38E-03 |
| 2010111I01Rik | 1435089_at | E13.056.787_10 | 1.66E-06 | 0 | 7.13 | 6.43 | Y | 2.38E-05 |
| Rnf170 | 1434956_at | E08.038.758_10 | 1.67E-06 | 0 | 6.53 | 5.9 | Y | 6.44E-03 |
| Prpf18 | 1429614_at | E02.005.251_10 | 1.67E-06 | 0 | 7.35 | 6.97 | Y | 8.09E-04 |
| Prkar2a | 1428783_at | E09.099.776_10 | 1.76E-06 | 0 | 8.15 | 7.73 | Y | 4.29E-03 |
| Zmym5 | 1442007_at | E14.051.169_10 | 1.77E-06 | 0 | 5.36 | 4.65 | Y | 1.39E-04 |
| Psme4 | 1426823_s_at | E11.028.442_10 | 1.81E-06 | 0 | 9.31 | 9.61 | N | 5.60E-02 |
| Zfp148 | 1449069_at | E16.033.089_10 | 1.89E-06 | 0 | 8.18 | 7.82 | Y | 1.84E-02 |
| Ranbp1 | 1422547_at | E05.044.682_10 | 1.93E-06 | 0 | 11.48 | 11.16 | N | 3.04E-01 |
| Ttc4 | 1451193_x_at | E04.106.454_10 | 1.96E-06 | 0 | 7.08 | 6.59 | Y | 2.23E-02 |
| 5730453I16Rik | 1415730_at | E19.005.316_10 | 2.01E-06 | 0 | 8.07 | 7.66 | Y | 5.32E-05 |
| Tor1aip2 | 1435526_at | E01.144.066_10 | 2.06E-06 | 0 | 8.51 | 7.82 | Y | 1.49E-02 |
| E130120F12Rik | 1439176_at | E13.056.787_10 | 2.07E-06 | 0 | 5.4 | 4.93 | N | 4.93E-04 |
| Gna13 | 1422555_s_at | E11.101.632_10 | 2.10E-06 | 0 | 8.41 | 7.94 | Y | 6.63E-04 |
| Hook3 | 1446737_a_at | E08.038.758_10 | 2.13E-06 | 0 | 6.31 | 6.91 | N | 3.04E-01 |
| Wdr20a | 1451220_at | E12.105.658_10 | 2.13E-06 | 0.001 | 7.27 | 6.82 | Y | 3.22E-02 |
| Stat4 | 1448713_at | E01.042.090_10 | 2.18E-06 | 0.002 | 4.73 | 5.63 | N | 1.97E-02 |
| Sept11 | 1429233_at | E05.085.774_10 | 2.22E-06 | 0 | 8.29 | 7.77 | Y | 3.78E-03 |
| Reep3 | 1424780_a_at | E10.102.359_10 | 2.27E-06 | 0 | 9.18 | 8.5 | Y | 1.71E-04 |
| Ndst2 | 1417931_at | E14.019.954_10 | 2.28E-06 | 0 | 6.54 | 6.15 | N | 5.61E-04 |
| Nup54 | 1433580_at | E05.085.774_10 | 2.29E-06 | 0.001 | 7.71 | 7.37 | Y | 8.60E-02 |
| Pcgf6 | 1424081_at | E19.055.858_10 | 2.30E-06 | 0 | 7.62 | 7.01 | Y | 6.19E-03 |
| Canx | 1422845_at | E11.052.938_10 | 2.32E-06 | 0 | 9.49 | 8.7 | Y | 3.20E-02 |
| Wbp4 | 1419375_at | E14.093.815_10 | 2.34E-06 | 0.001 | 7.18 | 6.8 | Y | 1.43E-01 |
| Mbp | 1425263_a_at | E18.077.468_10 | 2.40E-06 | 0.001 | 5.43 | 6.46 | Y | 4.99E-03 |
| Anxa7 | 1416137_at | E14.019.954_10 | 2.40E-06 | 0 | 7.02 | 6.56 | Y | 1.46E-04 |
| Smek1 | 1435742_at | E12.105.658_10 | 2.41E-06 | 0 | 7.54 | 7.19 | Y | 2.17E-01 |
| Podxl | 1448688_at | E06.032.288_10 | 2.43E-06 | 0 | 7.08 | 6.46 | N | 1.07E-01 |
| Klhl28 | 1428721_at | E12.061.769_10 | 2.43E-06 | 0 | 7.23 | 6.74 | Y | 7.40E-04 |
| Glrx2 | 1422998_a_at | E01.144.066_10 | 2.45E-06 | 0 | 8.19 | 7.67 | Y | 1.01E-02 |
| Kbtbd7 | 1428192_at | E14.093.815_10 | 2.46E-06 | 0 | 3.97 | 3.4 | Y | 6.09E-02 |
| Ipo9 | 1424466_at | E01.135.010_10 | 2.46E-06 | 0.001 | 7.6 | 6.92 | Y | 3.12E-03 |
| Gpr123 | 1436529_at | E07.136.725_10 | 2.46E-06 | 0 | 3.96 | 5 | N | 1.09E-04 |
| Il1rn | 1425663_at | E02.019.239_10 | 2.49E-06 | 0 | 7.8 | 6.73 | Y | 1.01E-01 |
| Atp2a2 | 1416551_at | E06.032.288_10 | 2.54E-06 | 0.001 | 8.7 | 8.23 | N | 2.79E-01 |
| Dsel | 1438407_at | E09.052.894_10 | 2.57E-06 | 0.001 | 7.89 | 8.36 | Y | 3.31E-01 |
| C330027C09Rik | 1449699_s_at | E16.033.089_10 | 2.59E-06 | 0 | 6.84 | 6.11 | Y | 1.38E-02 |
| Gcap14 | 1452223_s_at | E14.019.954_10 | 2.62E-06 | 0.001 | 7.21 | 6.61 | Y | 5.43E-04 |
| Ttf2 | 1428522_at | E03.108.765_10 | 2.66E-06 | 0 | 6.61 | 6.19 | Y | 3.98E-02 |
| Endod1 | 1426542_at | E09.034.366_10 | 2.67E-06 | 0 | 6.41 | 5.66 | Y | 3.14E-02 |
| 2410014A08Rik | 1436508_at | E05.092.044_10 | 2.71E-06 | 0 | 7.2 | 6.53 | Y | 2.44E-02 |
| 9130011J15Rik | 1426647_at | E08.054.070_10 | 2.71E-06 | 0 | 5.13 | 5.62 | Y | 2.50E-04 |
| Amacr | 1417208_at | E15.013.193_10 | 2.71E-06 | 0.001 | 6.41 | 5.74 | Y | 1.79E-02 |
| Pink1 | 1441937_s_at | E04.141.582_10 | 2.73E-06 | 0.001 | 6 | 6.78 | Y | 4.25E-02 |
| Bms1 | 1447679_s_at | E06.126.705_10 | 2.75E-06 | 0.001 | 10.34 | 9.83 | Y | 1.80E-03 |
| Prpf4 | 1429724_at | E05.032.425_10 | 2.77E-06 | 0.001 | 7.22 | 6.93 | N | 3.30E-01 |
| Capn7 | 1423096_at | E14.023.871_10 | 2.78E-06 | 0 | 8.63 | 8.2 | Y | 1.85E-03 |
| Gstk1 | 1452823_at | E06.049.290_10 | 2.82E-06 | 0.001 | 6.31 | 7.45 | N | 7.40E-04 |
| Pdap1 | 1434019_at | E05.138.555_10 | 2.83E-06 | 0 | 9.69 | 9.32 | Y | 6.15E-02 |
| Isoc2b | 1418537_at | E07.002.028_10 | 2.87E-06 | 0 | 4.2 | 4.75 | Y | 6.93E-05 |
| Slc35f5 | 1452059_at | E01.125.571_10 | 2.87E-06 | 0 | 8.21 | 7.82 | Y | 9.99E-02 |
| Zswim3 | 1428313_at | E02.172.454_10 | 2.90E-06 | 0 | 5.07 | 5.41 | N | 1.52E-02 |
| Imp4 | 1426214_at | E01.042.090_10 | 2.92E-06 | 0 | 5.02 | 4.52 | Y | 7.51E-02 |
| Ifi35 | 1459151_x_at | E11.101.632_10 | 2.93E-06 | 0.001 | 7.47 | 6.77 | Y | 7.64E-04 |
| Rcor3 | 1428343_at | E01.187.751_10 | 2.95E-06 | 0.001 | 7.33 | 6.81 | N | 2.74E-01 |
| Ttc14 | 1452926_at | E03.019.137_10 | 2.95E-06 | 0 | 6.48 | 6.19 | Y | 8.33E-05 |
| Stx17 | 1431929_a_at | E04.040.685_10 | 3.01E-06 | 0 | 6.78 | 6.39 | Y | 7.79E-03 |
| Camta2 | 1426901_s_at | E06.032.288_10 | 3.05E-06 | 0.001 | 7.39 | 6.99 | N | 2.74E-01 |
| Def8 | 1415853_at | E08.129.275_10 | 3.05E-06 | 0.001 | 6.93 | 6.56 | Y | 1.11E-01 |
| Thg1l | 1432393_a_at | E11.036.485_10 | 3.06E-06 | 0.001 | 4.92 | 5.53 | Y | 9.72E-03 |
| Sav1 | 1416075_at | E12.061.769_10 | 3.08E-06 | 0.001 | 8.47 | 7.93 | Y | 2.00E-01 |
| Ncapg | 1429171_a_at | E05.044.682_10 | 3.08E-06 | 0 | 7.05 | 6.46 | N | 1.71E-01 |
| Ssr1 | 1441327_a_at | E10.106.427_10 | 3.12E-06 | 0 | 5.02 | 4.53 | N | 1.13E-01 |
| Dhdds | 1450654_a_at | E04.141.582_10 | 3.13E-06 | 0 | 6.76 | 6.34 | N | 1.39E-01 |
| Ap1gbp1 | 1457695_at | E11.089.228_10 | 3.24E-06 | 0 | 5.63 | 5.03 | Y | 2.97E-05 |
| Ddx49 | 1435681_s_at | E08.048.274_10 | 3.27E-06 | 0.001 | 4.92 | 5.49 | N | 2.53E-04 |
| Plscr4 | 1433626_at | E09.094.015_10 | 3.28E-06 | 0.001 | 3.71 | 4.9 | Y | 1.07E-03 |
| Calr | 1417606_a_at | E08.091.178_10 | 3.35E-06 | 0.001 | 11.47 | 11 | Y | 6.12E-05 |
| Endogl1 | 1436022_at | E09.099.776_10 | 3.38E-06 | 0.002 | 6.36 | 5.98 | Y | 1.22E-03 |
| N6amt2 | 1418386_at | E14.051.169_10 | 3.38E-06 | 0.001 | 7.21 | 6.69 | Y | 6.92E-04 |
| Hyls1 | 1431315_at | E09.034.366_10 | 3.47E-06 | 0 | 4.68 | 5.34 | N | 5.11E-03 |
| Tdp1 | 1426492_at | E12.105.658_10 | 3.48E-06 | 0.001 | 6.58 | 6.06 | N | 7.14E-03 |
| 2310061C15Rik | 1427921_s_at | E08.129.275_10 | 3.48E-06 | 0.002 | 7.39 | 6.93 | Y | 1.62E-01 |
| 1110019K23Rik | 1429601_x_at | E05.044.682_10 | 3.50E-06 | 0 | 6.51 | 5.96 | Y | 1.36E-01 |
| Ankrd39 | 1452933_at | E01.033.082_10 | 3.52E-06 | 0 | 5.59 | 5.2 | Y | 3.16E-03 |
| Cdkn2aip | 1429320_at | E08.054.070_10 | 3.56E-06 | 0 | 5.16 | 4.7 | Y | 1.97E-02 |
| 2410014A08Rik | 1436509_at | E05.103.695_10 | 3.59E-06 | 0 | 7.8 | 7.31 | Y | 2.85E-01 |
| Afg3l2 | 1427207_s_at | E18.056.581_10 | 3.59E-06 | 0 | 7.29 | 6.89 | Y | 4.25E-02 |
| Lmbr1 | 1442371_at | E05.032.425_10 | 3.61E-06 | 0 | 4.36 | 3.83 | Y | 4.78E-05 |
| Ikbkb | 1426207_at | E08.023.645_10 | 3.63E-06 | 0 | 9.09 | 8.65 | Y | 9.59E-04 |
| Sod3 | 1417634_at | E05.092.044_10 | 3.63E-06 | 0.001 | 6.3 | 5.43 | N | 2.41E-01 |
| Klf10 | 1416029_at | E15.087.204_10 | 3.66E-06 | 0 | 8.86 | 8.12 | Y | 2.20E-01 |
| Noc2l | 1424323_at | E04.156.084_10 | 3.67E-06 | 0.001 | 7.02 | 6.39 | Y | 5.55E-04 |
| Slc35b4 | 1416548_at | E06.032.288_10 | 3.72E-06 | 0 | 6.21 | 5.62 | Y | 5.49E-04 |
| Acaa1b | 1416947_s_at | E09.099.776_10 | 3.83E-06 | 0.001 | 7.83 | 7.51 | N | 2.17E-02 |
| Nek2 | 1437580_s_at | E01.187.751_10 | 3.93E-06 | 0 | 7.57 | 6.7 | N | 4.24E-02 |
| Jmjd3 | 1456610_at | E11.052.938_10 | 3.93E-06 | 0.003 | 7.95 | 8.49 | Y | 1.95E-01 |
| Mgat4a | 1435641_at | E16.084.417_10 | 3.95E-06 | 0.002 | 6.73 | 5.93 | Y | 2.00E-01 |
| Mettl7b | 1416980_at | E11.052.938_10 | 3.97E-06 | 0 | 3.8 | 4.16 | Y | 1.69E-01 |
| Idh3a | 1422500_at | E09.052.894_10 | 3.97E-06 | 0 | 5.62 | 5.1 | Y | 2.13E-02 |
| Rab11fip5 | 1427405_s_at | E06.092.041_10 | 4.01E-06 | 0 | 7.38 | 6.66 | Y | 1.39E-04 |
| Saa2 | 1449326_x_at | E07.021.899_10 | 4.02E-06 | 0 | 3.22 | 3.93 | N | 1.33E-01 |
| Mtap6 | 1457316_at | E07.116.345_10 | 4.05E-06 | 0 | 4.51 | 5.15 | Y | 1.62E-02 |
| Zfp192 | 1455778_at | E13.014.119_10 | 4.07E-06 | 0 | 6.72 | 6.19 | Y | 1.28E-02 |
| Appl1 | 1455159_at | E14.023.871_10 | 4.09E-06 | 0 | 7.25 | 6.9 | Y | 3.37E-03 |
| Cad | 1452830_s_at | E05.023.497_10 | 4.09E-06 | 0.001 | 7.56 | 6.88 | Y | 3.06E-03 |
| Pi4k2b | 1449862_a_at | E05.044.682_10 | 4.12E-06 | 0 | 8.24 | 7.42 | Y | 1.49E-03 |
| Slc30a9 | 1426668_at | E05.044.682_10 | 4.15E-06 | 0 | 8.13 | 7.74 | Y | 2.44E-02 |
| Rnf7 | 1456600_a_at | E09.102.375_10 | 4.23E-06 | 0 | 9.3 | 8.95 | Y | 5.37E-03 |
| Nab1 | 1417624_at | E01.042.090_10 | 4.25E-06 | 0 | 10.05 | 9.61 | Y | 6.74E-02 |
| 6030408C04Rik | 1455355_at | E06.046.553_10 | 4.26E-06 | 0 | 5.66 | 6.19 | N | 3.12E-01 |
| Rcbtb1 | 1456433_at | E14.093.815_10 | 4.30E-06 | 0 | 6.95 | 6.46 | Y | 1.02E-01 |
| Ric8 | 1455809_x_at | E07.133.429_10 | 4.36E-06 | 0 | 8.73 | 8.04 | Y | 5.20E-02 |
| BC019943 | 1424361_at | E08.020.455_10 | 4.39E-06 | 0.001 | 6.05 | 5.69 | Y | 4.01E-02 |
| Ms4a8a | 1418797_at | E19.005.316_10 | 4.40E-06 | 0.001 | 3.31 | 4.2 | N | 3.91E-03 |
| Tchp | 1438293_at | E05.107.059_10 | 4.42E-06 | 0.002 | 5.88 | 6.36 | Y | 6.25E-02 |
| Mki67ip | 1424001_at | E01.125.571_10 | 4.43E-06 | 0.002 | 8.14 | 7.58 | Y | 1.02E-02 |
| Zfp294 | 1452611_at | E16.084.417_10 | 4.44E-06 | 0 | 5.11 | 4.67 | Y | 1.39E-03 |
| D4Ertd22e | 1434482_at | E04.141.582_10 | 4.66E-06 | 0 | 8 | 7.54 | Y | 7.35E-02 |
| Minpp1 | 1423265_at | E19.055.858_10 | 4.76E-06 | 0.001 | 7.33 | 6.75 | Y | 9.53E-04 |
| Cxcl16 | 1418718_at | E09.080.375_10 | 4.81E-06 | 0 | 8.81 | 8.21 | Y | 2.21E-01 |
| Dctn4 | 1420863_at | E18.060.777_10 | 4.88E-06 | 0 | 5.39 | 5.93 | Y | 2.44E-01 |
| Cyb5r1 | 1424048_a_at | E01.135.010_10 | 4.92E-06 | 0.002 | 7.71 | 7.27 | N | 2.22E-02 |
| Cdca5 | 1416802_a_at | E19.005.316_10 | 4.95E-06 | 0.001 | 7.82 | 7.2 | N | 7.64E-04 |
| 2810422J05Rik | 1416624_a_at | E08.054.070_10 | 5.06E-06 | 0.002 | 13.95 | 13.78 | Y | 4.41E-05 |
| Cpd | 1418018_at | E11.089.228_10 | 5.07E-06 | 0 | 7.85 | 8.77 | Y | 4.74E-02 |
| Amotl1 | 1428785_at | E09.034.366_10 | 5.11E-06 | 0.003 | 9.43 | 8.8 | Y | 8.74E-04 |
| Apbb3 | 1424457_at | E18.053.479_10 | 5.13E-06 | 0 | 6.05 | 6.56 | Y | 1.17E-04 |
| Trim25 | 1425974_a_at | E11.095.195_10 | 5.24E-06 | 0 | 8.5 | 7.83 | Y | 1.22E-02 |
| 1200011I18Rik | 1454998_at | E14.093.815_10 | 5.25E-06 | 0 | 7.53 | 7.15 | Y | 2.24E-03 |
| 2810457I06Rik | 1429475_at | E09.034.366_10 | 5.25E-06 | 0.001 | 6 | 6.63 | Y | 1.19E-04 |
| Psma6 | 1416506_at | E12.053.286_10 | 5.26E-06 | 0 | 10.8 | 10.46 | Y | 2.93E-01 |
| Lztfl1 | 1428974_s_at | E09.099.776_10 | 5.43E-06 | 0 | 5.05 | 5.58 | Y | 2.06E-01 |
| Rps9 | 1434624_x_at | E07.002.028_10 | 5.43E-06 | 0 | 6.53 | 6.95 | Y | 2.35E-04 |
| Ube1l2 | 1428944_at | E05.085.774_10 | 5.44E-06 | 0 | 8.53 | 7.97 | Y | 6.15E-02 |
| Endod1 | 1426540_at | E09.034.366_10 | 5.56E-06 | 0 | 7.76 | 6.94 | Y | 2.92E-02 |
| Hsp90aa1 | 1438902_a_at | E12.092.013_10 | 5.66E-06 | 0.001 | 11.54 | 11.23 | Y | 9.53E-02 |
| Gnptab | 1435335_a_at | E10.102.359_10 | 5.73E-06 | 0.001 | 7.36 | 6.72 | Y | 1.62E-02 |
| Foxo1 | 1416982_at | E03.066.854_10 | 5.73E-06 | 0 | 6.66 | 6.11 | Y | 1.63E-03 |
| Ghitm | 1415882_at | E14.023.871_10 | 5.88E-06 | 0 | 10.39 | 10.09 | Y | 1.11E-01 |
| Rnasel | 1426603_at | E01.144.066_10 | 6.00E-06 | 0 | 6.92 | 6.18 | Y | 4.49E-03 |
| Rab35 | 1433922_at | E05.107.059_10 | 6.20E-06 | 0 | 8.15 | 7.85 | Y | 1.22E-03 |
| Setd2 | 1428555_at | E09.094.015_10 | 6.33E-06 | 0.002 | 7.75 | 8.03 | N | 3.05E-01 |
| Cnot6 | 1426685_a_at | E11.052.938_10 | 6.39E-06 | 0 | 9.27 | 8.83 | Y | 3.05E-02 |
| Zfp263 | 1447432_s_at | E16.018.064_10 | 6.42E-06 | 0.001 | 7.39 | 6.65 | Y | 2.94E-02 |
| Dctn6 | 1416499_a_at | E08.054.070_10 | 6.47E-06 | 0 | 8.66 | 8.19 | Y | 1.08E-01 |
| Tpbg | 1423311_s_at | E09.080.375_10 | 6.48E-06 | 0 | 8.43 | 7.39 | Y | 3.11E-03 |
| H2afj | 1424772_at | E06.131.131_10 | 6.49E-06 | 0 | 5.9 | 5.3 | Y | 5.20E-02 |
| Dnaja4 | 1434196_at | E09.052.894_10 | 6.56E-06 | 0 | 6.3 | 5.78 | Y | 8.68E-03 |
| Pon3 | 1419298_at | E06.001.969_10 | 6.56E-06 | 0 | 7.26 | 6.25 | Y | 7.46E-04 |
| Rpp14 | 1419461_at | E14.041.725_10 | 6.56E-06 | 0.001 | 8.15 | 7.83 | Y | 4.95E-02 |
| Ripk3 | 1448449_at | E14.051.169_10 | 6.58E-06 | 0 | 7.56 | 6.9 | Y | 5.02E-02 |
| Tm2d2 | 1439234_a_at | E08.023.645_10 | 6.58E-06 | 0.003 | 6.71 | 6.21 | Y | 6.47E-02 |
| Ndufc1 | 1448284_a_at | E03.066.854_10 | 6.60E-06 | 0.001 | 10.45 | 9.69 | Y | 2.84E-06 |
| Xbp1 | 1420886_a_at | E11.022.164_10 | 6.62E-06 | 0.001 | 10.45 | 9.74 | Y | 3.70E-03 |
| Wtap | 1454805_at | E17.022.410_10 | 6.66E-06 | 0 | 8.68 | 8.29 | Y | 3.21E-03 |
| D2Ertd750e | 1420081_s_at | E02.112.734_10 | 6.72E-06 | 0.001 | 8.06 | 7.5 | N | 2.98E-01 |
| Mfap1a | 1419370_a_at | E02.112.734_10 | 6.74E-06 | 0.002 | 7.56 | 7.28 | Y | 1.22E-02 |
| Acad8 | 1419261_at | E09.017.669_10 | 6.83E-06 | 0 | 4.59 | 4.24 | Y | 2.30E-03 |
| Vti1a | 1419189_at | E19.055.858_10 | 6.83E-06 | 0 | 6.29 | 5.96 | Y | 5.15E-02 |
| Cln5 | 1426886_at | E14.111.590_10 | 6.86E-06 | 0.001 | 7.47 | 7.09 | Y | 4.22E-02 |
| Dclk1 | 1435940_at | E03.019.137_10 | 6.94E-06 | 0 | 4.85 | 5.65 | N | 2.92E-02 |
| Pfkfb3 | 1456676_a_at | E02.005.251_10 | 7.01E-06 | 0 | 5.04 | 4.39 | N | 2.18E-01 |
| Bst2 | 1424921_at | E08.054.070_10 | 7.03E-06 | 0 | 6.43 | 7.63 | Y | 7.36E-03 |
| Ddx1 | 1415915_at | E12.070.033_10 | 7.03E-06 | 0.003 | 9.74 | 9.4 | Y | 3.33E-01 |
| Atp8a1 | 1433965_at | E06.032.288_10 | 7.04E-06 | 0.001 | 4.99 | 4.48 | N | 1.57E-01 |
| Zfp516 | 1455944_at | E18.077.468_10 | 7.07E-06 | 0.001 | 5.74 | 5.31 | Y | 1.13E-02 |
| Rpl21 | 1423070_at | E05.138.555_10 | 7.09E-06 | 0 | 5.52 | 4.93 | Y | 6.13E-03 |
| Nola3 | 1423211_at | E02.112.734_10 | 7.10E-06 | 0.002 | 9.11 | 8.58 | Y | 2.69E-01 |
| Daxx | 1419026_at | E06.032.288_10 | 7.18E-06 | 0 | 7.72 | 7.49 | Y | 7.57E-02 |
| Krr1 | 1428084_at | E10.118.344_10 | 7.27E-06 | 0.001 | 8.35 | 7.96 | Y | 1.26E-01 |
| 2610510H03Rik | 1460037_at | E02.112.734_10 | 7.27E-06 | 0.001 | 9.11 | 8.77 | Y | 2.05E-01 |
| BC003331 | 1426089_a_at | E01.144.066_10 | 7.34E-06 | 0 | 7.77 | 7.3 | Y | 4.40E-02 |
| Mnda | 1452348_s_at | E01.169.742_10 | 7.41E-06 | 0.004 | 6.97 | 8.2 | Y | 3.07E-02 |
| Stat5b | 1422103_a_at | E06.001.969_10 | 7.44E-06 | 0.001 | 6.42 | 6.01 | Y | 3.27E-01 |
| Cog3 | 1434654_at | E14.093.815_10 | 7.48E-06 | 0 | 7.88 | 7.59 | Y | 1.32E-01 |
| Gm8126 | 1440815_x_at | E14.019.954_10 | 7.50E-06 | 0.002 | 2.47 | 4.27 | Y | 1.56E-04 |
| Tmem33 | 1436028_at | E05.044.682_10 | 7.51E-06 | 0 | 8.39 | 8.1 | Y | 8.59E-02 |
| Zfp410 | 1451150_at | E12.073.179_10 | 7.56E-06 | 0.003 | 6.7 | 6.41 | Y | 1.18E-01 |
| Bin3 | 1417691_at | E14.094.324_10 | 7.61E-06 | 0.005 | 8.72 | 8.42 | Y | 7.80E-02 |
| Trove2 | 1423433_at | E01.135.010_10 | 7.63E-06 | 0.002 | 6.72 | 6.2 | Y | 3.09E-03 |
| Sgms2 | 1429029_at | E03.140.010_10 | 7.64E-06 | 0.004 | 5.45 | 6.32 | N | 1.01E-01 |
| Rogdi | 1451421_a_at | E16.018.064_10 | 7.87E-06 | 0.001 | 7.09 | 6.84 | Y | 5.91E-04 |
| Birc6 | 1427488_a_at | E17.087.010_10 | 7.88E-06 | 0.001 | 4.95 | 5.51 | N | 3.13E-01 |
| Gpr160 | 1453072_at | E03.026.032_10 | 7.90E-06 | 0.001 | 6.82 | 6.05 | Y | 3.20E-02 |
| Trim26 | 1424929_a_at | E17.022.410_10 | 8.18E-06 | 0.003 | 6.99 | 6.56 | Y | 6.92E-04 |
| Ncoa3 | 1448027_at | E06.032.288_10 | 8.24E-06 | 0.002 | 6.74 | 6.35 | Y | 1.94E-01 |
| Sept8 | 1426802_at | E04.106.454_10 | 8.35E-06 | 0.001 | 6.59 | 5.93 | N | 3.53E-02 |
| Rps6 | 1416141_a_at | E04.075.609_10 | 8.38E-06 | 0.004 | 13.87 | 13.7 | Y | 7.14E-03 |
| Wsb2 | 1421847_at | E05.107.059_10 | 8.42E-06 | 0.001 | 9.18 | 8.9 | Y | 4.33E-02 |
| Gnb1 | 1417432_a_at | E04.156.084_10 | 8.43E-06 | 0.001 | 10.43 | 10.02 | Y | 6.79E-03 |
| Terf2ip | 1426049_a_at | E08.112.069_10 | 8.48E-06 | 0.002 | 5.95 | 5.56 | Y | 1.28E-02 |
| Klhdc5 | 1426988_at | E06.131.131_10 | 8.64E-06 | 0.004 | 6.94 | 6.33 | N | 2.54E-01 |
| Zfp384 | 1438047_at | E09.099.776_10 | 8.69E-06 | 0.001 | 8.41 | 8.65 | Y | 1.69E-01 |
| Atp5j2 | 1416269_at | E05.138.555_10 | 8.74E-06 | 0.001 | 11.04 | 10.63 | Y | 3.88E-02 |
| Dnajc21 | 1460711_at | E15.013.193_10 | 8.80E-06 | 0.003 | 7.06 | 6.5 | Y | 5.02E-02 |
| Zfp213 | 1433705_at | E17.022.410_10 | 8.89E-06 | 0.002 | 5.24 | 4.83 | Y | 7.14E-03 |
| Slc25a38 | 1424418_at | E09.099.776_10 | 8.90E-06 | 0.001 | 6.5 | 6.26 | Y | 6.13E-03 |
| Ets1 | 1452163_at | E09.034.366_10 | 8.99E-06 | 0.001 | 8.22 | 7.55 | Y | 5.17E-04 |
| Klhdc3 | 1415991_a_at | E06.007.946_10 | 9.09E-06 | 0.004 | 9.8 | 9.5 | N | 3.35E-01 |
| Zfhx3 | 1429725_at | E08.109.032_10 | 9.23E-06 | 0.001 | 4.35 | 4.98 | N | 7.51E-02 |
| Prkar1b | 1434325_x_at | E05.138.555_10 | 9.28E-06 | 0.005 | 4.58 | 5.66 | N | 3.88E-02 |
| Asah3l | 1451355_at | E13.001.439_10 | 9.30E-06 | 0.003 | 5.94 | 5.38 | N | 2.78E-02 |
| Canx | 1428935_at | E11.052.938_10 | 9.34E-06 | 0.001 | 7.03 | 6.56 | Y | 1.75E-02 |
| Ripk1 | 1439273_at | E13.037.637_10 | 9.54E-06 | 0.001 | 6.24 | 6.67 | Y | 7.61E-02 |
| Qtrtd1 | 1421320_a_at | E16.047.738_10 | 9.56E-06 | 0 | 4.3 | 4.69 | N | 3.40E-01 |
| D15Wsu75e | 1460689_at | E15.057.403_10 | 9.64E-06 | 0.001 | 7.84 | 7.29 | Y | 2.11E-01 |
| Itgb1 | 1426918_at | E08.129.275_10 | 9.70E-06 | 0.002 | 10.46 | 9.95 | Y | 3.59E-02 |
| Gan | 1439615_at | E08.129.275_10 | 9.87E-06 | 0.002 | 5.47 | 4.96 | N | 1.22E-01 |
| Abce1 | 1416015_s_at | E08.091.178_10 | 9.91E-06 | 0.001 | 9.37 | 8.81 | Y | 5.93E-04 |
| Decr2 | 1423495_at | E17.022.410_10 | 1.01E-05 | 0 | 5.7 | 5.2 | Y | 1.95E-01 |
| Ifitm1 | 1424254_at | E07.133.429_10 | 1.01E-05 | 0.001 | 10.46 | 9.34 | Y | 1.02E-01 |
| Rpl23 | 1454859_a_at | E11.101.632_10 | 1.02E-05 | 0 | 13.77 | 13.46 | Y | 8.01E-04 |
| Kctd3 | 1436811_at | E01.187.751_10 | 1.02E-05 | 0 | 7.09 | 6.74 | Y | 1.38E-02 |
| Sfxn5 | 1436618_at | E09.102.375_10 | 1.04E-05 | 0.001 | 5.13 | 5.85 | N | 1.25E-01 |
| Tmem69 | 1457680_a_at | E04.121.681_10 | 1.04E-05 | 0.002 | 5.63 | 6.01 | Y | 4.58E-03 |
| Klhl23 | 1435743_at | E02.056.057_10 | 1.07E-05 | 0 | 3.92 | 4.62 | Y | 4.69E-02 |
| Dbf4 | 1418334_at | E05.044.682_10 | 1.07E-05 | 0.002 | 8.36 | 7.84 | N | 6.73E-02 |
| Itpr2 | 1427287_s_at | E06.147.529_10 | 1.08E-05 | 0.003 | 6.3 | 6.91 | Y | 9.60E-03 |
| Gpr123 | 1459750_s_at | E07.136.725_10 | 1.08E-05 | 0 | 2.94 | 4.22 | N | 6.84E-05 |
| Ndrg1 | 1450977_s_at | E09.034.366_10 | 1.08E-05 | 0 | 9.52 | 8.51 | N | 3.13E-01 |
| Sh3rf1 | 1421271_at | E08.048.274_10 | 1.08E-05 | 0.001 | 5.9 | 6.41 | N | 2.74E-02 |
| Ublcp1 | 1415788_at | E11.036.485_10 | 1.09E-05 | 0.001 | 8.28 | 7.9 | Y | 1.65E-02 |
| Ripk5 | 1436300_at | E01.144.066_10 | 1.09E-05 | 0.001 | 6.98 | 6.53 | Y | 1.00E-01 |
| Sod2 | 1454976_at | E17.022.410_10 | 1.09E-05 | 0.001 | 6.57 | 5.81 | Y | 4.41E-05 |
| Alkbh1 | 1434660_at | E12.070.033_10 | 1.10E-05 | 0.002 | 7.06 | 6.72 | Y | 3.59E-02 |
| 5430435G22Rik | 1424987_at | E01.144.066_10 | 1.10E-05 | 0.002 | 6.24 | 6.77 | Y | 2.14E-02 |
| Lztfl1 | 1417170_at | E09.099.776_10 | 1.11E-05 | 0.001 | 7.66 | 8.09 | Y | 1.05E-01 |
| Ndrg1 | 1423413_at | E09.034.366_10 | 1.11E-05 | 0.002 | 9.33 | 8.28 | N | 3.34E-01 |
| 1810063B07Rik | 1427905_at | E14.023.871_10 | 1.11E-05 | 0.001 | 6.94 | 6.55 | N | 3.71E-03 |
| Usp48 | 1424056_at | E04.141.582_10 | 1.12E-05 | 0 | 6.99 | 7.42 | Y | 4.22E-02 |
| Rbms2 | 1433979_at | E10.118.344_10 | 1.12E-05 | 0.002 | 7.51 | 6.98 | Y | 8.57E-02 |
| Jmjd2c | 1424458_at | E04.075.609_10 | 1.14E-05 | 0.002 | 6.09 | 5.7 | Y | 2.85E-03 |
| Incenp | 1439436_x_at | E19.005.316_10 | 1.14E-05 | 0.001 | 8.89 | 8.36 | N | 6.27E-04 |
| Akr1b8 | 1448894_at | E06.032.288_10 | 1.15E-05 | 0.001 | 10.13 | 10.94 | Y | 1.71E-04 |
| Siva1 | 1426323_x_at | E12.112.981_10 | 1.15E-05 | 0 | 7.98 | 7.53 | Y | 6.67E-02 |
| Dnlz | 1435528_at | E02.019.239_10 | 1.15E-05 | 0 | 6.49 | 6.12 | Y | 6.92E-04 |
| Btrc | 1425680_a_at | E19.055.858_10 | 1.17E-05 | 0 | 6.01 | 5.53 | Y | 1.40E-02 |
| Mpp5 | 1421064_at | E12.080.625_10 | 1.18E-05 | 0.001 | 7.46 | 6.82 | Y | 6.11E-02 |
| 5730455O13Rik | 1435810_at | E19.055.858_10 | 1.18E-05 | 0 | 6.2 | 5.71 | Y | 2.03E-02 |
| Sppl3 | 1451073_at | E05.107.059_10 | 1.19E-05 | 0.003 | 8.43 | 8.16 | Y | 1.12E-03 |
| Gsk3b | 1434439_at | E16.033.089_10 | 1.20E-05 | 0.002 | 5.24 | 5.73 | Y | 9.88E-03 |
| Cntn2 | 1435165_at | E01.135.010_10 | 1.21E-05 | 0.002 | 4.16 | 4.72 | Y | 7.97E-05 |
| Tusc4 | 1417046_at | E09.099.776_10 | 1.21E-05 | 0 | 6.75 | 6.43 | Y | 5.70E-02 |
| Nrp2 | 1435349_at | E01.029.882_10 | 1.21E-05 | 0.001 | 8.37 | 7.56 | Y | 1.35E-01 |
| Prkab2 | 1442001_at | E05.032.425_10 | 1.22E-05 | 0.002 | 5.53 | 5.24 | Y | 2.98E-01 |
| Tex2 | 1452213_at | E11.101.632_10 | 1.22E-05 | 0.001 | 6.64 | 6.08 | N | 1.10E-02 |
| Bet1l | 1422980_a_at | E07.133.429_10 | 1.23E-05 | 0.001 | 7.67 | 7.1 | Y | 1.93E-01 |
| 1110014J01Rik | 1448141_at | E15.073.732_10 | 1.23E-05 | 0.005 | 7.98 | 7.63 | Y | 8.47E-02 |
| Slc35d2 | 1453300_at | E09.094.015_10 | 1.24E-05 | 0.003 | 5.05 | 5.4 | Y | 3.13E-01 |
| Tgfbrap1 | 1428425_at | E01.016.840_10 | 1.24E-05 | 0.002 | 6.33 | 6.05 | N | 2.97E-01 |
| Tmco6 | 1451692_at | E18.053.479_10 | 1.25E-05 | 0.003 | 6.36 | 5.91 | Y | 1.55E-02 |
| Atad3a | 1437343_x_at | E04.141.582_10 | 1.26E-05 | 0.001 | 8.73 | 8.14 | Y | 1.68E-02 |
| Bxdc2 | 1423841_at | E15.024.918_10 | 1.26E-05 | 0 | 5.68 | 5 | N | 6.11E-02 |
| Nup155 | 1418727_at | E15.024.918_10 | 1.29E-05 | 0.002 | 7.04 | 6.54 | Y | 9.54E-02 |
| Hn1l | 1433696_at | E05.138.555_10 | 1.29E-05 | 0.001 | 8.07 | 7.62 | Y | 2.89E-01 |
| Hsp90aa1 | 1426645_at | E12.092.013_10 | 1.29E-05 | 0.002 | 11.89 | 11.47 | Y | 2.73E-01 |
| Slu7 | 1447805_s_at | E11.036.485_10 | 1.29E-05 | 0.003 | 7.09 | 6.81 | Y | 3.09E-03 |
| Fuca2 | 1440313_at | E06.104.373_10 | 1.30E-05 | 0.001 | 5.07 | 5.25 | N | 7.79E-02 |
| Homer1 | 1437363_at | E13.056.787_10 | 1.30E-05 | 0.001 | 6.97 | 6.49 | Y | 1.43E-02 |
| Tada3l | 1417467_a_at | E06.113.085_10 | 1.31E-05 | 0 | 6.76 | 6.46 | N | 7.57E-02 |
| Pros1 | 1426246_at | E16.067.649_10 | 1.34E-05 | 0.003 | 7.75 | 6.92 | Y | 2.58E-02 |
| Atm | 1428830_at | E09.052.894_10 | 1.34E-05 | 0.003 | 6.65 | 6.3 | Y | 1.40E-02 |
| 4930535B03Rik | 1438028_at | E03.094.477_10 | 1.36E-05 | 0 | 6.37 | 5.97 | N | 7.57E-04 |
| Lmnb1 | 1423520_at | E18.056.581_10 | 1.36E-05 | 0.001 | 7.36 | 6.74 | Y | 1.82E-01 |
| Ier3ip1 | 1417424_at | E18.077.468_10 | 1.37E-05 | 0 | 8.42 | 7.86 | Y | 1.39E-03 |
| Dlgap5 | 1438811_at | E14.041.725_10 | 1.37E-05 | 0.001 | 4.87 | 5.43 | N | 3.37E-03 |
| Pcmtd1 | 1429429_s_at | E01.001.113_10 | 1.38E-05 | 0.002 | 7.79 | 7.35 | Y | 3.09E-03 |
| Ivd | 1418238_at | E02.112.734_10 | 1.38E-05 | 0.006 | 7.23 | 6.8 | Y | 1.95E-02 |
| Abl2 | 1455682_at | E01.160.825_10 | 1.39E-05 | 0.001 | 7.01 | 6.39 | Y | 8.55E-02 |
| March7 | 1436893_a_at | E02.056.057_10 | 1.39E-05 | 0.005 | 8.37 | 7.89 | Y | 7.72E-03 |
| Fbxo39 | 1443621_at | E11.070.437_10 | 1.39E-05 | 0.003 | 4.35 | 5.3 | Y | 8.05E-06 |
| Pcbd2 | 1452621_at | E01.001.113_10 | 1.40E-05 | 0.002 | 7.89 | 8.3 | Y | 1.99E-02 |
| Pcgf2 | 1420645_at | E09.094.015_10 | 1.40E-05 | 0.003 | 4.68 | 4.93 | N | 1.92E-01 |
| Sys1 | 1450057_at | E02.172.454_10 | 1.40E-05 | 0.002 | 7.54 | 7.16 | Y | 2.11E-02 |
| Map4k4 | 1434184_s_at | E01.016.840_10 | 1.40E-05 | 0.002 | 9.16 | 8.84 | N | 3.36E-01 |
| Psmc3ip | 1442058_s_at | E11.101.632_10 | 1.41E-05 | 0.001 | 7 | 6.18 | Y | 1.57E-04 |
| Mlxip | 1426979_at | E06.032.288_10 | 1.43E-05 | 0.001 | 7.53 | 7.18 | Y | 3.00E-01 |
| Hk1 | 1420901_a_at | E07.133.429_10 | 1.48E-05 | 0.001 | 6.97 | 7.76 | N | 2.85E-01 |
| Rnf168 | 1455585_at | E16.033.089_10 | 1.48E-05 | 0.001 | 6.89 | 6.59 | Y | 1.07E-01 |
| Dpy30 | 1423767_at | E06.032.288_10 | 1.48E-05 | 0.005 | 8.85 | 9.21 | Y | 1.36E-01 |
| Acot2 | 1422996_at | E12.080.625_10 | 1.48E-05 | 0.004 | 3.87 | 4.34 | N | 3.53E-03 |
| Vkorc1l1 | 1429092_at | E05.138.555_10 | 1.49E-05 | 0.002 | 7.04 | 6.59 | Y | 1.07E-01 |
| Clptm1 | 1416883_at | E14.116.830_10 | 1.53E-05 | 0.001 | 9.49 | 9.7 | Y | 1.79E-01 |
| Tbl1x | 1434643_at | E02.112.734_10 | 1.54E-05 | 0.002 | 7.68 | 7.3 | Y | 1.90E-01 |
| Plekha8 | 1436128_at | E06.032.288_10 | 1.55E-05 | 0.003 | 5.23 | 5.94 | Y | 4.03E-04 |
| Slc39a6 | 1424675_at | E09.052.894_10 | 1.56E-05 | 0.003 | 7.58 | 6.98 | N | 3.25E-01 |
| Gli3 | 1456067_at | E13.014.119_10 | 1.56E-05 | 0 | 7.75 | 7.12 | Y | 1.63E-03 |
| Peli2 | 1437181_at | E14.041.725_10 | 1.56E-05 | 0.001 | 4.91 | 4.25 | Y | 8.33E-05 |
| Gna13 | 1422556_at | E11.117.260_10 | 1.58E-05 | 0.001 | 7.89 | 8.49 | Y | 2.64E-02 |
| Soat1 | 1417697_at | E03.006.520_10 | 1.59E-05 | 0.001 | 6.3 | 7.1 | Y | 2.96E-01 |
| Tpm2 | 1449577_x_at | E02.015.712_10 | 1.60E-05 | 0.001 | 8.05 | 8.7 | N | 1.22E-01 |
| Tmem140 | 1424354_at | E06.032.288_10 | 1.60E-05 | 0.002 | 6.41 | 5.45 | N | 5.14E-02 |
| 2310045N01Rik | 1429079_a_at | E08.048.274_10 | 1.62E-05 | 0.001 | 8.55 | 9 | N | 3.05E-04 |
| Dnajc3a | 1419162_s_at | E15.013.193_10 | 1.62E-05 | 0.002 | 6.41 | 6.82 | Y | 3.39E-01 |
| Myo9b | 1418031_at | E06.032.288_10 | 1.62E-05 | 0.002 | 8.44 | 8.13 | Y | 1.28E-01 |
| Txndc9 | 1424512_a_at | E06.032.288_10 | 1.63E-05 | 0.002 | 8.29 | 8.8 | N | 1.87E-01 |
| Centb2 | 1433561_at | E16.033.089_10 | 1.65E-05 | 0.003 | 8.79 | 8.36 | Y | 2.27E-03 |
| Pon2 | 1429019_s_at | E06.001.969_10 | 1.65E-05 | 0.005 | 8.76 | 8.33 | Y | 1.84E-02 |
| Acbd6 | 1452601_a_at | E09.116.239_10 | 1.66E-05 | 0 | 6.93 | 7.27 | Y | 2.19E-01 |
| 9130005N14Rik | 1417272_at | E05.092.044_10 | 1.66E-05 | 0.003 | 7.66 | 6.8 | Y | 3.94E-03 |
| Ngdn | 1428226_at | E14.023.871_10 | 1.68E-05 | 0.002 | 8.49 | 8.23 | N | 1.88E-01 |
| Topbp1 | 1452241_at | E09.102.375_10 | 1.69E-05 | 0.003 | 8.55 | 8.14 | Y | 4.29E-03 |
| Rce1 | 1418779_at | E19.005.316_10 | 1.70E-05 | 0.003 | 7.83 | 7.54 | Y | 1.20E-02 |
| Acad9 | 1453206_at | E03.019.137_10 | 1.73E-05 | 0.005 | 6.8 | 7.18 | Y | 1.92E-01 |
| Tmem38a | 1424178_at | E08.023.645_10 | 1.73E-05 | 0.001 | 4.69 | 5.19 | N | 7.52E-02 |
| Sbk1 | 1423978_at | E07.116.345_10 | 1.73E-05 | 0.003 | 4 | 4.35 | N | 2.18E-01 |
| Ormdl3 | 1419450_at | E04.075.609_10 | 1.75E-05 | 0.001 | 6.39 | 6.97 | Y | 4.69E-02 |
| Mfap3 | 1424721_at | E11.052.938_10 | 1.76E-05 | 0.001 | 7.73 | 7.1 | Y | 7.14E-03 |
| Als2cr4 | 1435661_at | E01.029.882_10 | 1.76E-05 | 0.003 | 6.57 | 5.68 | Y | 1.12E-01 |
| Dclk1 | 1450863_a_at | E03.019.137_10 | 1.76E-05 | 0.002 | 3.95 | 4.87 | N | 2.93E-01 |
| Elp2 | 1438179_s_at | E18.053.479_10 | 1.77E-05 | 0.001 | 9.84 | 9.31 | Y | 6.52E-04 |
| Pak2 | 1454887_at | E16.033.089_10 | 1.77E-05 | 0.005 | 8.91 | 8.55 | N | 1.82E-02 |
| Rdh11 | 1449209_a_at | E06.032.288_10 | 1.82E-05 | 0.001 | 6.36 | 6.94 | Y | 5.29E-02 |
| Kitl | 1415854_at | E09.052.894_10 | 1.82E-05 | 0.006 | 4.48 | 3.82 | N | 5.47E-02 |
| Fbxo36 | 1449418_s_at | E01.073.342_10 | 1.83E-05 | 0.002 | 5.49 | 5.08 | Y | 7.26E-03 |
| Ufd1l | 1418087_at | E16.018.064_10 | 1.83E-05 | 0.001 | 7.27 | 6.89 | Y | 1.02E-01 |
| Gfra1 | 1421973_at | E19.058.440_10 | 1.84E-05 | 0 | 3.67 | 4.43 | Y | 1.11E-04 |
| Gnptab | 1456620_at | E10.102.359_10 | 1.85E-05 | 0.003 | 7.01 | 6.34 | Y | 2.43E-03 |
| Dck | 1428838_a_at | E05.085.774_10 | 1.85E-05 | 0.004 | 6.03 | 5.42 | N | 8.87E-03 |
| Psme3 | 1418078_at | E04.075.609_10 | 1.87E-05 | 0.005 | 8.28 | 7.7 | Y | 3.35E-01 |
| Klc1 | 1428881_at | E12.099.141_10 | 1.88E-05 | 0.004 | 7.88 | 8.31 | Y | 6.67E-02 |
| 2410127L17Rik | 1429458_at | E19.023.477_10 | 1.88E-05 | 0.004 | 9.33 | 8.92 | Y | 3.30E-02 |
| Bst1 | 1449454_at | E05.044.682_10 | 1.89E-05 | 0.003 | 3.63 | 4.53 | N | 4.97E-03 |
| Iars2 | 1426735_at | E01.187.751_10 | 1.90E-05 | 0.001 | 7.32 | 6.8 | Y | 2.36E-02 |
| Brf2 | 1429561_at | E08.023.645_10 | 1.90E-05 | 0.001 | 5.93 | 5.65 | Y | 2.55E-01 |
| Dcun1d1 | 1452079_s_at | E06.032.288_10 | 1.90E-05 | 0.005 | 6.82 | 7.38 | N | 2.98E-01 |
| Atp6v1c1 | 1419546_at | E15.024.918_10 | 1.90E-05 | 0.006 | 8.29 | 7.78 | Y | 4.91E-02 |
| Zkscan1 | 1447944_at | E05.138.555_10 | 1.91E-05 | 0.004 | 5.17 | 4.55 | N | 1.14E-01 |
| Ttc4 | 1451192_a_at | E04.106.454_10 | 1.91E-05 | 0.004 | 6.92 | 6.44 | Y | 5.92E-03 |
| Cript | 1423486_at | E17.087.010_10 | 1.92E-05 | 0 | 9.7 | 9.26 | Y | 1.39E-04 |
| Dlgap4 | 1426465_at | E02.157.973_10 | 1.94E-05 | 0.003 | 7.8 | 7.44 | Y | 3.39E-01 |
| Rps10 | 1416719_a_at | E17.022.410_10 | 1.94E-05 | 0.002 | 13.1 | 12.88 | Y | 4.55E-02 |
| Metap2 | 1451048_at | E06.049.290_10 | 1.94E-05 | 0.002 | 4.28 | 4.99 | Y | 2.74E-01 |
| Stk11ip | 1431792_a_at | E09.102.375_10 | 1.94E-05 | 0.004 | 5.63 | 6.12 | Y | 2.42E-01 |
| Mbip | 1424405_at | E12.070.033_10 | 1.95E-05 | 0.001 | 7.01 | 6.63 | Y | 9.01E-02 |
| Vdac1 | 1437192_x_at | E11.052.938_10 | 1.96E-05 | 0.002 | 10.83 | 10.42 | Y | 5.11E-03 |
| Arl3 | 1450706_a_at | E19.055.858_10 | 1.97E-05 | 0.002 | 8.22 | 7.64 | Y | 1.25E-01 |
| Smyd2 | 1424760_a_at | E01.187.751_10 | 1.97E-05 | 0.001 | 8.66 | 8.17 | Y | 1.91E-01 |
| BC049806 | 1456320_at | E01.033.082_10 | 1.97E-05 | 0.001 | 6.93 | 6.21 | Y | 2.30E-01 |
| Ormdl1 | 1451219_at | E01.060.215_10 | 1.99E-05 | 0.005 | 8.41 | 7.89 | N | 2.55E-01 |
| Qk | 1425597_a_at | E17.022.410_10 | 1.99E-05 | 0.001 | 4.47 | 3.89 | N | 2.02E-03 |
| Lsm6 | 1437108_at | E08.112.069_10 | 2.00E-05 | 0.001 | 6.19 | 5.65 | Y | 6.88E-02 |
| Sco1 | 1430300_at | E11.070.437_10 | 2.01E-05 | 0.004 | 4.48 | 4.04 | Y | 3.94E-06 |
| Car4 | 1448949_at | E10.106.427_10 | 2.01E-05 | 0.002 | 5.39 | 6.69 | N | 4.00E-02 |
| Dnajc14 | 1437546_at | E10.118.344_10 | 2.01E-05 | 0.003 | 7.8 | 7.26 | Y | 1.86E-01 |
| Btaf1 | 1435953_at | E19.055.858_10 | 2.04E-05 | 0 | 7.19 | 6.75 | N | 1.25E-02 |
| Ugcgl1 | 1455839_at | E09.102.375_10 | 2.05E-05 | 0.004 | 8.61 | 8.95 | N | 3.16E-01 |
| Kitl | 1426152_a_at | E10.102.359_10 | 2.08E-05 | 0.006 | 4.73 | 5.36 | N | 5.05E-02 |
| Ubap1 | 1423185_a_at | E01.083.652_10 | 2.09E-05 | 0.003 | 7.48 | 7.26 | Y | 8.43E-02 |
| 9030612M13Rik | 1435163_at | E17.022.410_10 | 2.10E-05 | 0.004 | 6.68 | 6.05 | Y | 5.60E-04 |
| Bcl10 | 1418970_a_at | E03.147.739_10 | 2.11E-05 | 0.002 | 9.09 | 8.75 | N | 1.67E-04 |
| Slc15a4 | 1448385_at | E05.126.262_10 | 2.11E-05 | 0.002 | 7.67 | 7.31 | Y | 4.83E-03 |
| Rqcd1 | 1450887_at | E09.099.776_10 | 2.13E-05 | 0.001 | 7.93 | 8.24 | N | 2.93E-01 |
| Zfp458 | 1445824_at | E13.053.052_10 | 2.13E-05 | 0.002 | 4.29 | 4.91 | Y | 1.44E-03 |
| 1810063B05Rik | 1435864_a_at | E08.129.275_10 | 2.14E-05 | 0.005 | 8.32 | 7.75 | Y | 1.79E-01 |
| Rpl10a | 1431177_a_at | E06.032.288_10 | 2.15E-05 | 0.003 | 12.72 | 12.85 | Y | 2.48E-01 |
| Cmpk | 1423073_at | E04.106.454_10 | 2.20E-05 | 0.003 | 10.65 | 10.17 | Y | 2.50E-04 |
| Car4 | 1418094_s_at | E10.106.427_10 | 2.22E-05 | 0.002 | 5.77 | 6.97 | N | 3.27E-02 |
| Chek1 | 1439208_at | E09.034.366_10 | 2.24E-05 | 0.002 | 3.68 | 4.1 | N | 6.92E-04 |
| Hoxa3 | 1452421_at | E03.066.854_10 | 2.25E-05 | 0.001 | 4.99 | 5.27 | N | 3.38E-01 |
| Acly | 1439459_x_at | E11.101.632_10 | 2.25E-05 | 0.004 | 9.86 | 9.41 | Y | 1.22E-02 |
| Racgap1 | 1421546_a_at | E15.101.379_10 | 2.26E-05 | 0.004 | 7.07 | 6.22 | N | 5.99E-02 |
| Hnrnpu | 1423051_at | E09.099.776_10 | 2.27E-05 | 0.002 | 11.16 | 11.33 | Y | 1.35E-01 |
| Cyp51 | 1422533_at | E05.002.624_10 | 2.29E-05 | 0.002 | 7.4 | 6.3 | Y | 3.32E-02 |
| 0710008K08Rik | 1424311_at | E08.023.645_10 | 2.30E-05 | 0.004 | 8.75 | 8.42 | N | 1.14E-01 |
| AI931714 | 1455916_at | E08.088.925_10 | 2.36E-05 | 0.005 | 6.88 | 7.32 | Y | 1.18E-04 |
| Casp6 | 1415995_at | E03.147.739_10 | 2.36E-05 | 0.004 | 9.24 | 8.82 | N | 8.44E-02 |
| 0610011L14Rik | 1426017_a_at | E02.157.973_10 | 2.37E-05 | 0.004 | 6.53 | 6.32 | Y | 3.40E-01 |
| Utp6 | 1424500_at | E06.032.288_10 | 2.37E-05 | 0.003 | 7.49 | 8.11 | Y | 1.34E-01 |
| Cog1 | 1449053_s_at | E09.034.366_10 | 2.37E-05 | 0.002 | 6.86 | 7.13 | N | 1.86E-01 |
| Ints9 | 1436896_at | E14.067.774_10 | 2.37E-05 | 0.003 | 6.77 | 6.45 | Y | 2.94E-02 |
| Cdk2 | 1416873_a_at | E10.118.344_10 | 2.39E-05 | 0.006 | 7.29 | 6.95 | Y | 2.25E-01 |
| Ciapin1 | 1448140_at | E08.112.069_10 | 2.40E-05 | 0.005 | 7.45 | 7.04 | Y | 4.09E-02 |
| D030051N19Rik | 1454985_at | E06.007.946_10 | 2.41E-05 | 0.002 | 6.95 | 6.69 | Y | 6.17E-02 |
| Cspp1 | 1454989_at | E01.001.113_10 | 2.42E-05 | 0.006 | 7.27 | 6.74 | Y | 6.88E-04 |
| Apoc4 | 1418708_at | E07.021.899_10 | 2.44E-05 | 0.004 | 4.24 | 4.88 | Y | 5.82E-05 |
| Dennd4a | 1435975_at | E09.052.894_10 | 2.45E-05 | 0.004 | 5.65 | 6.03 | N | 2.84E-02 |
| Trappc6b | 1428177_at | E03.026.032_10 | 2.45E-05 | 0.003 | 9.15 | 9.38 | Y | 1.94E-01 |
| Nfkb1 | 1427705_a_at | E03.147.739_10 | 2.46E-05 | 0.003 | 9.58 | 9.19 | N | 7.35E-02 |
| Kpna1 | 1449504_at | E05.138.555_10 | 2.47E-05 | 0.003 | 6.08 | 5.42 | N | 3.17E-01 |
| 4933403F05Rik | 1434977_at | E18.060.777_10 | 2.48E-05 | 0.005 | 5.31 | 4.75 | Y | 7.83E-04 |
| Gigyf2 | 1451397_at | E01.135.010_10 | 2.49E-05 | 0.002 | 7.42 | 7.07 | Y | 7.30E-02 |
| Zmym6 | 1438685_at | E01.187.751_10 | 2.51E-05 | 0.003 | 5.55 | 5.22 | Y | 3.33E-01 |
| Sccpdh | 1426510_at | E01.169.742_10 | 2.52E-05 | 0.002 | 6.6 | 7.25 | Y | 4.48E-04 |
| Rap2b | 1439548_at | E09.052.894_10 | 2.53E-05 | 0 | 5.86 | 6.23 | N | 1.94E-01 |
| Mllt6 | 1433811_at | E11.101.632_10 | 2.54E-05 | 0.001 | 7.24 | 6.77 | Y | 5.61E-03 |
| Tor1b | 1448848_at | E02.019.239_10 | 2.54E-05 | 0.002 | 8.01 | 8.35 | Y | 1.08E-03 |
| Zik1 | 1433946_at | E12.105.658_10 | 2.56E-05 | 0.005 | 4.26 | 3.68 | N | 1.13E-01 |
| Cerk | 1434034_at | E15.042.560_10 | 2.59E-05 | 0.003 | 7.61 | 7.22 | Y | 7.07E-03 |
| Ppp1r11 | 1448565_at | E17.022.410_10 | 2.60E-05 | 0.006 | 8.82 | 8.23 | Y | 3.52E-03 |
| Chtf18 | 1452098_at | E05.107.059_10 | 2.60E-05 | 0.004 | 4.71 | 4.33 | N | 2.73E-01 |
| Tmpo | 1428976_at | E10.106.427_10 | 2.62E-05 | 0.002 | 8.81 | 8.29 | Y | 3.31E-01 |
| C230096C10Rik | 1436709_at | E04.141.582_10 | 2.62E-05 | 0.003 | 5.35 | 4.78 | N | 2.66E-01 |
| Pex7 | 1418988_at | E10.009.586_10 | 2.62E-05 | 0.003 | 8.3 | 7.96 | Y | 9.96E-03 |
| Tmem66 | 1424039_at | E08.023.645_10 | 2.62E-05 | 0.005 | 9.91 | 9.58 | N | 2.93E-01 |
| Gna13 | 1450656_at | E11.095.195_10 | 2.63E-05 | 0.004 | 7.47 | 7.01 | Y | 2.50E-04 |
| Fen1 | 1421731_a_at | E19.005.316_10 | 2.64E-05 | 0.005 | 7.93 | 7.39 | Y | 3.01E-02 |
| Brd3 | 1450902_at | E09.099.776_10 | 2.64E-05 | 0.001 | 6.45 | 6.73 | Y | 2.09E-01 |
| Tmem19 | 1416261_at | E10.118.344_10 | 2.65E-05 | 0.005 | 7.48 | 7.01 | Y | 1.43E-01 |
| Ndufaf1 | 1423711_at | E02.085.273_10 | 2.66E-05 | 0.004 | 5.7 | 6.2 | Y | 1.24E-03 |
| Dusp12 | 1420636_a_at | E09.034.366_10 | 2.67E-05 | 0.004 | 6.77 | 7.09 | Y | 2.83E-01 |
| St3gal4 | 1425668_a_at | E09.034.366_10 | 2.69E-05 | 0 | 8.38 | 7.76 | Y | 3.01E-01 |
| Tmed8 | 1428854_at | E06.001.969_10 | 2.73E-05 | 0.003 | 7.34 | 6.95 | N | 1.34E-01 |
| Hyou1 | 1423291_s_at | E09.034.366_10 | 2.75E-05 | 0.003 | 7.97 | 8.32 | Y | 1.82E-02 |
| 2310038H17Rik | 1449274_at | E01.060.215_10 | 2.76E-05 | 0.004 | 6.05 | 5.58 | Y | 6.27E-02 |
| 4930579G22Rik | 1429551_at | E05.126.262_10 | 2.79E-05 | 0.004 | 4 | 4.39 | N | 1.16E-03 |
| Rpl14 | 1422128_at | E09.116.239_10 | 2.81E-05 | 0.002 | 6.67 | 5.87 | Y | 5.12E-02 |
| Rabl5 | 1424575_at | E05.138.555_10 | 2.81E-05 | 0 | 6.85 | 6.26 | Y | 4.38E-02 |
| Syk | 1418262_at | E10.009.586_10 | 2.83E-05 | 0.005 | 6.57 | 6 | N | 3.46E-02 |
| Med4 | 1417844_at | E14.067.774_10 | 2.83E-05 | 0.003 | 7.93 | 7.6 | Y | 4.99E-03 |
| Nfya | 1422082_a_at | E05.138.555_10 | 2.87E-05 | 0.004 | 5.7 | 5.13 | N | 1.81E-01 |
| Klhdc3 | 1454747_a_at | E06.007.946_10 | 2.87E-05 | 0.004 | 9.16 | 8.87 | N | 3.31E-01 |
| Klf7 | 1419354_at | E01.016.840_10 | 2.88E-05 | 0.004 | 8 | 7.5 | Y | 2.43E-01 |
| Rer1 | 1460660_x_at | E04.156.084_10 | 2.88E-05 | 0.004 | 8.52 | 8.22 | Y | 7.64E-04 |
| Ubr2 | 1427163_at | E09.116.239_10 | 2.89E-05 | 0.004 | 6.32 | 6.76 | Y | 2.35E-01 |
| Dnajb6 | 1434035_at | E05.023.497_10 | 2.89E-05 | 0.005 | 8.3 | 7.77 | Y | 2.48E-03 |
| Ostm1 | 1428334_at | E10.009.586_10 | 2.89E-05 | 0.003 | 8.39 | 7.95 | Y | 4.83E-02 |
| Spast | 1454794_at | E17.068.056_10 | 2.91E-05 | 0.001 | 8.07 | 7.69 | Y | 4.46E-02 |
| 4930570C03Rik | 1450410_a_at | E15.101.379_10 | 2.92E-05 | 0.001 | 8.51 | 7.98 | Y | 3.21E-01 |
| Zbtb34 | 1436085_at | E06.032.288_10 | 2.92E-05 | 0.004 | 6.08 | 5.75 | N | 2.85E-01 |
| 3300001P08Rik | 1451485_at | E03.140.010_10 | 2.92E-05 | 0 | 8.23 | 7.87 | Y | 3.13E-01 |
| Cxcl16 | 1449195_s_at | E09.080.375_10 | 2.97E-05 | 0.001 | 8.18 | 7.53 | Y | 1.92E-01 |
| 4933433P14Rik | 1431334_a_at | E12.090.698_10 | 2.99E-05 | 0.002 | 7.33 | 6.87 | Y | 7.51E-02 |
| Insl6 | 1418346_at | E19.023.477_10 | 3.00E-05 | 0.006 | 6.07 | 5.18 | N | 6.76E-04 |
| Zfand2a | 1415940_at | E05.138.555_10 | 3.00E-05 | 0.002 | 8.87 | 8.32 | Y | 1.54E-02 |
| Slc1a3 | 1426341_at | E15.024.918_10 | 3.01E-05 | 0.001 | 3.97 | 4.71 | Y | 6.44E-02 |
| Slc35a4 | 1456013_x_at | E18.053.479_10 | 3.03E-05 | 0 | 7.35 | 6.66 | Y | 1.35E-05 |
| Eif4b | 1426379_at | E15.101.379_10 | 3.11E-05 | 0.002 | 9.17 | 8.69 | Y | 1.24E-01 |
| Ms4a4b | 1423467_at | E19.005.316_10 | 3.13E-05 | 0.001 | 2.62 | 3.1 | N | 3.45E-04 |
| Ing1 | 1448496_a_at | E06.007.946_10 | 3.13E-05 | 0.006 | 9.13 | 8.83 | N | 3.40E-01 |
| OTTMUSG00000010657 | 1451477_at | E07.002.028_10 | 3.13E-05 | 0.002 | 5.03 | 4.37 | Y | 5.49E-03 |
| Mterfd1 | 1454116_a_at | E13.056.787_10 | 3.14E-05 | 0.004 | 8 | 7.66 | Y | 6.92E-04 |
| Pphln1 | 1434822_at | E15.101.153_10 | 3.15E-05 | 0.004 | 7.28 | 6.91 | Y | 1.42E-01 |
| Perld1 | 1435037_at | E11.101.632_10 | 3.16E-05 | 0.006 | 4.57 | 4.17 | Y | 3.22E-03 |
| Csde1 | 1427396_a_at | E03.100.082_10 | 3.18E-05 | 0.003 | 11.66 | 11.42 | Y | 9.13E-03 |
| Wdr5 | 1416581_at | E02.015.712_10 | 3.19E-05 | 0.002 | 7.82 | 7.59 | Y | 4.39E-02 |
| AI462493 | 1455152_at | E19.005.316_10 | 3.19E-05 | 0.005 | 7.48 | 7.21 | Y | 4.91E-02 |
| Dock2 | 1422808_s_at | E06.032.288_10 | 3.20E-05 | 0.004 | 6.63 | 5.96 | N | 2.37E-01 |
| Atxn10 | 1450666_s_at | E15.045.423_10 | 3.20E-05 | 0.005 | 5.41 | 5.05 | Y | 7.30E-02 |
| Stat2 | 1421911_at | E10.118.344_10 | 3.21E-05 | 0.002 | 4.8 | 5.54 | Y | 1.27E-02 |
| Hbxip | 1436152_a_at | E03.100.082_10 | 3.24E-05 | 0.006 | 9.44 | 8.86 | Y | 1.09E-06 |
| Tsn | 1448516_at | E01.086.274_10 | 3.27E-05 | 0.001 | 7.44 | 7.05 | Y | 1.71E-01 |
| Ndrg1 | 1450976_at | E09.034.366_10 | 3.30E-05 | 0.006 | 9.75 | 8.9 | N | 2.98E-01 |
| Arl5b | 1437884_at | E02.019.239_10 | 3.31E-05 | 0.002 | 5.87 | 5.32 | Y | 1.28E-03 |
| Gpr137b | 1450881_s_at | E13.014.119_10 | 3.31E-05 | 0.004 | 8.68 | 7.97 | Y | 3.92E-04 |
| Zfp187 | 1457285_at | E13.001.439_10 | 3.33E-05 | 0.002 | 7.75 | 7.45 | Y | 1.33E-02 |
| Rnf6 | 1427898_at | E05.138.555_10 | 3.34E-05 | 0.003 | 8.82 | 8.44 | N | 1.40E-02 |
| Phf3 | 1429000_at | E01.033.082_10 | 3.35E-05 | 0.005 | 5.57 | 5 | N | 2.47E-02 |
| Osgepl1 | 1435127_a_at | E01.060.215_10 | 3.35E-05 | 0.006 | 6.02 | 5.38 | Y | 3.48E-02 |
| Tnip2 | 1419488_at | E05.107.059_10 | 3.39E-05 | 0.003 | 5.26 | 5.54 | N | 2.64E-01 |
| Tbc1d22a | 1423776_s_at | E15.073.732_10 | 3.40E-05 | 0.004 | 7.85 | 7.58 | Y | 1.91E-02 |
| Jtv1 | 1424151_at | E05.138.555_10 | 3.42E-05 | 0.004 | 9.1 | 8.72 | Y | 9.03E-02 |
| 2900073G15Rik | 1450013_at | E17.068.056_10 | 3.44E-05 | 0.002 | 7.18 | 6.85 | N | 1.89E-03 |
| Mrps25 | 1418716_at | E06.092.041_10 | 3.45E-05 | 0.006 | 5.69 | 5.25 | Y | 2.95E-03 |
| Nrd1 | 1424391_at | E10.118.344_10 | 3.47E-05 | 0.005 | 8.84 | 8.58 | Y | 3.04E-01 |
| Zfp294 | 1452612_at | E01.001.113_10 | 3.48E-05 | 0.006 | 7.61 | 7.33 | N | 2.10E-01 |
| Slc13a2 | 1418857_at | E10.118.344_10 | 3.49E-05 | 0.003 | 4.03 | 4.22 | N | 3.39E-01 |
| 1110014N23Rik | 1454825_at | E19.005.316_10 | 3.49E-05 | 0.006 | 6.11 | 6.37 | Y | 6.96E-04 |
| Map2k4 | 1451982_at | E11.101.632_10 | 3.50E-05 | 0.005 | 8.69 | 8.34 | N | 2.93E-01 |
| Pign | 1432115_a_at | E01.094.380_10 | 3.53E-05 | 0.006 | 5.96 | 6.63 | Y | 7.64E-04 |
| D9Ertd402e | 1455118_at | E09.102.375_10 | 3.56E-05 | 0.006 | 5.72 | 5.38 | Y | 7.96E-03 |
| Irak4 | 1451749_at | E15.101.153_10 | 3.59E-05 | 0.006 | 4.57 | 5.05 | N | 1.79E-01 |
| Sbno1 | 1455026_at | E05.126.262_10 | 3.59E-05 | 0.002 | 6.49 | 5.9 | Y | 8.26E-03 |
| 4921506J03Rik | 1428127_at | E10.118.344_10 | 3.63E-05 | 0.003 | 8.04 | 8.42 | Y | 3.12E-01 |
| Med1 | 1421907_at | E11.101.632_10 | 3.63E-05 | 0.004 | 7.68 | 7.22 | Y | 2.50E-04 |
| Arhgap25 | 1437072_at | E06.032.288_10 | 3.66E-05 | 0.002 | 5.42 | 4.88 | N | 3.04E-01 |
| Kif1c | 1424746_at | E11.070.437_10 | 3.68E-05 | 0.001 | 8.99 | 8.41 | Y | 2.28E-03 |
| Glt8d3 | 1434876_at | E02.112.734_10 | 3.71E-05 | 0.006 | 7.32 | 7.75 | Y | 2.85E-01 |
| BC025546 | 1451273_x_at | E08.129.275_10 | 3.77E-05 | 0.005 | 4.99 | 4.55 | Y | 1.71E-01 |
| Ppp2r5c | 1427003_at | E14.019.954_10 | 3.90E-05 | 0.002 | 6.87 | 7.16 | N | 2.74E-01 |
| Ubac2 | 1423928_at | E14.116.830_10 | 3.91E-05 | 0.006 | 7.09 | 6.83 | Y | 9.83E-03 |
| Pcm1 | 1436908_at | E08.038.758_10 | 3.91E-05 | 0.005 | 5.82 | 5.13 | Y | 2.47E-01 |
| Phip | 1425721_at | E03.140.010_10 | 3.92E-05 | 0.004 | 4.33 | 4.88 | N | 2.20E-01 |
| Aspm | 1422814_at | E09.099.776_10 | 3.96E-05 | 0.001 | 6.87 | 7.39 | N | 2.55E-01 |
| Scp2 | 1449686_s_at | E04.106.454_10 | 3.96E-05 | 0.005 | 9.41 | 9.74 | N | 1.82E-02 |
| Pigk | 1428488_at | E03.158.424_10 | 3.97E-05 | 0.006 | 7.46 | 7.88 | Y | 5.94E-02 |
| Zadh1 | 1451744_a_at | E12.090.698_10 | 4.01E-05 | 0.003 | 6.26 | 5.66 | Y | 8.23E-02 |
| LOC100048105 | 1420494_x_at | E05.126.262_10 | 4.02E-05 | 0.005 | 11.66 | 11.42 | Y | 1.44E-03 |
| Gapdh | 1418625_s_at | E06.131.131_10 | 4.04E-05 | 0.002 | 13.59 | 13.25 | Y | 3.22E-03 |
| Eif2s3x | 1421895_at | E06.032.288_10 | 4.06E-05 | 0.005 | 7.69 | 8.41 | N | 3.09E-01 |
| Ppp2r4 | 1448138_at | E02.019.239_10 | 4.07E-05 | 0.006 | 8.21 | 8.5 | N | 2.92E-02 |
| Bik | 1449836_x_at | E15.069.357_10 | 4.11E-05 | 0 | 5.04 | 4.58 | Y | 1.22E-02 |
| Trim44 | 1431932_s_at | E05.138.555_10 | 4.12E-05 | 0.002 | 6.17 | 5.7 | N | 2.83E-01 |
| Creg1 | 1415947_at | E01.160.825_10 | 4.13E-05 | 0.004 | 8.42 | 7.86 | N | 2.90E-02 |
| Msrb2 | 1424433_at | E02.019.239_10 | 4.19E-05 | 0.006 | 5.32 | 4.66 | N | 3.91E-03 |
| Hexim1 | 1425937_a_at | E15.101.379_10 | 4.19E-05 | 0.003 | 8.35 | 8.61 | Y | 7.30E-02 |
| Rbm27 | 1456058_at | E18.056.581_10 | 4.21E-05 | 0.004 | 7.5 | 7.18 | Y | 1.22E-02 |
| Trove2 | 1436535_at | E01.144.066_10 | 4.29E-05 | 0.002 | 5.9 | 6.42 | Y | 1.59E-04 |
| Pdxp | 1417890_at | E15.013.193_10 | 4.31E-05 | 0.003 | 5.29 | 4.83 | Y | 2.15E-01 |
| Myo10 | 1450650_at | E15.024.918_10 | 4.31E-05 | 0.006 | 7.96 | 7.43 | Y | 1.87E-01 |
| Higd2a | 1418002_at | E13.037.637_10 | 4.35E-05 | 0.005 | 10.29 | 9.9 | Y | 2.72E-03 |
| Capzb | 1424168_a_at | E04.141.582_10 | 4.35E-05 | 0.006 | 10.22 | 10.78 | Y | 2.95E-03 |
| Abcb8 | 1423713_at | E05.023.497_10 | 4.40E-05 | 0.002 | 5.56 | 5.17 | Y | 7.29E-02 |
| Yipf5 | 1449142_a_at | E07.133.429_10 | 4.41E-05 | 0.003 | 9.67 | 9.38 | N | 2.44E-01 |
| Eps8 | 1422823_at | E03.140.010_10 | 4.45E-05 | 0.006 | 8.44 | 9.19 | N | 3.31E-01 |
| Ptgfrn | 1434891_at | E03.100.082_10 | 4.48E-05 | 0.004 | 9.41 | 8.99 | Y | 1.55E-02 |
| Nsf | 1422456_at | E01.008.129_10 | 4.52E-05 | 0.005 | 8.3 | 8 | Y | 2.00E-01 |
| Ate1 | 1435173_at | E07.079.411_10 | 4.59E-05 | 0.003 | 7.37 | 6.95 | Y | 1.12E-01 |
| Casp4 | 1449591_at | E06.001.969_10 | 4.62E-05 | 0.006 | 8.23 | 7.46 | N | 2.70E-01 |
| Zdhhc4 | 1432136_s_at | E05.138.555_10 | 4.67E-05 | 0.004 | 7.83 | 7.51 | Y | 1.49E-02 |
| Tslp | 1450004_at | E05.138.555_10 | 4.72E-05 | 0.002 | 5.1 | 7.21 | N | 2.95E-01 |
| Gas2l3 | 1453416_at | E10.106.427_10 | 4.87E-05 | 0.006 | 6.6 | 5.94 | Y | 2.57E-01 |
| H2afx | 1416746_at | E09.052.894_10 | 5.08E-05 | 0.004 | 9.08 | 8.64 | N | 2.93E-01 |
| BC013529 | 1424026_s_at | E10.009.586_10 | 5.11E-05 | 0.004 | 7.95 | 7.65 | Y | 1.43E-02 |
| Taf8 | 1416451_s_at | E17.056.536_10 | 5.13E-05 | 0.004 | 5.63 | 5.2 | Y | 2.38E-03 |
| Gpatch1 | 1419013_at | E02.122.101_10 | 5.15E-05 | 0.003 | 6.4 | 6.68 | N | 3.05E-01 |
| Sel1l | 1425188_s_at | E03.026.032_10 | 5.16E-05 | 0.002 | 6.55 | 7.01 | Y | 2.06E-01 |
| Mtap2 | 1434194_at | E10.102.359_10 | 5.16E-05 | 0.005 | 4.61 | 3.63 | N | 2.94E-01 |
| Cd80 | 1451950_a_at | E16.047.738_10 | 5.18E-05 | 0.003 | 5.35 | 6.15 | N | 6.99E-03 |
| Sugt1 | 1426425_at | E14.094.324_10 | 5.20E-05 | 0.004 | 9.99 | 9.67 | N | 1.22E-01 |
| Ppih | 1429832_at | E05.044.682_10 | 5.32E-05 | 0.006 | 6.77 | 6.23 | N | 3.13E-01 |
| BC024659 | 1433986_at | E13.001.439_10 | 5.33E-05 | 0.005 | 7.44 | 6.97 | Y | 3.21E-03 |
| Tob2 | 1448667_x_at | E02.015.712_10 | 5.41E-05 | 0.005 | 7.77 | 8.04 | N | 2.61E-01 |
| 1810031K17Rik | 1427879_at | E01.073.342_10 | 5.42E-05 | 0.001 | 8.54 | 8.16 | Y | 2.68E-01 |
| Pole4 | 1423371_at | E06.049.290_10 | 5.50E-05 | 0.001 | 6.35 | 5.89 | Y | 6.43E-03 |
| Ero1l | 1419030_at | E09.034.366_10 | 5.53E-05 | 0.005 | 9.55 | 8.46 | N | 1.43E-02 |
| Rab40c | 1424332_at | E06.032.288_10 | 5.54E-05 | 0.005 | 4.83 | 5.44 | Y | 1.49E-01 |
| Gstm2 | 1416411_at | E03.127.496_10 | 5.72E-05 | 0.003 | 6.38 | 7.19 | Y | 2.21E-02 |
| Pigq | 1415946_at | E17.022.410_10 | 5.74E-05 | 0.004 | 7.73 | 7.33 | Y | 1.91E-02 |
| Mllt6 | 1425360_at | E02.048.324_10 | 5.75E-05 | 0.004 | 4.63 | 4.35 | N | 3.12E-01 |
| Ptger2 | 1449310_at | E14.019.954_10 | 5.80E-05 | 0.002 | 6.13 | 5.24 | N | 9.67E-02 |
| Rrs1 | 1416998_at | E01.001.113_10 | 5.80E-05 | 0.003 | 9.31 | 8.77 | Y | 2.46E-04 |
| Med20 | 1416826_a_at | E17.068.056_10 | 5.81E-05 | 0.002 | 8.16 | 7.54 | Y | 1.26E-05 |
| Smndc1 | 1452999_at | E19.055.858_10 | 5.91E-05 | 0.001 | 8.12 | 7.76 | N | 3.12E-04 |
| Exosc6 | 1435544_at | E08.129.275_10 | 5.91E-05 | 0.006 | 8.7 | 8.35 | N | 2.55E-01 |
| Gab1 | 1417694_at | E08.038.758_10 | 6.05E-05 | 0.002 | 6.73 | 6.25 | Y | 7.33E-03 |
| Ncoa4 | 1450006_at | E03.019.137_10 | 6.11E-05 | 0.001 | 8.32 | 8.86 | Y | 2.85E-01 |
| Stx17 | 1428917_at | E04.040.685_10 | 6.13E-05 | 0.006 | 7.01 | 6.38 | Y | 1.59E-04 |
| AI662250 | 1433687_at | E02.104.443_10 | 6.14E-05 | 0.005 | 5.37 | 5.69 | Y | 3.12E-01 |
| Caprin1 | 1416461_at | E02.112.734_10 | 6.17E-05 | 0.003 | 11.02 | 10.74 | Y | 2.48E-01 |
| Tep1 | 1418196_at | E14.041.725_10 | 6.18E-05 | 0.005 | 7.59 | 7.15 | N | 1.92E-03 |
| Commd9 | 1454781_x_at | E02.078.067_10 | 6.22E-05 | 0.006 | 5.8 | 5.42 | Y | 9.57E-03 |
| Gne | 1455583_at | E04.043.559_10 | 6.28E-05 | 0.003 | 5.06 | 4.72 | Y | 9.92E-02 |
| Higd1a | 1416481_s_at | E09.102.375_10 | 6.38E-05 | 0.004 | 6.34 | 5.62 | Y | 3.94E-03 |
| Ttc32 | 1452972_at | E12.005.190_10 | 6.38E-05 | 0.006 | 6.57 | 7.08 | Y | 1.28E-03 |
| 2310076L09Rik | 1441915_s_at | E17.056.536_10 | 6.41E-05 | 0.005 | 4.93 | 5.22 | N | 4.74E-02 |
| Fbln1 | 1439688_at | E02.092.755_10 | 6.42E-05 | 0.005 | 4.77 | 4.51 | N | 1.70E-02 |
| Atp6v0e | 1416328_a_at | E01.086.274_10 | 6.42E-05 | 0.004 | 10.63 | 10.34 | Y | 1.30E-01 |
| Coro1a | 1455269_a_at | E07.133.429_10 | 6.44E-05 | 0.006 | 9 | 8.32 | N | 1.08E-01 |
| Sdc4 | 1417654_at | E02.172.454_10 | 6.51E-05 | 0.006 | 9.43 | 9.95 | Y | 5.92E-03 |
| Serpina3g | 1424923_at | E14.019.954_10 | 6.61E-05 | 0.004 | 6.29 | 5.5 | Y | 1.65E-01 |
| Tmed5 | 1424573_at | E05.103.695_10 | 6.66E-05 | 0.005 | 8.49 | 8 | Y | 4.03E-04 |
| Orc2l | 1418225_at | E09.099.776_10 | 6.70E-05 | 0.005 | 6.51 | 6.83 | N | 2.28E-01 |
| Pik3cd | 1453281_at | E06.032.288_10 | 6.97E-05 | 0.006 | 5.94 | 5.5 | N | 3.03E-01 |
| 2010012O05Rik | 1419299_at | E19.055.858_10 | 7.05E-05 | 0.005 | 6.85 | 6.28 | Y | 2.80E-01 |
| Hdac7a | 1420812_at | E15.101.379_10 | 7.15E-05 | 0.006 | 6.14 | 5.87 | N | 4.42E-02 |
| H47 | 1435735_x_at | E07.079.411_10 | 7.33E-05 | 0.003 | 8.9 | 8.44 | Y | 1.03E-02 |
| Tardbp | 1436318_at | E01.187.751_10 | 7.41E-05 | 0.005 | 6.37 | 5.98 | N | 2.93E-01 |
| Cct2 | 1433534_a_at | E10.118.344_10 | 7.42E-05 | 0.005 | 9.49 | 8.99 | Y | 1.85E-03 |
| Snrpb | 1419260_a_at | E05.103.695_10 | 7.43E-05 | 0.006 | 10.17 | 9.96 | Y | 2.97E-01 |
| Trip13 | 1429294_at | E05.044.682_10 | 7.46E-05 | 0.006 | 7.27 | 6.77 | N | 2.10E-01 |
| Yeats4 | 1423105_a_at | E10.118.344_10 | 7.54E-05 | 0.001 | 8.88 | 8.51 | Y | 2.24E-01 |
| Dnajc1 | 1420500_at | E06.032.288_10 | 7.55E-05 | 0.003 | 7.71 | 7.39 | Y | 1.10E-01 |
| Usp46 | 1435325_at | E05.044.682_10 | 7.65E-05 | 0.005 | 7.53 | 7.09 | Y | 9.10E-02 |
| Sntb2 | 1420371_at | E08.112.069_10 | 7.77E-05 | 0.005 | 5.46 | 5.19 | N | 2.93E-02 |
| Pgm3 | 1428228_at | E09.052.894_10 | 7.78E-05 | 0.004 | 6.07 | 6.5 | Y | 1.14E-01 |
| Chd1l | 1449415_at | E09.094.015_10 | 7.92E-05 | 0.004 | 6.78 | 7.13 | N | 2.79E-01 |
| Man1a2 | 1420977_at | E07.136.725_10 | 7.93E-05 | 0.005 | 7.64 | 7.98 | N | 7.36E-02 |
| Frs2 | 1437891_at | E10.118.344_10 | 8.17E-05 | 0.006 | 7.94 | 7.53 | N | 1.86E-01 |
| Gpatch2 | 1445189_at | E06.001.969_10 | 8.26E-05 | 0.006 | 5.26 | 5.59 | N | 4.41E-02 |
| Med13 | 1453160_at | E10.106.427_10 | 8.28E-05 | 0.005 | 7.97 | 7.61 | N | 2.03E-02 |
| Cks1b | 1448441_at | E05.107.059_10 | 8.61E-05 | 0.005 | 9.68 | 9.19 | Y | 2.68E-01 |
| Nfib | 1427680_a_at | E10.102.359_10 | 8.63E-05 | 0.004 | 8.58 | 7.96 | Y | 1.27E-01 |
| Abcb9 | 1416264_at | E06.001.969_10 | 8.80E-05 | 0.006 | 5.51 | 5.86 | N | 1.97E-01 |
| Pigv | 1437663_at | E18.077.468_10 | 8.85E-05 | 0.006 | 5.38 | 5.64 | Y | 1.43E-01 |
| Rasl11b | 1423854_a_at | E05.092.044_10 | 8.93E-05 | 0.004 | 6.08 | 5.51 | Y | 8.43E-03 |
| 6330416G13Rik | 1426316_at | E06.032.288_10 | 9.22E-05 | 0.006 | 5.99 | 5.58 | N | 2.07E-01 |
| Parn | 1437334_x_at | E16.018.064_10 | 9.38E-05 | 0.004 | 7.32 | 7.02 | Y | 7.97E-02 |
| Klhl12 | 1435786_at | E01.144.066_10 | 9.95E-05 | 0.006 | 7.19 | 6.84 | Y | 3.17E-01 |
| Pctk3 | 1449151_at | E01.125.571_10 | 9.99E-05 | 0.005 | 5.94 | 5.14 | N | 1.14E-01 |
| BC055107 | 1434202_a_at | E16.033.089_10 | 1.01E-04 | 0.005 | 3.35 | 2.83 | N | 3.05E-01 |
| Fbxo3 | 1423490_at | E02.092.755_10 | 1.01E-04 | 0.006 | 7.94 | 7.51 | Y | 2.40E-01 |
| D430028G21Rik | 1451176_at | E01.169.742_10 | 1.11E-04 | 0.002 | 6.81 | 6.53 | N | 2.74E-01 |
| Ccdc117 | 1460399_at | E02.157.973_10 | 1.01E-04 | 0.006 | 5.44 | 5.78 | N | 2.75E-01 |
| Snap29 | 1423356_at | E16.018.064_10 | 1.01E-04 | 0.004 | 6.22 | 5.84 | Y | 5.37E-02 |
| Wdfy1 | 1437358_at | E01.135.010_10 | 1.18E-04 | 0.006 | 7.14 | 6.72 | Y | 2.05E-01 |
| Safb2 | 1427988_s_at | E05.138.555_10 | 1.21E-04 | 0.006 | 7.1 | 7.4 | Y | 2.89E-01 |
| Bicd2 | 1450732_a_at | E13.037.637_10 | 1.25E-04 | 0.006 | 8.86 | 8.34 | Y | 1.54E-02 |
| Adra2a | 1433600_at | E03.081.606_10 | 1.27E-04 | 0.006 | 5.39 | 6.25 | Y | 2.00E-01 |
| Ctnnb1 | 1450008_a_at | E03.140.010_10 | 1.28E-04 | 0.006 | 10.07 | 10.53 | N | 4.42E-02 |
| Dusp10 | 1417163_at | E05.126.262_10 | 1.32E-04 | 0.004 | 4.12 | 3.71 | N | 8.51E-02 |
| Acss1 | 1416617_at | E06.001.969_10 | 1.36E-04 | 0.006 | 4.93 | 4.33 | Y | 1.89E-01 |
| Slc5a5 | 1436239_at | E08.054.070_10 | 1.44E-04 | 0.004 | 3.44 | 3.84 | N | 1.31E-02 |
| Ctnna1 | 1437807_x_at | E18.053.479_10 | 1.44E-04 | 0 | 11.29 | 10.69 | Y | 7.82E-05 |
| Oxct1 | 1436750_a_at | E15.024.918_10 | 1.78E-04 | 0.005 | 8.24 | 7.57 | Y | 1.97E-02 |
| Sec61a1 | 1416190_a_at | E05.138.555_10 | 1.95E-04 | 0.006 | 8.11 | 7.49 | N | 1.49E-01 |
| Crnkl1 | 1420850_at | E02.137.900_10 | 2.01E-04 | 0.002 | 7.72 | 7.35 | Y | 4.87E-02 |
| Spast | 1460400_at | E03.140.010_10 | 2.15E-04 | 0.005 | 6.55 | 6.85 | N | 2.11E-01 |
| Trpm7 | 1416800_at | E02.125.375_10 | 3.80E-04 | 0.003 | 7.84 | 7.4 | Y | 1.91E-01 |

**Table S2**

**Counts of candidate eQTL identified at a maximum 10% False Discovery Rate in Skin, Papillomas, and Carcinomas.**

Columns identify counts of *cis*- or *trans*-eQTL where mean values are higher in FVB/N homozygous alleles (FF) or FVB/N Spret/Ei heterozygotes (FS). Tail data taken from [38]. Papilloma data contains 37 unique genotypes; tail and carcinoma contain 71 and 55 respectively. Three refseq-annotated probes with significant papilloma eQTL do not have alignments on the mouse reference genome and therefore could not be assigned *cis* or *trans* status: *Noc2l1* (1425288_at) , *Eraf* (1449077_at), *Samd11* (1424323_at). Note that *cis* control was defined as locus less than 30 Mb. away from gene; as stated in Methods, changing this window to 20 or 40 Mb. did not affect the conclusions.

|  | **TOTAL** | **CIS (same Chromosome)** | | **TRANS** | |
| --- | --- | --- | --- | --- | --- |
|  |  | Higher FF (%) | Higher FS (%) | Higher FF (%) | Higher FS (%) |
| *Tail* | 7414 | 2622 (78) | 733 (22) | 1592 (39) | 2467 (61) |
| *Papilloma* | 3408 | 1658 (77) | 488 (23) | 642 (51) | 620 (49) |
| *Carcinoma* | 912 | 576 (79) | 155 (21) | 111 (61) | 70 (39) |

A locus with no true effect on gene expression could appear to be a *cis*-acting eQTL if polymorphisms in one parental strain selectively interfere with probe hybridization. This phenomenon will not affect gene co-expression measurements, but can in principle lead to false positive *cis*-eQTL results. A bias in favor of lower SPRET/Ei expression for *cis*-eQTL suggested that some of these candidate *cis*-eQTL may be affected by probe hybridization bias.

Removal of probesets with known or predicted sequence variation in probes has been proposed to address this issue [39]. We investigated this issue for *Lgr5*, as we previously reported it has a *cis*-acting eQTL in skin [38]. We aligned the relevant microarray probes against the SPRET/Ei genome and noted that although there are SPRET/Ei SNPs in two of eleven probe binding regions, the eQTL effect is seen in all probes. Importantly, probes containing SNPs do not overlap with the other probes in the probeset, and quantitative PCR analysis confirmed differential expression of these genes in parental strains (Figure S6 in Additional File 1 and [38]). We also identified genes with higher expression in heterozygous alleles despite SPRET/Ei-specific SNPs in probe-binding regions (data not shown). We therefore advocate careful validation of putatively significant *cis*-eQTL after biologically interesting candidates have been identified rather than preemptive removal based on predicted SNP effects.

**Table S3**

Genes altered in carcinomas more than 2 standard deviations more than the mean fold-change. Values are the mean difference in log_2_ expression between tumor and tail skin, so a value of 2 represents four-fold change, while a value of 3 represents 8-fold change. Tumor microarrays were separately re-normalized from original CEL files with tail microarrays to allow for meaningful direct comparison of mRNA expression values.

| **Gene** | **probe** | **Foldchange** |
| --- | --- | --- |
| Krt2 | 1427154_at | -9.80 |
| Krt84 | 1451551_at | -8.32 |
| Krtap16-8 | 1425237_at | -7.64 |
| 2310057N15Rik | 1421575_at | -7.64 |
| Krtap6-1 | 1451859_at | -7.63 |
| 2310034C09Rik | 1449986_at | -7.03 |
| Krtap13 | 1420358_at | -6.94 |
| Hrnr | 1451613_at | -6.88 |
| Krtap16-10 | 1427549_s_at | -6.67 |
| Krtap6-2 | 1449919_at | -6.58 |
| Cyp2b19 | 1419731_at | -6.38 |
| Serpinb12 | 1429297_at | -6.38 |
| Krtap3-3 | 1452957_at | -6.29 |
| Lce1c | 1453218_at | -6.21 |
| Them5 | 1453801_at | -6.17 |
| Krtap16-5 | 1425430_at | -6.15 |
| Lor | 1448745_s_at | -6.06 |
| Serpinb3c | 1422939_at | -5.87 |
| Serpinb3c | 1422940_x_at | -5.84 |
| Lce1d | 1420332_x_at | -5.84 |
| Krtap16-10 | 1425431_at | -5.83 |
| Krtap7-1 | 1432540_at | -5.80 |
| AY026312 | 1420415_at | -5.78 |
| Krtap11-1 | 1439100_s_at | -5.78 |
| Krtap13-1 | 1428007_at | -5.77 |
| Lce1a2 | 1420350_at | -5.68 |
| Lce1b | 1419409_at | -5.63 |
| Expi | 1417160_s_at | -5.57 |
| Krtap17-1 | 1453523_at | -5.53 |
| Krt33a | 1449387_at | -5.51 |
| Serpina12 | 1421092_at | -5.43 |
| Lce1a1 | 1420676_at | -5.40 |
| Panx3 | 1456073_s_at | -5.39 |
| Olah | 1451510_s_at | -5.36 |
| Asah3 | 1439183_at | -5.32 |
| Them5 | 1431211_s_at | -5.32 |
| Lce1i | 1420741_x_at | -5.31 |
| Krtap8-2 | 1421689_at | -5.30 |
| Krt25 | 1418173_at | -5.28 |
| Lce1l | 1418855_at | -5.23 |
| Adh1 | 1416225_at | -5.21 |
| Coch | 1423285_at | -5.19 |
| Krt34 | 1418742_at | -5.19 |
| A030010K20Rik | 1441307_at | -5.18 |
| Lce1h | 1449959_x_at | -5.15 |
| 1110054P19Rik | 1431650_at | -5.15 |
| Cyp2f2 | 1448792_a_at | -5.14 |
| Slurp1 | 1420562_at | -5.13 |
| Krtap14 | 1419707_at | -5.12 |
| Lce1f | 1420550_at | -5.10 |
| Lce1a1 | 1420677_x_at | -5.09 |
| 5430421N21Rik | 1427365_at | -5.09 |
| Lass4 | 1417780_at | -5.07 |
| Dsg1a | 1458000_at | -5.06 |
| Ear5 | 1450616_at | -5.05 |
| Fa2h | 1426960_a_at | -5.03 |
| Krt10 | 1452166_a_at | -5.01 |
| Rdhe2 | 1440523_at | -5.00 |
| Defb6 | 1421807_at | -4.95 |
| 2310043L02Rik | 1449560_at | -4.92 |
| Sptlc3 | 1439492_at | -4.88 |
| OTTMUSG00000002196 | 1451774_at | -4.87 |
| Kprp | 1428980_at | -4.87 |
| Krt31 | 1421589_at | -4.81 |
| Tmem56 | 1434553_at | -4.80 |
| Psors1c2 | 1420467_at | -4.79 |
| Lass4 | 1417782_at | -4.79 |
| Krtap12-1 | 1450536_s_at | -4.75 |
| Dsc1 | 1421460_at | -4.71 |
| Mbp | 1433532_a_at | -4.71 |
| Krtap4-7 | 1430669_at | -4.70 |
| Orm1 | 1451054_at | -4.69 |
| Wfdc12 | 1449191_at | -4.68 |
| Krtap16-9 | 1427801_at | -4.67 |
| Krtap5-2 | 1420452_at | -4.66 |
| Krtap16-1 | 1425655_at | -4.66 |
| Mtap2 | 1434194_at | -4.65 |
| Rdh9 | 1427963_s_at | -4.64 |
| Klk5 | 1429230_at | -4.63 |
| Gsdm1 | 1457777_at | -4.62 |
| Elovl3 | 1420722_at | -4.61 |
| 2310040M23Rik | 1449963_at | -4.61 |
| RP23-212C14.7 | 1430509_at | -4.60 |
| Gm11563 | 1442089_at | -4.52 |
| Oas1f | 1442389_at | -4.48 |
| Mbp | 1456228_x_at | -4.44 |
| 2310002B14Rik | 1429957_at | -4.42 |
| Lass4 | 1417781_at | -4.42 |
| Cyp1a1 | 1422217_a_at | -4.38 |
| Myl2 | 1448394_at | -4.33 |
| Aldh3a1 | 1418752_at | -4.33 |
| Selenbp1 | 1450699_at | -4.26 |
| Slc46a2 | 1423476_at | -4.25 |
| Serpinb3a | 1437517_x_at | -4.22 |
| Mpz | 1423253_at | -4.21 |
| A030004J04Rik | 1444061_at | -4.20 |
| Krt79 | 1427352_at | -4.19 |
| Pof1b | 1427492_at | -4.14 |
| Psapl1 | 1440186_s_at | -4.13 |
| Atp12a | 1449475_at | -4.13 |
| Vsig8 | 1436671_at | -4.09 |
| Defb3 | 1421806_at | -4.08 |
| Asah3 | 1450825_at | -4.08 |
| Cds1 | 1428680_at | -4.06 |
| Krt27 | 1449378_at | -4.03 |
| Cidea | 1417956_at | -4.02 |
| Il1f5 | 1421370_a_at | -4.02 |
| Krt35 | 1420409_at | -4.01 |
| Arl4a | 1435092_at | -3.98 |
| Tyrp1 | 1415861_at | -3.96 |
| Krtap16-4 | 1426203_at | -3.94 |
| Ly6g6c | 1422749_at | -3.93 |
| Mug1 | 1448854_s_at | -3.93 |
| Cdsn | 1435191_at | -3.91 |
| Elovl7 | 1440312_at | -3.90 |
| Dct | 1418028_at | -3.89 |
| Dnase1l2 | 1450936_a_at | -3.89 |
| 1110033F04Rik | 1421494_at | -3.88 |
| Acoxl | 1460470_at | -3.87 |
| 2810432L12Rik | 1423679_at | -3.86 |
| Elovl7 | 1440354_at | -3.85 |
| Elovl4 | 1424306_at | -3.83 |
| Apod | 1416371_at | -3.79 |
| Elovl6 | 1417404_at | -3.79 |
| Lce1m | 1429565_s_at | -3.77 |
| Odz2 | 1457273_at | -3.77 |
| Tcfl5 | 1456515_s_at | -3.77 |
| Sbsn | 1459898_at | -3.76 |
| Lce1g | 1421316_at | -3.75 |
| Spink12 | 1453503_at | -3.75 |
| Adh7 | 1421058_at | -3.74 |
| Tnmd | 1417979_at | -3.70 |
| Kcna1 | 1455785_at | -3.70 |
| Elovl6 | 1417403_at | -3.69 |
| Krt71 | 1448457_at | -3.69 |
| BC026374 | 1451615_at | -3.68 |
| Gsdm1 | 1423634_at | -3.68 |
| Cst6 | 1427910_at | -3.68 |
| Ace2 | 1425102_a_at | -3.67 |
| Igfbp5 | 1452114_s_at | -3.67 |
| Camta1 | 1433971_at | -3.66 |
| Selenbp1 | 1417580_s_at | -3.66 |
| Sec14l4 | 1424676_s_at | -3.65 |
| Rdh1 | 1421702_at | -3.64 |
| Rptn | 1420431_at | -3.64 |
| Ace2 | 1425103_at | -3.64 |
| Tmem45a | 1422587_at | -3.64 |
| Psapl1 | 1429457_at | -3.62 |
| Krt73 | 1436557_at | -3.62 |
| Plcxd1 | 1437842_at | -3.61 |
| Hpgd | 1419905_s_at | -3.61 |
| Mgll | 1450391_a_at | -3.61 |
| Fmod | 1415939_at | -3.59 |
| Elovl7 | 1441091_at | -3.59 |
| Olah | 1424855_at | -3.54 |
| Rora | 1420583_a_at | -3.53 |
| Elmod1 | 1434083_a_at | -3.53 |
| C1qtnf4 | 1417050_at | -3.51 |
| Ttpa | 1427284_a_at | -3.50 |
| Eppk1 | 1427537_at | -3.49 |
| Mgll | 1426785_s_at | -3.49 |
| Tmem20 | 1435452_at | -3.48 |
| Krtap5-1 | 1450539_at | -3.48 |
| Comp | 1419527_at | -3.45 |
| Cttnbp2 | 1435435_at | -3.43 |
| Lipm | 1430551_s_at | -3.42 |
| Mbp | 1419646_a_at | -3.41 |
| Elovl7 | 1424097_at | -3.40 |
| Defb1 | 1419491_at | -3.40 |
| Hsd3b2 | 1460232_s_at | -3.39 |
| Bbox1 | 1419618_at | -3.39 |
| Abca8a | 1427371_at | -3.38 |
| Mfsd2 | 1428223_at | -3.35 |
| Tnnc1 | 1418370_at | -3.35 |
| Scd1 | 1415964_at | -3.35 |
| EG545947 | 1425032_at | -3.34 |
| Elovl7 | 1424098_at | -3.34 |
| Gp1bb | 1422977_at | -3.34 |
| Snap91 | 1416688_at | -3.32 |
| Pla2g5 | 1417814_at | -3.32 |
| Hal | 1418645_at | -3.30 |
| Prg4 | 1449824_at | -3.29 |
| Cpm | 1429413_at | -3.29 |
| Lipm | 1430550_at | -3.27 |
| OTTMUSG00000000971 | 1436530_at | -3.27 |
| 4732454E20Rik | 1436873_at | -3.26 |
| Dhcr24 | 1451895_a_at | -3.26 |
| Gm11554 | 1420599_at | -3.26 |
| Apoc1 | 1417561_at | -3.25 |
| Serpinb5 | 1438856_x_at | -3.25 |
| OTTMUSG00000005148 | 1455973_at | -3.23 |
| Aadac | 1448813_at | -3.23 |
| Krt15 | 1422667_at | -3.23 |
| 2310005G13Rik | 1430466_at | -3.23 |
| 2310057J16Rik | 1432464_a_at | -3.23 |
| Cxcl13 | 1417851_at | -3.21 |
| Camta1 | 1433972_at | -3.19 |
| Clip4 | 1427278_at | -3.18 |
| Mansc1 | 1423284_at | -3.17 |
| Ly6g6d | 1450774_at | -3.17 |
| Defb1 | 1419492_s_at | -3.17 |
| Il1f6 | 1418609_at | -3.16 |
| Pla2g2f | 1434852_at | -3.15 |
| Clip4 | 1437092_at | -3.13 |
| Klk7 | 1423542_at | -3.12 |
| Mgll | 1453836_a_at | -3.11 |
| Elovl4 | 1451308_at | -3.10 |
| Slc22a4 | 1417639_at | -3.10 |
| Tnfrsf19 | 1448147_at | -3.10 |
| Acacb | 1427052_at | -3.09 |
| Zbtb16 | 1439163_at | -3.07 |
| Cpm | 1453009_at | -3.07 |
| Krtap5-1 | 1450540_x_at | -3.06 |
| Slc5a1 | 1455431_at | -3.05 |
| Sgpp2 | 1457867_at | -3.05 |
| Rdh12 | 1424256_at | -3.04 |
| Ace2 | 1452138_a_at | -3.04 |
| Cyp2b10 | 1425645_s_at | -3.03 |
| Xkrx | 1454762_at | -3.03 |
| Alox8 | 1425376_at | -3.02 |
| Adh6a | 1429608_at | -3.01 |
| Gprc5d | 1420538_at | -3.01 |
| Adipoq | 1422651_at | -3.00 |
| Scgb1a1 | 1452543_a_at | -2.99 |
| Calm4 | 1450633_at | -2.99 |
| Angptl7 | 1451478_at | -2.98 |
| Krt82 | 1427719_s_at | -2.98 |
| Ctnnbip1 | 1431694_a_at | -2.98 |
| Rbm35a | 1454681_at | -2.97 |
| Tesc | 1418744_s_at | -2.97 |
| Grhl1 | 1424030_at | -2.97 |
| Efhd1 | 1448507_at | -2.96 |
| Gpld1 | 1418050_at | -2.96 |
| Ankrd35 | 1438474_at | -2.96 |
| Serpina9 | 1429285_at | -2.95 |
| Tnnt1 | 1419606_a_at | -2.95 |
| Atp6v1c2 | 1430306_a_at | -2.94 |
| S3-12 | 1418595_at | -2.93 |
| Acox2 | 1420673_a_at | -2.93 |
| Krtap5-5 | 1430728_at | -2.93 |
| Cfd | 1417867_at | -2.92 |
| Hoxd9 | 1419126_at | -2.92 |
| Tmem45b | 1424357_at | -2.92 |
| Rab27b | 1439610_at | -2.92 |
| Eif2c4 | 1453289_at | -2.92 |
| Hoxd10 | 1418606_at | -2.91 |
| 2010107G23Rik | 1424072_at | -2.90 |
| Acpp | 1419832_s_at | -2.90 |
| Scd3 | 1450956_at | -2.90 |
| Oplah | 1424359_at | -2.89 |
| Tyrp1 | 1415862_at | -2.87 |
| Acot1 | 1449065_at | -2.87 |
| Si | 1422523_at | -2.86 |
| Gnai1 | 1454959_s_at | -2.86 |
| Fam189a2 | 1435283_s_at | -2.86 |
| Krtap12-1 | 1450535_at | -2.85 |
| Il1f8 | 1425715_at | -2.85 |
| Sh3gl2 | 1418791_at | -2.84 |
| Ephb6 | 1418051_at | -2.83 |
| Gata3 | 1448886_at | -2.83 |
| Mb | 1451203_at | -2.82 |
| 2310014L17Rik | 1435572_at | -2.82 |
| Tcfap2b | 1423340_at | -2.81 |
| Cyp2b10 | 1422257_s_at | -2.81 |
| Hacl1 | 1449047_at | -2.78 |
| 2310040C09Rik | 1453184_at | -2.77 |
| Akr1c21 | 1451030_at | -2.77 |
| Lrp4 | 1426288_at | -2.76 |
| 4732473B16Rik | 1433877_at | -2.76 |
| Rps6ka6 | 1429759_at | -2.76 |
| Homer2 | 1424367_a_at | -2.75 |
| Serpinb5 | 1424623_at | -2.75 |
| BC054059 | 1424729_at | -2.73 |
| Lhx2 | 1418317_at | -2.73 |
| Slc15a1 | 1419343_at | -2.73 |
| Lyg2 | 1437899_at | -2.72 |
| Dlx5 | 1449863_a_at | -2.72 |
| Plcxd2 | 1455324_at | -2.72 |
| 9530008L14Rik | 1427957_at | -2.71 |
| Paqr5 | 1460459_at | -2.71 |
| Tnxb | 1450798_at | -2.70 |
| Scara5 | 1451204_at | -2.69 |
| Scd1 | 1415965_at | -2.68 |
| Prom2 | 1417047_at | -2.67 |
| Tcte3 | 1421682_a_at | -2.67 |
| 9930012K11Rik | 1433801_at | -2.66 |
| Bglap-rs1 | 1449880_s_at | -2.66 |
| Usp2 | 1417168_a_at | -2.66 |
| Pctp | 1420984_at | -2.65 |
| Gjb5 | 1449204_at | -2.65 |
| Bpil2 | 1437232_at | -2.65 |
| Wif1 | 1425425_a_at | -2.65 |
| Aldh6a1 | 1448104_at | -2.65 |
| Spink5 | 1430567_at | -2.64 |
| Ccdc64b | 1460051_at | -2.64 |
| Cds1 | 1456114_at | -2.64 |
| Gsta3 | 1423436_at | -2.63 |
| Fbp1 | 1448470_at | -2.63 |
| Higd1a | 1416481_s_at | -2.63 |
| Ptplb | 1437329_at | -2.63 |
| Spnb3 | 1452269_at | -2.62 |
| Inmt | 1418697_at | -2.61 |
| Asprv1 | 1452732_at | -2.61 |
| Dgat2 | 1422678_at | -2.60 |
| Pnpla3 | 1420655_at | -2.60 |
| Dhrs7 | 1426440_at | -2.60 |
| Gpm6a | 1456741_s_at | -2.59 |
| Foxp2 | 1438232_at | -2.59 |
| Tchhl1 | 1429944_at | -2.58 |
| Pank1 | 1418715_at | -2.58 |
| Eif2c4 | 1429779_at | -2.58 |
| Ctnnbip1 | 1417567_at | -2.57 |
| Pgrmc2 | 1452882_at | -2.56 |
| Cyp1b1 | 1416612_at | -2.56 |
| Pdzk1ip1 | 1455477_s_at | -2.56 |
| Plcxd2 | 1435462_at | -2.55 |
| Krt78 | 1438849_at | -2.55 |
| 2300002D11Rik | 1453008_at | -2.55 |
| Mreg | 1437250_at | -2.55 |
| Gnai1 | 1434440_at | -2.53 |
| 4632417N05Rik | 1427082_at | -2.53 |
| Irx4 | 1419539_at | -2.53 |
| Ddx26b | 1426832_at | -2.52 |
| Mycl1 | 1434777_at | -2.52 |
| Otub2 | 1417575_at | -2.51 |
| Bhlhb9 | 1428512_at | -2.51 |
| Slc40a1 | 1417061_at | -2.51 |
| Usp2 | 1417169_at | -2.51 |
| Pdcd4 | 1418840_at | -2.51 |
| Ces3 | 1435370_a_at | -2.50 |
| Pp11r | 1449937_at | -2.49 |
| Nrcam | 1434709_at | -2.49 |
| Plxdc2 | 1418912_at | -2.49 |
| 9530066K23Rik | 1441909_s_at | -2.49 |
| Mgst1 | 1415897_a_at | -2.49 |
| Pank1 | 1431028_a_at | -2.48 |
| Maob | 1434354_at | -2.48 |
| Acot1 | 1422997_s_at | -2.47 |
| Lrrc28 | 1433858_at | -2.46 |
| Asah3l | 1451355_at | -2.46 |
| Pdzk1ip1 | 1417689_a_at | -2.46 |
| Popdc3 | 1423856_at | -2.46 |
| Dlx3 | 1450475_at | -2.45 |
| Cobl | 1434917_at | -2.44 |
| Gstm5 | 1416842_at | -2.44 |
| Tgm3 | 1421355_at | -2.44 |
| Acsl1 | 1423883_at | -2.43 |
| 1190002N15Rik | 1433581_at | -2.43 |
| Tmem30b | 1433579_at | -2.43 |
| Dsc3 | 1434534_at | -2.43 |
| Pamci | 1427942_at | -2.43 |
| Casp14 | 1418748_at | -2.43 |
| 1100001G20Rik | 1434484_at | -2.43 |
| Vit | 1426231_at | -2.42 |
| Ptger3 | 1450344_a_at | -2.42 |
| Crct1 | 1453092_at | -2.41 |
| Tmprss13 | 1427477_at | -2.41 |
| Slc30a1 | 1436164_at | -2.41 |
| Tcfap2c | 1436392_s_at | -2.41 |
| Tmem16j | 1453041_at | -2.40 |
| Atp8a1 | 1433965_at | -2.40 |
| Tcfap2b | 1435670_at | -2.40 |
| F3 | 1417408_at | -2.39 |
| Trim29 | 1424162_at | -2.39 |
| Krt26 | 1436160_at | -2.38 |
| Myh7 | 1448553_at | -2.38 |
| Sh3yl1 | 1449579_at | -2.38 |
| Dkkl1 | 1417787_at | -2.38 |
| Crym | 1416776_at | -2.37 |
| Nrbp2 | 1424544_at | -2.37 |
| 2310007H09Rik | 1452889_at | -2.37 |
| Acpp | 1453943_a_at | -2.37 |
| Cyp2j6 | 1417952_at | -2.35 |
| BC055107 | 1434202_a_at | -2.35 |
| Akr1c18 | 1419136_at | -2.35 |
| Slc5a1 | 1419057_at | -2.35 |
| Serpina3c | 1421564_at | -2.35 |
| Yod1 | 1436976_a_at | -2.35 |
| Gjb3 | 1416715_at | -2.34 |
| Pparg | 1420715_a_at | -2.34 |
| Nudt17 | 1452995_at | -2.34 |
| Cldn1 | 1437932_a_at | -2.33 |
| Tbx1 | 1425779_a_at | -2.33 |
| Myoz2 | 1418769_at | -2.32 |
| Ly6d | 1416930_at | -2.31 |
| Hist2h2be | 1447854_s_at | -2.31 |
| Sema4g | 1449202_at | -2.31 |
| 2200002K05Rik | 1428400_at | -2.31 |
| Slc27a4 | 1424441_at | -2.31 |
| Krt75 | 1427378_at | -2.30 |
| Gjb4 | 1422179_at | -2.29 |
| Atp1a2 | 1443823_s_at | -2.28 |
| Sbsn | 1439630_x_at | -2.28 |
| Cyp17a1 | 1417017_at | -2.28 |
| 5730410E15Rik | 1438667_at | -2.28 |
| Cebpa | 1418982_at | -2.27 |
| Rdh12 | 1431010_a_at | -2.26 |
| Chdh | 1455435_s_at | -2.26 |
| Bmp7 | 1418910_at | -2.25 |
| Pp11r | 1449938_at | -2.25 |
| Anxa9 | 1431554_a_at | -2.25 |
| C030019I05Rik | 1436484_at | -2.24 |
| Rbp7 | 1449461_at | -2.24 |
| Cpeb3 | 1455372_at | -2.24 |
| Sbsn | 1459897_a_at | -2.24 |
| Egr2 | 1427683_at | -2.23 |
| Bmp4 | 1422912_at | -2.23 |
| Cited4 | 1425400_a_at | -2.23 |
| Fhit | 1425893_a_at | -2.23 |
| Kcna1 | 1437230_at | -2.22 |
| Hlf | 1434736_at | -2.22 |
| Chi3l1 | 1451537_at | -2.22 |
| Sh2d4a | 1453377_at | -2.22 |
| Egr2 | 1427682_a_at | -2.22 |
| Mllt4 | 1436303_at | -2.21 |
| Calml3 | 1418608_at | -2.21 |
| Myo5b | 1452298_a_at | -2.21 |
| BC031353 | 1436033_at | -2.21 |
| Myo6 | 1435559_at | -2.20 |
| Gjb6 | 1448397_at | -2.20 |
| Tuft1 | 1416689_at | -2.19 |
| Amy1 | 1417765_a_at | -2.19 |
| Cd36 | 1450884_at | -2.19 |
| Pdzd2 | 1435553_at | -2.19 |
| Gm94 | 1458367_at | -2.19 |
| Entpd2 | 1418259_a_at | -2.19 |
| Nrtn | 1449281_at | -2.18 |
| Il1f10 | 1451957_at | -2.18 |
| Tuba8 | 1419518_at | -2.18 |
| Mtap7 | 1421836_at | -2.18 |
| Il18 | 1417932_at | -2.17 |
| Nudt11 | 1426887_at | -2.17 |
| Rorc | 1425792_a_at | -2.17 |
| Ttc22 | 1457149_at | -2.16 |
| D9Ertd280e | 1456481_at | -2.16 |
| Rhbg | 1419134_at | -2.16 |
| Mapk13 | 1448871_at | -2.16 |
| Pxmp4 | 1422780_at | -2.16 |
| 1110067D22Rik | 1451313_a_at | -2.15 |
| Timp4 | 1423405_at | -2.15 |
| Celsr2 | 1435336_at | -2.15 |
| Aim1l | 1437813_at | -2.15 |
| Cd36 | 1423166_at | -2.15 |
| Paqr5 | 1456152_at | -2.14 |
| Gal3st1 | 1454078_a_at | -2.14 |
| Arl4a | 1431429_a_at | -2.13 |
| Btf3l4 | 1424074_at | -2.13 |
| Cbs | 1423844_s_at | -2.13 |
| BC038479 | 1433728_at | -2.13 |
| Ndrg2 | 1448154_at | -2.13 |
| Ahnak | 1428057_a_at | -2.12 |
| Slc6a8 | 1417116_at | -2.12 |
| Mal2 | 1427042_at | -2.12 |
| Mc5r | 1460723_at | -2.12 |
| Cma1 | 1449456_a_at | -2.11 |
| Rgn | 1448852_at | -2.11 |
| Aloxe3 | 1449237_at | -2.11 |
| Gm438 | 1446246_at | -2.10 |
| BB146404 | 1424680_at | -2.10 |
| Arrdc4 | 1424759_at | -2.10 |
| Sgpp1 | 1420822_s_at | -2.10 |
| Olig1 | 1416149_at | -2.10 |
| Gdpd2 | 1429076_a_at | -2.10 |
| Fetub | 1449555_a_at | -2.10 |
| Tex264 | 1425068_a_at | -2.09 |
| Ell3 | 1442514_a_at | -2.09 |
| Fam57b | 1452807_s_at | -2.09 |
| Klf4 | 1417394_at | -2.09 |
| Myl3 | 1427768_s_at | -2.09 |
| Slc10a6 | 1428776_at | -2.09 |
| Ly6g6e | 1429833_at | -2.08 |
| Ppil6 | 1453310_at | -2.08 |
| Alox12b | 1418266_at | -2.08 |
| Krtdap | 1434227_at | -2.08 |
| Chad | 1420569_at | -2.08 |
| Rasgrp1 | 1434295_at | -2.08 |
| 1810005K13Rik | 1444790_at | -2.08 |
| Lnx1 | 1455825_s_at | -2.07 |
| Cyp2b10 | 1451787_at | -2.07 |
| 1110067D22Rik | 1424318_at | -2.07 |
| Zfp711 | 1432750_at | -2.06 |
| Atp10b | 1453352_at | -2.06 |
| Tcfap2e | 1435205_at | -2.06 |
| BC038479 | 1433727_at | -2.06 |
| 5830404H04Rik | 1436344_at | -2.05 |
| Sbk1 | 1451190_a_at | -2.05 |
| Cyb561 | 1417507_at | -2.05 |
| Ovol1 | 1419051_at | -2.05 |
| Cxcl14 | 1418457_at | -2.04 |
| C79267 | 1435455_at | -2.03 |
| 9030619P08Rik | 1443889_at | -2.03 |
| Stx19 | 1425290_at | -2.03 |
| Heph | 1448696_at | -2.02 |
| Rnf208 | 1435105_at | -2.02 |
| Hoxd11 | 1450584_at | -2.02 |
| Cldn23 | 1424409_at | -2.02 |
| Il20rb | 1437876_at | -2.01 |
| Arl6ip2 | 1435594_at | -2.01 |
| Ccdc120 | 1428066_at | -2.01 |
| Kcnk7 | 1425437_a_at | -2.01 |
| Serpinb5 | 1421752_a_at | -2.01 |
| Sphk1 | 1451596_a_at | -2.01 |
| Acsl1 | 1450643_s_at | -2.01 |
| Pmp22 | 1417133_at | -2.01 |
| Scd3 | 1423366_at | -2.00 |
| Acad9 | 1429581_at | -2.00 |
| 4930422I07Rik | 1458491_at | -1.99 |
| Adh7 | 1450110_at | -1.99 |
| Pla2g4b | 1456047_at | -1.99 |
| Lin7a | 1435805_at | -1.99 |
| Ndn | 1455792_x_at | -1.99 |
| BC039632 | 1441991_at | -1.98 |
| Zfp750 | 1437469_at | -1.98 |
| Cd36 | 1450883_a_at | -1.98 |
| AI842396 | 1448034_at | -1.97 |
| 3110001I20Rik | 1450642_at | -1.97 |
| 2900006K08Rik | 1438122_at | -1.97 |
| Abca5 | 1434474_at | -1.97 |
| Tppp | 1452766_at | -1.97 |
| Tmem54 | 1417895_a_at | -1.96 |
| Klf4 | 1417395_at | -1.96 |
| 4732466D17Rik | 1438980_x_at | -1.96 |
| Tesc | 1418743_a_at | -1.96 |
| Dmd | 1448665_at | -1.96 |
| Ntn1 | 1454974_at | -1.95 |
| Bcl11b | 1435227_at | -1.95 |
| Dos | 1438370_x_at | -1.95 |
| Nov | 1426852_x_at | -1.95 |
| Osbpl1a | 1416823_a_at | -1.95 |
| Msx2 | 1449559_at | -1.95 |
| Sort1 | 1423362_at | -1.95 |
| Bnipl | 1420683_at | -1.95 |
| 1190002N15Rik | 1433582_at | -1.95 |
| Slc39a2 | 1443163_at | -1.95 |
| Smpd1 | 1448621_a_at | -1.95 |
| Cxcl13 | 1448859_at | -1.94 |
| Cyfip2 | 1428347_at | -1.94 |
| Boc | 1426869_at | -1.94 |
| Ssfa2 | 1423613_at | -1.93 |
| Slc44a1 | 1423865_at | -1.93 |
| Ube2e2 | 1424358_at | -1.93 |
| Vwa2 | 1438567_at | -1.93 |
| Clec4a3 | 1429954_at | 2.06 |
| Cttn | 1421313_s_at | 2.06 |
| Prc1 | 1423775_s_at | 2.07 |
| Lypla3 | 1422341_s_at | 2.07 |
| Mtap | 1424425_a_at | 2.07 |
| Marcksl1 | 1415922_s_at | 2.07 |
| Irg1 | 1427381_at | 2.07 |
| Ncf4 | 1418465_at | 2.07 |
| Slc16a3 | 1449005_at | 2.07 |
| Flrt2 | 1455096_at | 2.08 |
| Zfp260 | 1427831_s_at | 2.08 |
| Itga1 | 1455251_at | 2.08 |
| Efemp2 | 1417018_at | 2.08 |
| Hsp90aa1 | 1426645_at | 2.08 |
| Plat | 1415806_at | 2.08 |
| Itgax | 1419128_at | 2.08 |
| C1qb | 1417063_at | 2.08 |
| 1600029D21Rik | 1454254_s_at | 2.08 |
| Calm3 | 1426710_at | 2.09 |
| Plk1 | 1448191_at | 2.09 |
| Tmem2 | 1451458_at | 2.09 |
| Krt19 | 1417156_at | 2.09 |
| Snap23 | 1420896_at | 2.09 |
| Sfrs7 | 1424033_at | 2.10 |
| Msln | 1460238_at | 2.10 |
| 1200009F10Rik | 1429219_at | 2.10 |
| Il4ra | 1421034_a_at | 2.10 |
| Dusp9 | 1454737_at | 2.10 |
| Alcam | 1426300_at | 2.10 |
| Actn1 | 1428585_at | 2.10 |
| Chsy1 | 1434316_at | 2.10 |
| Csf3 | 1419427_at | 2.11 |
| Tcfec | 1419537_at | 2.11 |
| Emilin1 | 1416414_at | 2.11 |
| Atic | 1452811_at | 2.11 |
| Hs6st2 | 1450047_at | 2.11 |
| Wdr1 | 1437591_a_at | 2.11 |
| Sh3kbp1 | 1431592_a_at | 2.11 |
| Ccng1 | 1450016_at | 2.11 |
| Hmga1 | 1416184_s_at | 2.12 |
| Ctla2b | 1452352_at | 2.12 |
| 2310005E10Rik | 1453173_at | 2.12 |
| Pglyrp1 | 1449184_at | 2.13 |
| Pxn | 1426085_a_at | 2.13 |
| 2810417H13Rik | 1419153_at | 2.13 |
| Smc2 | 1429660_s_at | 2.13 |
| BC025076 | 1451583_a_at | 2.13 |
| Pdia4 | 1416497_at | 2.13 |
| Vnn1 | 1447845_s_at | 2.13 |
| Padi4 | 1422760_at | 2.13 |
| Tnfrsf12a | 1418571_at | 2.13 |
| Ccnb1 | 1419943_s_at | 2.14 |
| Ccna2 | 1417911_at | 2.14 |
| Cxcl16 | 1449195_s_at | 2.15 |
| Tnfrsf22 | 1422039_at | 2.15 |
| Cep55 | 1453683_a_at | 2.15 |
| Ccng1 | 1450017_at | 2.15 |
| Cyp4f18 | 1419219_at | 2.15 |
| Fut8 | 1460319_at | 2.16 |
| Has2 | 1418678_at | 2.16 |
| Gzmd | 1420343_at | 2.16 |
| Vcam1 | 1415989_at | 2.16 |
| Dmrta2 | 1441107_at | 2.16 |
| Trim30 | 1451860_a_at | 2.17 |
| Lasp1 | 1460173_at | 2.17 |
| Gprc5b | 1424613_at | 2.17 |
| Gprc5b | 1451411_at | 2.17 |
| Stom | 1449341_a_at | 2.17 |
| Cald1 | 1424769_s_at | 2.17 |
| Csf3r | 1418806_at | 2.18 |
| Lamp3 | 1417057_a_at | 2.18 |
| Mmp14 | 1448383_at | 2.18 |
| Aif1 | 1418204_s_at | 2.18 |
| Fcgr2b | 1435477_s_at | 2.19 |
| Tmepai | 1422705_at | 2.19 |
| AI467606 | 1433466_at | 2.19 |
| Dclk1 | 1451289_at | 2.19 |
| Nid1 | 1448469_at | 2.19 |
| Runx1 | 1440878_at | 2.20 |
| Tnfaip2 | 1438855_x_at | 2.20 |
| Bcl2l15 | 1452614_at | 2.20 |
| Sec61a1 | 1416190_a_at | 2.20 |
| Reg1 | 1415905_at | 2.20 |
| 2410014A08Rik | 1436509_at | 2.20 |
| Vcl | 1416157_at | 2.20 |
| Krt6b | 1422588_at | 2.21 |
| Gsr | 1421817_at | 2.21 |
| Car4 | 1448949_at | 2.21 |
| Cd38 | 1433741_at | 2.21 |
| Cxcl1 | 1457644_s_at | 2.21 |
| Ccr1 | 1419610_at | 2.22 |
| Eps8 | 1422824_s_at | 2.22 |
| Lcp1 | 1415983_at | 2.22 |
| Kif20a | 1449207_a_at | 2.22 |
| Ceacam1 | 1427711_a_at | 2.22 |
| Lrrc17 | 1429679_at | 2.22 |
| Prc1 | 1423774_a_at | 2.22 |
| Ddah1 | 1455400_at | 2.22 |
| Plek | 1417523_at | 2.23 |
| Aurka | 1424511_at | 2.23 |
| Eps8 | 1422823_at | 2.23 |
| Srgn | 1417426_at | 2.24 |
| Lasp1 | 1448207_at | 2.24 |
| Kif22 | 1451128_s_at | 2.24 |
| Ddah1 | 1454995_at | 2.24 |
| Itgb1 | 1426918_at | 2.24 |
| Enpp1 | 1459546_s_at | 2.24 |
| Ccbe1 | 1437385_at | 2.24 |
| Flrt2 | 1438702_at | 2.24 |
| Ptges | 1449449_at | 2.25 |
| Plscr1 | 1429527_a_at | 2.25 |
| Mnda | 1452348_s_at | 2.25 |
| Kpnb1 | 1434357_a_at | 2.25 |
| Msr2 | 1448891_at | 2.25 |
| Apob48r | 1420382_at | 2.26 |
| D0H4S114 | 1436736_x_at | 2.26 |
| Ube2s | 1416726_s_at | 2.26 |
| Gfpt1 | 1428715_at | 2.26 |
| Tpm3 | 1427567_a_at | 2.26 |
| Col5a1 | 1434479_at | 2.27 |
| Ccl9 | 1448898_at | 2.27 |
| Hif1a | 1448183_a_at | 2.27 |
| Inhba | 1422053_at | 2.27 |
| Vnn1 | 1418486_at | 2.27 |
| Ifitm1 | 1424254_at | 2.27 |
| Pi15 | 1421403_at | 2.27 |
| Loxl2 | 1431004_at | 2.28 |
| Lpp | 1438271_at | 2.28 |
| Bst1 | 1449453_at | 2.28 |
| Smc2 | 1429658_a_at | 2.28 |
| Plscr1 | 1453181_x_at | 2.29 |
| Sox2 | 1416967_at | 2.29 |
| Cap1 | 1417462_at | 2.29 |
| Tnfrsf12a | 1418572_x_at | 2.29 |
| Obfc2a | 1452203_at | 2.29 |
| Tmod3 | 1423089_at | 2.30 |
| Flrt2 | 1456874_at | 2.30 |
| Ncapg | 1429172_a_at | 2.30 |
| Ctla2a | 1416811_s_at | 2.30 |
| D2Ertd750e | 1420081_s_at | 2.30 |
| D0H4S114 | 1450839_at | 2.31 |
| Bcl2a1b | 1419004_s_at | 2.31 |
| 4632417K18Rik | 1422628_at | 2.31 |
| Pdlim5 | 1450786_x_at | 2.31 |
| Ccl8 | 1419684_at | 2.32 |
| Cd53 | 1448617_at | 2.32 |
| 2810004N23Rik | 1448947_at | 2.32 |
| Chst11 | 1428902_at | 2.32 |
| Itgb1 | 1452545_a_at | 2.33 |
| Adcy7 | 1450065_at | 2.33 |
| Steap1 | 1451532_s_at | 2.34 |
| Mtpn | 1420472_at | 2.34 |
| Hmmr | 1450157_a_at | 2.34 |
| Grem1 | 1425357_a_at | 2.34 |
| Hsp90b1 | 1438040_a_at | 2.35 |
| Lamb1-1 | 1424114_s_at | 2.35 |
| Scd2 | 1415824_at | 2.35 |
| Rhoc | 1448605_at | 2.35 |
| 2010002N04Rik | 1423306_at | 2.35 |
| Rai14 | 1417400_at | 2.35 |
| E2f7 | 1437187_at | 2.35 |
| Trp53 | 1426538_a_at | 2.35 |
| Rras2 | 1448689_at | 2.36 |
| Actn1 | 1452415_at | 2.38 |
| Col5a2 | 1422437_at | 2.38 |
| Trp53 | 1427739_a_at | 2.38 |
| Gzmd | 1420344_x_at | 2.39 |
| Ptges | 1449450_at | 2.39 |
| Acsl4 | 1451828_a_at | 2.39 |
| Ncam1 | 1426865_a_at | 2.39 |
| Mtap | 1424426_at | 2.39 |
| Slco2a1 | 1450032_at | 2.40 |
| Col5a1 | 1416741_at | 2.41 |
| Pdlim5 | 1422861_s_at | 2.42 |
| Ms4a6d | 1419599_s_at | 2.42 |
| Fbln2 | 1423407_a_at | 2.42 |
| Myadm | 1439389_s_at | 2.43 |
| Cnn2 | 1450981_at | 2.43 |
| Col5a1 | 1416740_at | 2.43 |
| Cenpe | 1439040_at | 2.44 |
| St3gal4 | 1425668_a_at | 2.44 |
| Foxc2 | 1416693_at | 2.44 |
| Cdc2a | 1448314_at | 2.44 |
| Ier5l | 1419066_at | 2.44 |
| Bub1 | 1424046_at | 2.45 |
| Bmp1 | 1427457_a_at | 2.45 |
| Gprc5a | 1437486_at | 2.45 |
| Rrm2 | 1416120_at | 2.46 |
| Ccnb1 | 1416076_at | 2.47 |
| Hdlbp | 1449615_s_at | 2.47 |
| Oasl2 | 1453196_a_at | 2.47 |
| Krt4 | 1418735_at | 2.47 |
| Fblim1 | 1418569_at | 2.49 |
| D2Ertd750e | 1423463_a_at | 2.49 |
| Rrm1 | 1448127_at | 2.49 |
| Sox4 | 1419157_at | 2.50 |
| Tmsb10 | 1436902_x_at | 2.50 |
| Fosl1 | 1417488_at | 2.50 |
| Serpinb9g | 1418423_s_at | 2.51 |
| Fcgr1 | 1417876_at | 2.51 |
| Ccnd1 | 1417420_at | 2.51 |
| Angptl2 | 1421002_at | 2.52 |
| Cd300lf | 1427994_at | 2.52 |
| Hdlbp | 1415988_at | 2.53 |
| Myadm | 1423321_at | 2.53 |
| Ccl3 | 1419561_at | 2.53 |
| Lyn | 1425598_a_at | 2.53 |
| Cxcr4 | 1448710_at | 2.54 |
| Ly6e | 1453304_s_at | 2.54 |
| Slpi | 1448377_at | 2.54 |
| Pla1a | 1417785_at | 2.54 |
| Sfrp2 | 1448201_at | 2.55 |
| B4galnt1 | 1418655_at | 2.55 |
| Lgals1 | 1455439_a_at | 2.56 |
| Gusb | 1430332_a_at | 2.56 |
| Vsnl1 | 1420955_at | 2.56 |
| Pla2g7 | 1430700_a_at | 2.56 |
| Ms4a7 | 1424754_at | 2.57 |
| Bcat1 | 1450871_a_at | 2.57 |
| Plod2 | 1416686_at | 2.57 |
| Clec4d | 1420804_s_at | 2.57 |
| Clec5a | 1421366_at | 2.57 |
| Ddah1 | 1429298_at | 2.58 |
| Cep55 | 1452242_at | 2.58 |
| Il2rg | 1416296_at | 2.58 |
| Il4ra | 1423996_a_at | 2.59 |
| Tnfrsf22 | 1426095_a_at | 2.59 |
| Lman1 | 1428129_at | 2.60 |
| Dok1 | 1417790_at | 2.60 |
| Tes | 1424246_a_at | 2.60 |
| Hbegf | 1418349_at | 2.61 |
| P4ha1 | 1426519_at | 2.61 |
| Eps8 | 1425733_a_at | 2.61 |
| Cldn7 | 1448393_at | 2.62 |
| Fst | 1434458_at | 2.63 |
| Stom | 1419098_at | 2.64 |
| Col5a2 | 1450625_at | 2.64 |
| Mlstd2 | 1453550_a_at | 2.64 |
| Aldh1a3 | 1427395_a_at | 2.65 |
| Actn1 | 1427385_s_at | 2.66 |
| Trem1 | 1460282_at | 2.66 |
| Mtap | 1451346_at | 2.66 |
| Slc25a24 | 1427483_at | 2.67 |
| Il2rg | 1416295_a_at | 2.67 |
| Met | 1434447_at | 2.67 |
| Hsp110 | 1423566_a_at | 2.68 |
| Cdkn2a | 1450140_a_at | 2.68 |
| Fcgr2b | 1451941_a_at | 2.71 |
| Il6 | 1450297_at | 2.71 |
| Ms4a6d | 1419598_at | 2.71 |
| Lamb1-1 | 1424113_at | 2.71 |
| Clec4n | 1425951_a_at | 2.71 |
| Adamts4 | 1452595_at | 2.74 |
| Ube2c | 1452954_at | 2.75 |
| Oaf | 1424086_at | 2.75 |
| Mal | 1432558_a_at | 2.76 |
| Antxr2 | 1426708_at | 2.76 |
| Tgfb1 | 1420653_at | 2.76 |
| Steap2 | 1428636_at | 2.77 |
| Col1a1 | 1423669_at | 2.77 |
| Clec7a | 1420699_at | 2.77 |
| Elk3 | 1448797_at | 2.78 |
| Loxl3 | 1418269_at | 2.78 |
| Serpinh1 | 1450843_a_at | 2.79 |
| Lamb1-1 | 1451241_at | 2.81 |
| Pcdh7 | 1456214_at | 2.81 |
| Cald1 | 1424770_at | 2.82 |
| Serpinb9 | 1439790_at | 2.82 |
| Stom | 1419099_x_at | 2.83 |
| Rab31 | 1431691_a_at | 2.83 |
| Fpr-rs2 | 1422953_at | 2.83 |
| Aldh1a3 | 1417642_at | 2.83 |
| Lgals1 | 1419573_a_at | 2.86 |
| Msr1 | 1448061_at | 2.87 |
| Adam12 | 1421171_at | 2.87 |
| Loxl2 | 1452436_at | 2.87 |
| Slfn4 | 1427102_at | 2.88 |
| Obfc2a | 1460521_a_at | 2.90 |
| Itgam | 1422046_at | 2.91 |
| Wnt5a | 1448818_at | 2.92 |
| Stra6 | 1422723_at | 2.93 |
| Ywhag | 1420816_at | 2.94 |
| Ceacam1 | 1452532_x_at | 2.98 |
| Dsc2 | 1421156_a_at | 3.00 |
| Enpp1 | 1419276_at | 3.00 |
| Clec4e | 1420330_at | 3.00 |
| Rras2 | 1417398_at | 3.01 |
| Mmp14 | 1416572_at | 3.01 |
| Rai14 | 1417401_at | 3.02 |
| Mlkl | 1429570_at | 3.02 |
| Tes | 1460378_a_at | 3.03 |
| Cthrc1 | 1452968_at | 3.04 |
| Ceacam1 | 1460682_s_at | 3.05 |
| Fosl1 | 1417487_at | 3.05 |
| Cxcl1 | 1419209_at | 3.07 |
| Dusp6 | 1415834_at | 3.09 |
| Ifi202b | 1457666_s_at | 3.13 |
| Chi3l4 | 1425450_at | 3.13 |
| Serpine1 | 1419149_at | 3.15 |
| Cd14 | 1417268_at | 3.16 |
| Ccr1 | 1419609_at | 3.17 |
| Crisp1 | 1416325_at | 3.19 |
| Csrp2 | 1420731_a_at | 3.20 |
| Mtap | 1451345_at | 3.22 |
| Wisp1 | 1448593_at | 3.23 |
| Fn1 | 1426642_at | 3.24 |
| Cpxm1 | 1448901_at | 3.26 |
| A330021E22Rik | 1455859_at | 3.26 |
| Adam8 | 1416871_at | 3.28 |
| Klra18 | 1426127_x_at | 3.28 |
| Vcam1 | 1448162_at | 3.29 |
| Tubb6 | 1416431_at | 3.29 |
| Col8a1 | 1455627_at | 3.30 |
| Tnfrsf23 | 1442590_at | 3.31 |
| Plaur | 1452521_a_at | 3.32 |
| Ceacam1 | 1422123_s_at | 3.32 |
| Msn | 1450379_at | 3.32 |
| Glipr1 | 1424927_at | 3.33 |
| Tnfrsf23 | 1422101_at | 3.33 |
| Serpinb9 | 1422601_at | 3.33 |
| Pitx1 | 1419514_at | 3.34 |
| Pyhin1 | 1435331_at | 3.35 |
| Phlda1 | 1418835_at | 3.40 |
| Ccnd1 | 1417419_at | 3.40 |
| Plod2 | 1416687_at | 3.42 |
| Tnc | 1416342_at | 3.43 |
| Anxa3 | 1460330_at | 3.45 |
| Gzme | 1450171_x_at | 3.46 |
| Ceacam1 | 1425675_s_at | 3.47 |
| Il1rl1 | 1422317_a_at | 3.47 |
| Col8a1 | 1418440_at | 3.48 |
| Fst | 1421365_at | 3.51 |
| Chi3l4 | 1425451_s_at | 3.54 |
| Igf2bp2 | 1437103_at | 3.55 |
| Steap1 | 1424938_at | 3.55 |
| Casp3 | 1426165_a_at | 3.56 |
| Upp1 | 1448562_at | 3.58 |
| Runx1 | 1422864_at | 3.59 |
| Tfpi2 | 1418547_at | 3.60 |
| Sh2d5 | 1436100_at | 3.61 |
| Wisp1 | 1448594_at | 3.61 |
| C1qtnf3 | 1422606_at | 3.71 |
| Pthlh | 1422324_a_at | 3.72 |
| Saa3 | 1450826_a_at | 3.72 |
| Kcnn4 | 1435945_a_at | 3.74 |
| Areg | 1421134_at | 3.75 |
| Timp1 | 1460227_at | 3.82 |
| Rbp1 | 1448754_at | 3.87 |
| Parva | 1416818_at | 3.89 |
| Psca | 1451258_at | 3.90 |
| Serpinb6b | 1422804_at | 3.91 |
| Il24 | 1426181_a_at | 3.92 |
| Sprr3 | 1422401_at | 3.93 |
| Kcnn4 | 1421038_a_at | 3.94 |
| Fscn1 | 1416514_a_at | 3.98 |
| Adam12 | 1421172_at | 4.01 |
| Cald1 | 1424768_at | 4.03 |
| Pthlh | 1427527_a_at | 4.04 |
| Il1b | 1449399_a_at | 4.06 |
| Mmp9 | 1416298_at | 4.06 |
| Crabp1 | 1448326_a_at | 4.10 |
| Pitx1 | 1449488_at | 4.13 |
| Ptgs2 | 1417263_at | 4.16 |
| Mmp9 | 1448291_at | 4.17 |
| Mmp3 | 1418945_at | 4.26 |
| Ltf | 1450009_at | 4.27 |
| Mmp10 | 1420450_at | 4.27 |
| Krt13 | 1422454_at | 4.39 |
| Gzme | 1421227_at | 4.39 |
| Igf2bp2 | 1439764_s_at | 4.72 |
| Prkg2 | 1435460_at | 4.86 |
| Ppbp | 1418480_at | 4.87 |
| Krt8 | 1423691_x_at | 4.93 |
| Mmp12 | 1449153_at | 4.94 |
| Krt8 | 1420647_a_at | 4.95 |
| Sprr2f | 1449833_at | 5.05 |
| Prkg2 | 1435162_at | 5.14 |
| Prl2c2 | 1427760_s_at | 5.77 |
| Hmga2 | 1450780_s_at | 5.90 |
| Hmga2 | 1422851_at | 5.96 |
| Krt18 | 1448169_at | 6.14 |
| Cxcl3 | 1438148_at | 6.23 |
| Hmga2 | 1450781_at | 6.31 |
| Mmp13 | 1417256_at | 6.67 |

**Table S4**

Perturbation eQTL

**"Obs. *P* value"**: Observed P value from regression

**"Perm *P* value**": P value calculated from 1000 permutations

"**Mean Fold FF**": Mean fold-change of this probe between matched skin and carcinoma samples when a mouse was homozygous at this locus

**"Mean Fold FS**": Mean fold-change of this probe between matched skin and carcinoma samples when a mouse was heterozygous at this locus

"**Mean Skin**": Mean value of this probe in all skin samples (steady-state)

"**Mean Carcinoma**": Mean value of this probe in all carcinoma samples (steady-state)

| **Gene** | **Probe** | **SNP** | **Obs. *P***  **value** | **Perm *P* value** | **Mean Fold**  **FF** | **Mean Fold**  **FS** | **Mean Skin** | **Mean**  **carcinoma** |
| --- | --- | --- | --- | --- | --- | --- | --- | --- |
| Rab11fip5 | 1427405_s_at | E01.008.129_10 | 1.30E-05 | 0.001 | 0.601 | -0.0571 | 6.85 | 6.95 |
| Atp2c1 | 1437738_at | E01.008.129_10 | 1.90E-05 | 0 | 0.222 | -0.247 | 8.55 | 8.91 |
| Anxa4 | 1424176_a_at | E01.033.082_10 | 2.40E-06 | 0 | 1.41 | 0.729 | 8.08 | 9.3 |
| Pam | 1418908_at | E01.086.274_10 | 2.50E-06 | 0 | -0.242 | -1.01 | 8.41 | 7.52 |
| Ezh1 | 1449023_a_at | E01.169.742_10 | 1.40E-06 | 0.001 | -0.472 | -0.933 | 7.5 | 7.24 |
| Gpatch1 | 1419013_at | E02.056.057_10 | 1.50E-05 | 0.001 | -0.972 | -0.609 | 7.16 | 6.55 |
| Nckipsd | 1418975_at | E02.066.918_10 | 1.80E-05 | 0 | -1.08 | -0.651 | 6.87 | 6.06 |
| 0610025P10Rik | 1430417_s_at | E02.112.734_10 | 5.80E-06 | 0 | -0.893 | -0.461 | 7.04 | 6.18 |
| Zfp110 | 1437237_x_at | E02.122.101_10 | 7.00E-06 | 0 | -0.608 | -0.122 | 7.76 | 7.57 |
| Hnrnpa2b1 | 1420365_a_at | E03.100.082_10 | 6.80E-06 | 0.001 | 1.19 | 0.752 | 9.71 | 10.8 |
| A230046K03Rik | 1436353_at | E03.140.010_10 | 2.20E-05 | 0.001 | 1.19 | 0.738 | 6.47 | 7.58 |
| Thoc1 | 1424641_a_at | E03.158.424_10 | 1.10E-05 | 0.001 | -0.0359 | -0.618 | 7.97 | 7.59 |
| Cpne3 | 1452814_at | E04.043.559_10 | 5.10E-08 | 0 | 1.85 | 0.986 | 6.18 | 7.85 |
| Eif5a | 1437859_x_at | E05.002.624_10 | 4.70E-06 | 0 | 1.09 | 0.576 | 9.56 | 10.8 |
| Fkbp6 | 1425101_a_at | E05.002.624_10 | 7.10E-06 | 0 | -0.344 | 0.0414 | 5.21 | 5.33 |
| Phgdh | 1426657_s_at | E05.002.624_10 | 1.30E-05 | 0 | 0.504 | -0.669 | 7.23 | 7.55 |
| Ece2 | 1423358_at | E05.023.497_10 | 1.80E-05 | 0.001 | 0.915 | 0.424 | 6.12 | 7.15 |
| Ddx51 | 1428728_at | E05.103.695_10 | 4.00E-06 | 0.001 | 0.567 | 0.191 | 6.11 | 6.55 |
| Iigp1 | 1419043_a_at | E06.001.969_10 | 2.10E-05 | 0.001 | 1.53 | -0.0591 | 5.71 | 6.42 |
| Ubtd2 | 1455862_at | E06.001.969_10 | 2.70E-05 | 0.001 | 0.346 | -0.182 | 7.17 | 7.39 |
| Gpr146 | 1454685_at | E06.032.288_10 | 1.60E-06 | 0.001 | 0.16 | -0.867 | 5.66 | 5.14 |
| Lrrc33 | 1451174_at | E06.032.288_10 | 3.10E-06 | 0 | 1.67 | 0.711 | 5.34 | 7.15 |
| Pip4k2a | 1449404_at | E06.032.288_10 | 4.80E-06 | 0.001 | 1.16 | 0.563 | 6.72 | 7.66 |
| Csf2rb2 | 1449360_at | E06.032.288_10 | 4.90E-06 | 0 | 1.01 | 0.167 | 6.1 | 6.95 |
| Arhgef6 | 1429012_at | E06.032.288_10 | 4.90E-06 | 0 | 0.0663 | -0.836 | 6.95 | 6.62 |
| Arrb1 | 1460444_at | E06.032.288_10 | 6.70E-06 | 0 | -0.471 | -1.1 | 6.4 | 6 |
| Tspyl4 | 1424029_at | E06.032.288_10 | 1.50E-05 | 0 | -0.717 | -1.62 | 6.56 | 5.11 |
| Snx10 | 1431055_a_at | E06.046.553_10 | 1.60E-05 | 0.001 | 1.41 | 0.369 | 6.28 | 7.6 |
| Tuba3a | 1448296_x_at | E06.049.290_10 | 1.90E-05 | 0 | 0.302 | 0.639 | 5.48 | 6.89 |
| Apoc2 | 1418069_at | E07.002.028_10 | 4.70E-12 | 0 | 1.01 | -0.764 | 5.43 | 5.73 |
| Eif3s10 | 1448425_at | E07.079.411_10 | 4.20E-08 | 0 | 0.358 | 0.82 | 9.62 | 10.7 |
| Hsp90aa1 | 1426645_at | E07.079.411_10 | 2.10E-06 | 0 | 1.73 | 2.39 | 9.1 | 11.6 |
| Rif1 | 1437179_at | E07.079.411_10 | 4.00E-06 | 0.001 | 0.116 | 0.856 | 6.14 | 7.15 |
| Mpi | 1451540_at | E07.079.411_10 | 5.00E-06 | 0 | 0.221 | 0.693 | 5.49 | 6.33 |
| Ttc14 | 1426544_a_at | E07.079.411_10 | 1.30E-05 | 0 | 0.506 | 1.09 | 5.72 | 6.92 |
| Npm1 | 1432416_a_at | E07.079.411_10 | 1.60E-05 | 0.001 | 0.707 | 1.3 | 10.8 | 12.2 |
| Rbbp8 | 1427062_at | E07.079.411_10 | 2.60E-05 | 0.001 | 0.176 | 0.848 | 5.65 | 7.23 |
| Ash1l | 1450071_at | E07.097.360_10 | 8.40E-06 | 0 | 0.027 | 0.577 | 6.03 | 7.03 |
| Ppp2cb | 1421823_a_at | E08.020.455_10 | 1.10E-05 | 0.001 | 1.41 | 0.817 | 8.47 | 9.97 |
| Tmem189 | 1424411_at | E08.038.758_10 | 4.80E-06 | 0 | -0.666 | -0.156 | 7.66 | 7.37 |
| Pscdbp | 1451206_s_at | E09.034.366_10 | 5.00E-06 | 0.001 | 0.849 | 2.08 | 6.05 | 7.97 |
| Pscdbp | 1435697_a_at | E09.034.366_10 | 6.50E-06 | 0 | 0.644 | 1.86 | 6.94 | 8.37 |
| Adcy3 | 1421960_at | E09.034.366_10 | 1.80E-05 | 0.001 | -0.768 | 0.00414 | 6.91 | 7.17 |
| BC038286 | 1452164_at | E09.094.015_10 | 1.70E-05 | 0.001 | -0.851 | -0.494 | 8.05 | 7.67 |
| H2-Ke6 | 1454987_a_at | E10.102.359_10 | 7.40E-06 | 0.001 | -1.11 | -1.51 | 7.11 | 5.78 |
| 6030443O07Rik | 1438233_at | E10.106.427_10 | 1.50E-05 | 0.001 | -0.227 | -0.7 | 7.67 | 7.24 |
| Ace2 | 1425103_at | E12.023.118_10 | 1.40E-05 | 0.001 | -4.07 | -3.18 | 8.44 | 5.84 |
| Pik3ip1 | 1428332_at | E12.023.118_10 | 2.80E-05 | 0.001 | -0.559 | -1.25 | 6.91 | 6.15 |
| Cxcl16 | 1449195_s_at | E12.073.179_10 | 9.20E-06 | 0.001 | 1.74 | 2.53 | 5.6 | 7.84 |
| Zfp386 | 1451146_at | E12.092.013_10 | 5.70E-06 | 0 | 0.421 | -0.311 | 5.98 | 6.58 |
| Cxxc1 | 1452221_a_at | E13.053.052_10 | 2.50E-06 | 0 | -0.387 | -0.047 | 8.17 | 7.62 |
| Klhdc3 | 1415991_a_at | E13.053.052_10 | 9.10E-06 | 0.001 | -0.775 | -0.343 | 9.93 | 9.65 |
| Dapk1 | 1426915_at | E13.056.787_10 | 7.80E-06 | 0.001 | -1.35 | -0.394 | 8.03 | 7.54 |
| Klhdc3 | 1454747_a_at | E13.056.787_10 | 1.80E-05 | 0.001 | -0.811 | -0.394 | 9.32 | 9.01 |
| Add3 | 1426574_a_at | E19.055.858_10 | 1.20E-05 | 0.001 | -0.364 | 0.448 | 6.79 | 7.13 |

**REFERENCES**

1. Quigley D, To M, Pérez-Losada J, Pelorosso F, Mao J, Nagase H, Ginzinger D, Balmain A: **Genetic architecture of mouse skin inflammation and tumour susceptibility.** *Nature* 2009, **458:**505-508.

2. Ciobanu DC, Lu L, Mozhui K, Wang X, Jagalur M, Morris JA, Taylor WL, Dietz K, Simon P, Williams RW: **Detection, validation, and downstream analysis of allelic variation in gene expression.** *Genetics* 2010, **184:**119-128.
